# DNA barcoding aids in generating a preliminary checklist of the lichens and allied fungi of Calvert Island, British Columbia: Results from the 2018 Hakai Terrestrial BioBlitz

**DOI:** 10.3897/BDJ.12.e120292

**Published:** 2024-02-28

**Authors:** Richard Troy McMullin, Andrew D. F. Simon, Irwin M. Brodo, Sara B. Wickham, Philip Bell-Doyon, Maria Kuzmina, Brian M. Starzomski

**Affiliations:** 1 Canadian Museum of Nature, PO Box 3443, Station D, Ottawa, Ontario, K1P 6P4, Canada Canadian Museum of Nature, PO Box 3443, Station D Ottawa, Ontario, K1P 6P4 Canada; 2 School of Environmental Studies, University of Victoria, Victoria, British Columbia, V8P 5C2, Canada School of Environmental Studies, University of Victoria Victoria, British Columbia, V8P 5C2 Canada; 3 Hakai Institute, PO Box 309, Heriot Bay, British Columbia, VOP 1H0, Canada Hakai Institute, PO Box 309 Heriot Bay, British Columbia, VOP 1H0 Canada; 4 Department of Biology, Université Laval, Québec, Québec, G1V 0A6, Canada Department of Biology, Université Laval Québec, Québec, G1V 0A6 Canada; 5 Centre for Biodiversity Genomics, Biodiversity Institute of Ontario, University of Guelph, Guelph, Ontario, N1G 2W1, Canada Centre for Biodiversity Genomics, Biodiversity Institute of Ontario, University of Guelph Guelph, Ontario, N1G 2W1 Canada

**Keywords:** biogeography, Calicioids, Central Coast Regional District, Great Bear Rainforest, ITS, Pacific Northwest

## Abstract

**Background:**

Bioblitzes are a tool for the rapid appraisal of biodiversity and are particularly useful in remote and understudied regions and for understudied taxa. Lichens are an example of an often overlooked group, despite being widespread in virtually all terrestrial ecosystems and having many important ecological functions.

**New information:**

We report the lichens and allied fungi collected during the 2018 terrestrial bioblitz conducted on Calvert Island on the Central Coast of British Columbia, Canada. We identified 449 specimens belonging to 189 species in 85 genera, increasing the total number of species known from Calvert Island to 194, and generated Internal Transcribed Spacer (ITS) sequences for 215 specimens from 121 species. *Bryoriafurcellata*, *Chaenothecopsislecanactidis* and *C.nigripunctata* were collected for the first time in British Columbia. We also found *Pseudocyphellariarainierensis*, which is listed as Special Concern on the federal Species at Risk Act, and other rarely reported species in British Columbia including *Opegraphasphaerophoricola*, *Protomicarealimosa*, *Raesaeneniahuuskonenii* and *Sareadifformis*. We demonstrate that DNA barcoding improves the scope and accuracy of expert-led bioblitzes by facilitating the detection of cryptic species and allowing for consistent identification of chemically and morphologically overlapping taxa. Despite the spatial and temporal limitations of our study, the results highlight the value of intact forest ecosystems on the Central Coast of British Columbia for lichen biodiversity, education and conservation.

## Introduction

Bioblitzes are a tool for the rapid appraisal of biodiversity in a region (e.g. Anderson et al. (2012), Telfer et al. (2015), McMullin et al. (2018)). Scientists, naturalists and other volunteers assemble at a target site and, over a short period of time, attempt to find as many species as possible (Censky 2001, iNaturalist 2022). Bioblitzes are usually held over one 24-hour period but can often extend over a longer time to intensively sample important or remote locations. Integrating DNA barcoding into identification procedures can further expand the scope and scale of bioblitzes (Telfer et al. 2015). Bioblitzes are also important for understanding species in space and time particularly because of our changing climate and declining global biodiversity. A series of extended bioblitzes were recently held at the Hakai Institute on Calvert Island, in Haíłzaqv and Wuikinuxv territories on the Central Coast of British Columbia, Canada. The Hakai Institute is a British Columbia-based not-for-profit science and ecology organisation dedicated to the coordination of collaborative, multidisciplinary, field-based research and long-term monitoring of coastal ecosystems (Hakai Institute 2023). One of its field stations is located on Calvert Island, within the Hakai Lúxvbálís Conservancy, which is one of the largest protected areas in the BC Parks and Protected Areas System (Government of British Columbia 2023). To support their research initiative on the understanding of biodiversity of the Central Coast, the Hakai Institute organised three bioblitzes on Calvert Island: two marine ones in 2017 and 2018 and a terrestrial one in 2018. Here, we present results from the 2018 terrestrial bioblitz for lichens and their allied fungi.

Lichens are a polyphyletic group that includes a symbiosis between a mycobiont and at least one photobiont (an alga and/or a cyanobacterium) (Grimm et al. 2021, Allen and Lendemer 2022, Spribille et al. 2022, Sanders 2024). Due to their dependence on and/or resemblance to lichens, some taxa of non‑lichenised “allied” fungi (e.g. lichenicolous fungi) have traditionally been included in lichen studies (McMullin et al. 2017b, Spribille et al. 2020, Esslinger 2021, Bell-Doyon 2023), as they are here in our work. Lichens have many important ecological functions including aiding in soil formation (Adamo and Violante 2000), helping to stabilise soil (Belnap and Gillette 1998, Eldridge and Leys 2003), contributing to nutrient cycling (Pike 1978, Knops et al. 1991) and they provide camouflage, habitat and food for many organisms (e.g. Pettersson et al. (1995), Hansell (1996), Webber et al. (2022), Miranda-González et al. (2023)). Moreover, lichen communities are used as indicators of atmospheric pollution (Hawksworth and Rose 1970, Richardson 1988, McMullin et al. 2016, McMullin et al. 2017a), ecological continuity (Tibell 1992, Selva 2003, Goward and Arsenault 2018, Bell-Doyon et al. 2021, Wiersma and McMullin 2022) and biodiversity conservation value (Goward 1994, Malíček et al. 2019, Miller et al. 2020). Lichens are also numerous; in Canada and the United States alone, there are 5823 species in 805 genera, including allied fungi (Esslinger 2021). In British Columbia, there is a wide range of ecosystems and, as a result, it is a particularly rich region for lichens (Noble et al. 1987, Goward et al. 1994, Goward 1999, McCune and Geiser 2009, McCune 2017a, McCune 2017b). In coastal British Columbia, detailed studies have been completed for Haida Gwaii (Brodo 1993, Brodo 1995, Brodo and Ahti 1996, Brodo and Santesson 1997, Brodo and Wirth 1998, Brodo and Sloan 2005) and the southern part of Vancouver Island and the Gulf Islands (Noble 1982). The Central Coast, however, has been less studied and few lichen collections were previously made on Calvert Island.

Our goal was to create a first checklist of the lichens and allied fungi for Calvert Island. Our aims were to: **1.** Survey as many different locations and habitats as possible within the time frame of the 2018 Hakai Terrestrial BioBlitz; **2.** Collect voucher specimens of all species encountered and deposit them in publicly accessible herbaria; **3.** Generate internal transcribed spacer (ITS) sequences of all species collected to aid in identifications; and **4.** Highlight species that are rare or that represent major range extensions. Our results help to fill in distribution gaps for many species along the Central Coast of British Columbia and they provide a baseline species list for Calvert Island, from which new discoveries and future changes in populations can be acknowledged.

## Materials and methods

### Study site

**Figure 1.**
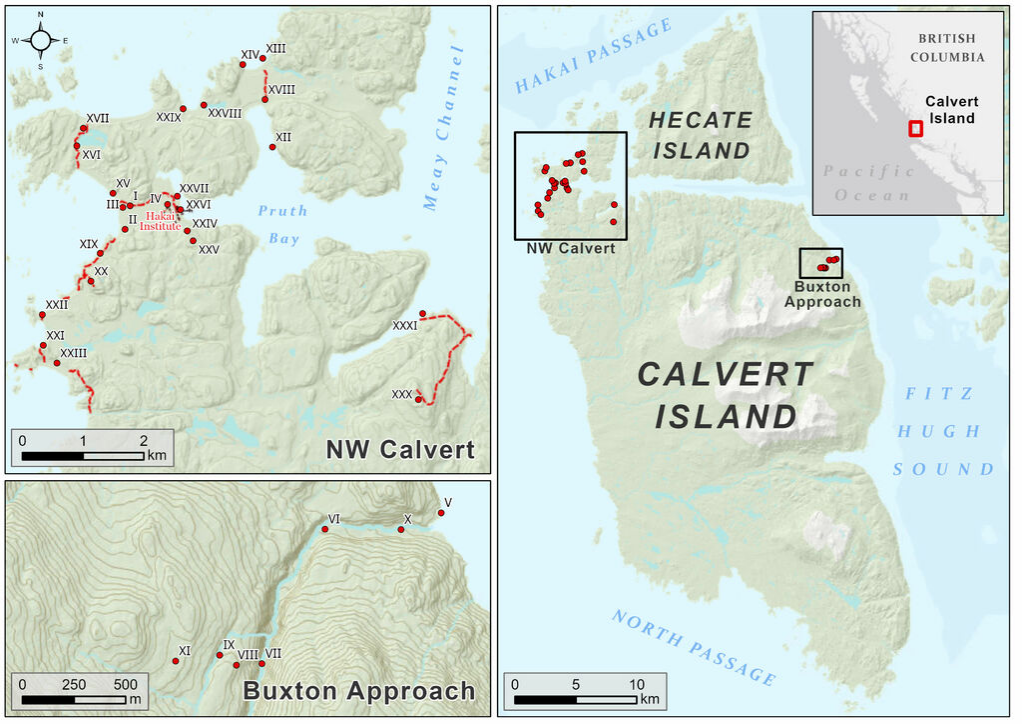
Location of the sites surveyed for lichens and allied fungi on Calvert Island during the 2018 Hakai Terrestrial BioBlitz. Roman numerals correspond with those in the Table 1, where the habitat descriptions and coordinates are provided.

The study site is located on northern Calvert Island (51.6592, -128.1365), on the Central Coast of British Columbia (also known as the Great Bear Rainforest) on the west coast of Canada (Fig. 1). Calvert Island is one of the larger outer Central Coast islands and is exposed to the northeast Pacific. Its shorelines are dominated by rocky, convoluted cliffs on both the exposed western side and protected eastern side of the island. Several large sandy beaches are on the south, west and north aspects of the island. Archaeological evidence and Haíłzaqv oral histories suggest that humans have occupied these landscapes for at least 13,000 years (McLaren et al. 2018). Historical human occupation has had an effect on fire regimes (Hoffman et al. 2016, Hoffman et al. 2017) and continues to affect forest productivity and composition near known habitation sites (Trant et al. 2016, Fisher et al. 2019).

Northern Calvert Island is located within the very wet hypermaritime subzone of the Coastal Western Hemlock biogeoclimatic classification (CWHvh2; Meidinger and Pojar (1991)), which is characterised by cool summers (mean warmest month 13.9ºC), warm winters (mean coldest month 3.0ºC) and large amounts of precipitation (mean annual precipitation 2951 mm) (Meidinger and Pojar 1991). Rain is dominant and snowpack only persists at higher elevations (> 1000 m; Oliver et al. (2017)). The geological substrate of all the sampling sites is granodiorite bedrock (Roddick 1996). Soils overlying these substrates are usually < 1 m thick and have formed in sandy colluvium and patchy morainal deposits. Chemical weathering and the accumulation of organic matter created nutrient-poor Podzol and Folisol soils (International Union of Soil Sciences 2015). Excess soil water regulates ecosystems on Calvert Island and high precipitation, abundant fog and low evapotranspiration causes anaerobic conditions in flat or low-lying areas (Banner et al. 2005). These conditions promote moss growth and reduce the decomposition of organic matter resulting in an abundance of wet soils, wetland ecosystems and forests. However, subtle variations in slope or drainage can result in significant differences to forest productivity and dense stands of productive rainforests occur in small pockets along coastal margins and river valley bottoms (Banner et al. 2005, Thompson et al. 2016). Elevations of the study sites ranged from 0 to 125 m above sea level. Detailed information on the dominant vegetation of collection sites is in Table 1.

Calvert Island is located on a sea-level hinge point that has experienced very modest sea level changes for more than 10,000 years (McLaren et al. 2014) which partly explains the long history of human occupation (McLaren et al. 2015), with corresponding impacts to the environment (Trant et al. 2016, Cox et al. 2019, Fisher et al. 2019). Calvert Island is remote and only accessible by boat or seaplane and the Hakai Institute’s field station is located on Pruth Bay to the northwest of the Island (Fig. 1). There are no roads or vehicles on Calvert Island and it has never been logged or mined. A network of trails maintained by BC Parks originating at the Hakai Institute provide foot accessibility to the northwest and north regions of the Island.

### Sampling and identification

Our survey of the lichens and allied fungi of Calvert Island occurred during the 2018 Hakai Terrestrial BioBlitz on 15-22 June. We surveyed 31 sites (Fig. 1, Table 1) following a floristic habitat sampling methodology (Newmaster et al. 2005), also referred to as an ‘intelligent meander’ (Selva 2003), because it provides the freedom to explore broadly and capture overall cryptogam diversity more effectively than representative plots. We surveyed as many ecosystems and mesohabitats (e.g. streams, rock outcrops) as possible in the limited timeframe of the bioblitz. We also attempted to assess as many microhabitats (e.g. snags and different tree species and rock types) as possible.

We examined lichen morphology and chemical spot tests using stereo- and compound microscopes. Chemical spot tests were performed with paraphenylenediamine in ethyl alcohol, 50% nitric acid, sodium hypochlorite, 10–20% potassium hydroxide and Lugol’s iodine (Brodo et al. 2001). We further examined chemistry using a longwave ultraviolet light chamber (365 nm) and thin-layer chromatography (TLC) in solvent systems A, B′ and C (Culberson and Kristinsson 1970, Orange et al. 2010). We captured images with a Leica DVM6 digital microscope. Maps were produced in ArcMap 10.5.1 (ESRI 2011). We deposited specimens at the Canadian Museum of Nature (CANL) and the Beaty Biodiversity Museum, University of British Columbia (UBC).

### Molecular analysis

We subsampled dry lichen tissue (2–5 mm) from each sample using a stereomicroscope and sterilised fine-tipped tweezers while ensuring that there were no vegetative propagules (i.e. soredia or isidia) from other lichens and no lichenicolous fungi. The Canadian Centre for DNA Barcoding (CCDB) then conducted DNA extractions following in‑house protocols (Ivanova et al. 2008, Ivanova et al. 2011). They ground lichen fragments into powder using stainless steel beads in a TissueLyser (Qiagen, USA) at 28 Hz for 60 seconds. The material then incubated in CTAB buffer at 65°C for 1 hour before DNA was extracted using a semi-automated method employing glass fibre filtration (Ivanova et al. 2008, Fazekas et al. 2012). The final concentration of the eluted DNA was 20-40 ng/μl.

Fungal primers ITS-1F (Gardes and Bruns 1993) and ITS4 (White et al. 1990) were used for amplification of ITS1, 5.8S and ITS2 regions. PCR reactions had a total volume of 12.5 μl and included: 6.25 μl of 10% trehalose, 2.00 μl of ultrapure water, 1.25 μl 10× PCR Platinum Taq buffer [500 mM KCl, 200 mM Tris–HCl (pH 8.4)], 0.625 μl MgCl_2_ (50 mM) (Invitrogen, Thermo Fisher Scientific), 0.125 μl of each primer (0.01 mM), 0.0625 μl of dNTP (10 mM), 0.3 U of Platinum DNA Polymerase (5 U/μl) (Invitrogen, Thermo Fisher Scientific) and 2.0 μl of DNA template (Kuzmina and Ivanova 2011). The thermocycle program consisted of 94°C for 2 min, 40 cycles of 94°C for 30 s, 50°C for 30 s and 72°C for 1 min, with a final extension at 72°C for 5 min. PCR products were visualised on a 2% agarose gel using an E-Gel96® Pre-cast Agarose Electrophoresis System (Invitrogen, Thermo Fisher Scientific). Bidirectional sequencing using ITS-1F and ITS4 primers was done using the BigDye® Terminator v.3.1 Cycle Sequencing Kit (Applied Biosystems, Thermo Fisher Scientific) on an ABI 3730xl Genetic Analyzer (Applied Biosystems, Thermo Fisher Scientific) following Ivanova and Grainger (2007). Bidirectional sequences were assembled in CodonCode 8.0.2 and manually edited.

In GenBank (Benson et al. 2013), we used the Standard Nucleotide Basic Local Alignment Search Tool (BLASTn) to compare the similarity of our sequences to public libraries. The results helped to confirm the identity of our specimens. We deposited all sequences in the Barcode of Life Database (BOLD; Ratnasingham and Hebert (2007)) and GenBank. Accession numbers are provided for both databases in the annotated species list.

### List assembling

The annotated species list is arranged alphabetically by genus and species. Species authors are generally abbreviated following Brummitt and Powell (1996) or the 24^th^ edition of the North American Lichen Checklist (Esslinger 2021). Any deviance from Esslinger’s list represents the opinion of the authors. Specimens collected by McMullin are deposited at CANL and those collected by Schofield and Simon are deposited at UBC. Roman numerals correspond to collection sites (see Fig. 1 and Table 1). Numbers following ‘BOLD’ and ‘GenBank’ are database accession numbers.

## Checklists

### Annotated checklist

#### Abrothallus parmeliarum

(Sommerf.) Arnold

0C7D490C-1910-53B6-86EA-2ADF17BB6142

##### Materials

- **Type status:** Other material. **Occurrence:** recordedBy: R.T. McMullin; occurrenceID: 312DD3A9-F8FA-5A32-96CE-AF2A19BF17B6; **Location:** locationID: XVI; decimalLatitude: 51.66051; decimalLongitude: -128.14587; **Event:** habitat: Lichenicolous on *Parmeliasquarrosa*; **Record Level:** institutionID: CANL; collectionID: McMullin 19665

##### Parasite of

*Parmeliasquarrosa*

#### Alectoria imshaugii

Brodo & D. Hawksw.

A6AA5DED-2D3B-5B79-9D28-9D41490C4CFB

##### Materials

- **Type status:** Other material. **Occurrence:** catalogNumber: BOLD CALV191-20; recordedBy: R.T. McMullin; otherCatalogNumbers: GenBank OQ843339; occurrenceID: 5A5402E4-C4C5-5092-935B-25D60B23B777; **Location:** locationID: XVI; decimalLatitude: 51.66051; decimalLongitude: -128.14587; **Event:** habitat: Lignicolous on a conifer snag; **Record Level:** institutionID: CANL; collectionID: McMullin 19665
- **Type status:** Other material. **Occurrence:** catalogNumber: BOLD CALV192-20; recordedBy: R.T. McMullin; otherCatalogNumbers: GenBank OQ843207; occurrenceID: 333B15C4-4E2B-5C99-AECC-81CA195E8B15; **Location:** locationID: XXIV; decimalLatitude: 51.65271; decimalLongitude: -128.12951; **Event:** habitat: Corticolous on *Piceasitchensis*; **Record Level:** institutionID: CANL; collectionID: McMullin 19755
- **Type status:** Other material. **Occurrence:** catalogNumber: BOLD CALV193-20; recordedBy: R.T. McMullin; otherCatalogNumbers: GenBank OQ843240; occurrenceID: 4DF4A0EC-470B-5D2C-8F9A-441A228A8ABC; **Location:** locationID: XVIII; decimalLatitude: 51.66476; decimalLongitude: -128.11798; **Event:** habitat: Corticolous on *Piceasitchensis*; **Record Level:** institutionID: CANL; collectionID: McMullin 19786
- **Type status:** Other material. **Occurrence:** recordedBy: R.T. McMullin; occurrenceID: EAF9A0BE-65C7-5477-81E0-A14C2C442789; **Location:** locationID: XIX; decimalLatitude: 51.65065; decimalLongitude: -128.14241; **Event:** habitat: Corticolous on *Thujaplicata*; **Record Level:** institutionID: CANL; collectionID: McMullin 19787
- **Type status:** Other material. **Occurrence:** recordedBy: R.T. McMullin; occurrenceID: 06BDB2BA-9B03-5FFB-85A1-7C2162D56BC7; **Location:** locationID: XXII; decimalLatitude: 51.64502; decimalLongitude: -128.15099; **Event:** habitat: Corticolous; **Record Level:** institutionID: CANL; collectionID: McMullin 19860

##### Notes

The discovery of additional coastal specimens of this typically interior species confirms its broader distribution first suggested by a single report from Haida Gwaii by Brodo and Hawksworth (1977). On Calvert Island, *A.imshaugii* is abundant.

#### Alectoria sarmentosa

(Ach.) Ach.

75A239BB-174E-5227-B090-2999269C72BE

##### Materials

- **Type status:** Other material. **Occurrence:** catalogNumber: BOLD CALV194-20; recordedBy: R.T. McMullin; otherCatalogNumbers: GenBank OQ843215; occurrenceID: 56AAA1C4-6387-5737-9093-CA9773F72C75; **Location:** locationID: IV; decimalLatitude: 51.65514; decimalLongitude: -128.13243; **Event:** habitat: Corticolous on *Tsuga*; **Record Level:** institutionID: CANL; collectionID: McMullin 19524
- **Type status:** Other material. **Occurrence:** recordedBy: R.T. McMullin; occurrenceID: A9A4EBFC-C152-5CBB-A2ED-8C1E4110FFC3; **Location:** locationID: XXVII; decimalLatitude: 51.65589; decimalLongitude: -128.13098; **Event:** habitat: Corticolous on *Alnusrubra*; **Record Level:** institutionID: CANL; collectionID: McMullin 19775
- **Type status:** Other material. **Occurrence:** catalogNumber: BOLD CALV196-20; recordedBy: R.T. McMullin; otherCatalogNumbers: GenBank OQ843292; occurrenceID: B75EFA3A-8DA5-55BB-AE96-D649118D998C; **Location:** locationID: XIX; decimalLatitude: 51.65065; decimalLongitude: -128.14241; **Event:** habitat: Corticolous on *Thujaplicata*; **Record Level:** institutionID: CANL; collectionID: McMullin 19790
- **Type status:** Other material. **Occurrence:** recordedBy: R.T. McMullin; occurrenceID: FE0CB4EF-8B81-5E9D-B162-442B83E4D672; **Location:** locationID: XX; decimalLatitude: 51.64809; decimalLongitude: -128.14378; **Identification:** identificationRemarks: TLC: alectoronic and usnic acids; **Event:** habitat: Corticolous on Pinuscontortassp.contorta; **Record Level:** institutionID: CANL; collectionID: McMullin 19842
- **Type status:** Other material. **Occurrence:** catalogNumber: BOLD PHAK289-20; recordedBy: A. Simon; otherCatalogNumbers: GenBank OQ922978; occurrenceID: C8205612-71E8-5ADD-93B2-59E8835B3879; **Location:** locationID: I; decimalLatitude: 51.65501; decimalLongitude: -128.13799; **Record Level:** institutionID: UBC; collectionID: Simon 749

#### Anisomeridium biforme

(Borrer) R.C. Harris

1AF8EC58-B7C8-5810-9D59-CFC7E5B01FF8

##### Materials

- **Type status:** Other material. **Occurrence:** recordedBy: R.T. McMullin; occurrenceID: 0F417F53-506B-5526-BFA7-D3309C4E5416; **Location:** locationID: XII; decimalLatitude: 51.66040; decimalLongitude: -128.11688; **Event:** habitat: Lignicolous on a snag; **Record Level:** institutionID: CANL; collectionID: McMullin 19603

#### Arthonia arthonioides

(Ach.) A.L. Sm.

D7145A23-9499-5E61-896D-188E282169EC

##### Materials

- **Type status:** Other material. **Occurrence:** recordedBy: R.T. McMullin; occurrenceID: 22194258-09CF-5739-888D-27BC9A293166; **Location:** locationID: XVI; decimalLatitude: 51.66051; decimalLongitude: -128.14587; **Event:** habitat: Corticolous on *Thujaplicata*; **Record Level:** institutionID: CANL; collectionID: McMullin 19872
- **Type status:** Other material. **Occurrence:** recordedBy: R.T. McMullin; occurrenceID: 626E13FB-0611-5D8B-BAC0-9D3784E7D9DA; **Location:** locationID: XVI; decimalLatitude: 51.66051; decimalLongitude: -128.14587; **Event:** habitat: Lignicolous on a snag; **Record Level:** institutionID: CANL; collectionID: McMullin 19873

#### Arthonia atra

(Pers.) A. Schneid.

37721DAA-89C2-53F9-8911-5B832CA7D52B

##### Materials

- **Type status:** Other material. **Occurrence:** recordedBy: R.T. McMullin; occurrenceID: D5EF128A-233B-5070-AA32-96C0DDC16538; **Location:** locationID: XII; decimalLatitude: 51.66040; decimalLongitude: -128.11688; **Event:** habitat: Corticolous on *Thujaplicata*; **Record Level:** institutionID: CANL; collectionID: McMullin 19580

#### Arthonia ilicina

Taylor

9BFF875B-1BF4-5DB1-8D7D-62D350B4A0C0

##### Materials

- **Type status:** Other material. **Occurrence:** recordedBy: R.T. McMullin; occurrenceID: 684E4E27-6CD6-552A-816C-ADD1CE72EE0F; **Location:** locationID: III; decimalLatitude: 51.65486; decimalLongitude: -128.13907; **Event:** habitat: Corticolous on *Alnusrubra*; **Record Level:** institutionID: CANL; collectionID: McMullin 19528
- **Type status:** Other material. **Occurrence:** catalogNumber: BOLD CALV199-20; recordedBy: R.T. McMullin; otherCatalogNumbers: GenBank OQ843334; occurrenceID: 0D4890A7-A857-5515-8888-5C9A57CC2ECF; **Location:** locationID: XII; decimalLatitude: 51.66040; decimalLongitude: -128.11688; **Event:** habitat: Corticolous on *Alnusrubra*; **Record Level:** institutionID: CANL; collectionID: McMullin 19602
- **Type status:** Other material. **Occurrence:** recordedBy: R.T. McMullin; occurrenceID: 43BF09DB-6530-5F4F-A1EC-968D021C6FAB; **Location:** locationID: I; decimalLatitude: 51.65501; decimalLongitude: -128.13799; **Event:** habitat: Corticolous on *Alnusrubra*; **Record Level:** institutionID: CANL; collectionID: McMullin 19720
- **Type status:** Other material. **Occurrence:** recordedBy: A. Simon; occurrenceID: A38700B5-AC47-57BE-BCE1-90488A289057; **Location:** locationID: V; decimalLatitude: 51.62022; decimalLongitude: -127.93070; **Record Level:** institutionID: UBC; collectionID: Simon 817

#### Arthonia norvegica

(Coppins & Tønsberg) McCune

7F95BB03-5DED-5E76-957E-234E4DCD3473

##### Materials

- **Type status:** Other material. **Occurrence:** recordedBy: R.T. McMullin; occurrenceID: DFD9555F-AAF5-5C2A-BB7C-9B7FB68DF8DB; **Location:** locationID: IV; decimalLatitude: 51.65514; decimalLongitude: -128.13243; **Event:** habitat: Corticolous on *Alnusrubra*; **Record Level:** institutionID: CANL; collectionID: McMullin 19881

#### Biatoropsis usnearum

Räsänen

001960D0-A982-5B72-BDB7-A55DA3E14DC9

##### Materials

- **Type status:** Other material. **Occurrence:** recordedBy: R.T. McMullin; occurrenceID: 77FC78EC-8C42-50D4-B0EE-E5DD94184595; **Location:** locationID: III; decimalLatitude: 51.65486; decimalLongitude: -128.13907; **Event:** habitat: Lichenicolous on *Usneafragilescens* on *Alnusrubra*; **Record Level:** institutionID: CANL; collectionID: McMullin 19533

##### Parasite of

*Usneafragilescens*

##### Notes

Non-lichenised fungus.

#### Brodoa oroarctica

(Krog) Goward

76814727-3A26-5817-9FBB-DF68A7933CC5

##### Materials

- **Type status:** Other material. **Occurrence:** recordedBy: R.T. McMullin; occurrenceID: 6200AF09-C535-5985-B05E-D18A135B3151; **Location:** locationID: XII; decimalLatitude: 51.66040; decimalLongitude: -128.11688; **Event:** habitat: Saxicolous; **Record Level:** institutionID: CANL; collectionID: McMullin 19581

#### Bryocaulon pseudosatoanum

(Asah.) Kärnefelt

9C7EB8BE-DB42-5D25-8E61-109CA9768AA6

##### Materials

- **Type status:** Other material. **Occurrence:** recordedBy: R.T. McMullin; occurrenceID: 73A44E25-7CB0-5761-BED4-C9A3CACCBEBE; **Location:** locationID: XIV; decimalLatitude: 51.66797; decimalLongitude: -128.12128; **Event:** habitat: Corticolous on a conifer; **Record Level:** institutionID: CANL; collectionID: McMullin 19600

#### Bryoria alaskana

Myllys & Goward

1598157C-5607-55F0-8923-A918BFC53117

##### Materials

- **Type status:** Other material. **Occurrence:** catalogNumber: BOLD CALV203-20; recordedBy: R.T. McMullin; otherCatalogNumbers: GenBank OQ843218; occurrenceID: CE0C373D-EDDA-50E3-B631-FE79A30167F0; **Location:** locationID: XXVII; decimalLatitude: 51.65589; decimalLongitude: -128.13098; **Event:** habitat: Corticolous on *Piceasitchensis*; **Record Level:** institutionID: CANL; collectionID: McMullin 19773

#### Bryoria americana

(Motyka) Holien

5D52C83B-04D5-5E3F-9D4A-92B523889439

##### Materials

- **Type status:** Other material. **Occurrence:** catalogNumber: BOLD CALV204-20; recordedBy: R.T. McMullin; otherCatalogNumbers: GenBank OQ843291; occurrenceID: DBE9AA60-DBDF-5F09-B717-1DC999B30A16; **Location:** locationID: XVIII; decimalLatitude: 51.66476; decimalLongitude: -128.11798; **Event:** habitat: Lignicolous; **Record Level:** institutionID: CANL; collectionID: McMullin 19551
- **Type status:** Other material. **Occurrence:** catalogNumber: BOLD CALV205-20; recordedBy: R.T. McMullin; otherCatalogNumbers: GenBank OQ843241; occurrenceID: 6B5D35DC-9759-54DF-94A7-F2B077C11206; **Location:** locationID: XVI; decimalLatitude: 51.66051; decimalLongitude: -128.14587; **Event:** habitat: Lignicolous on a conifer snag; **Record Level:** institutionID: CANL; collectionID: McMullin 19664
- **Type status:** Other material. **Occurrence:** catalogNumber: BOLD CALV206-20; recordedBy: R.T. McMullin; otherCatalogNumbers: GenBank OQ843326; occurrenceID: 826498A2-BD07-52E7-9F9B-3B3614E05D37; **Location:** locationID: XVIII; decimalLatitude: 51.66476; decimalLongitude: -128.11798; **Event:** habitat: Corticolous on *Thujaplicata*; **Record Level:** institutionID: CANL; collectionID: McMullin 19798
- **Type status:** Other material. **Occurrence:** recordedBy: R.T. McMullin; occurrenceID: 4A66AE0E-3B06-5388-9AF5-046282207C98; **Location:** locationID: XII; decimalLatitude: 51.66040; decimalLongitude: -128.11688; **Event:** habitat: Corticolous on *Tsuga*; **Record Level:** institutionID: CANL; collectionID: McMullin 19857
- **Type status:** Other material. **Occurrence:** recordedBy: R.T. McMullin; occurrenceID: E2D97217-D669-5B97-8B09-7BAA30856115; **Location:** locationID: XII; decimalLatitude: 51.66040; decimalLongitude: -128.11688; **Event:** habitat: Corticolous on *Tsuga*; **Record Level:** institutionID: CANL; collectionID: McMullin 19875

#### Bryoria bicolor

(Ehrh.) Brodo & D. Hawksw.

515D7FC0-C0EB-5919-95EE-5901BF91DBD4

##### Materials

**Figure 2.**
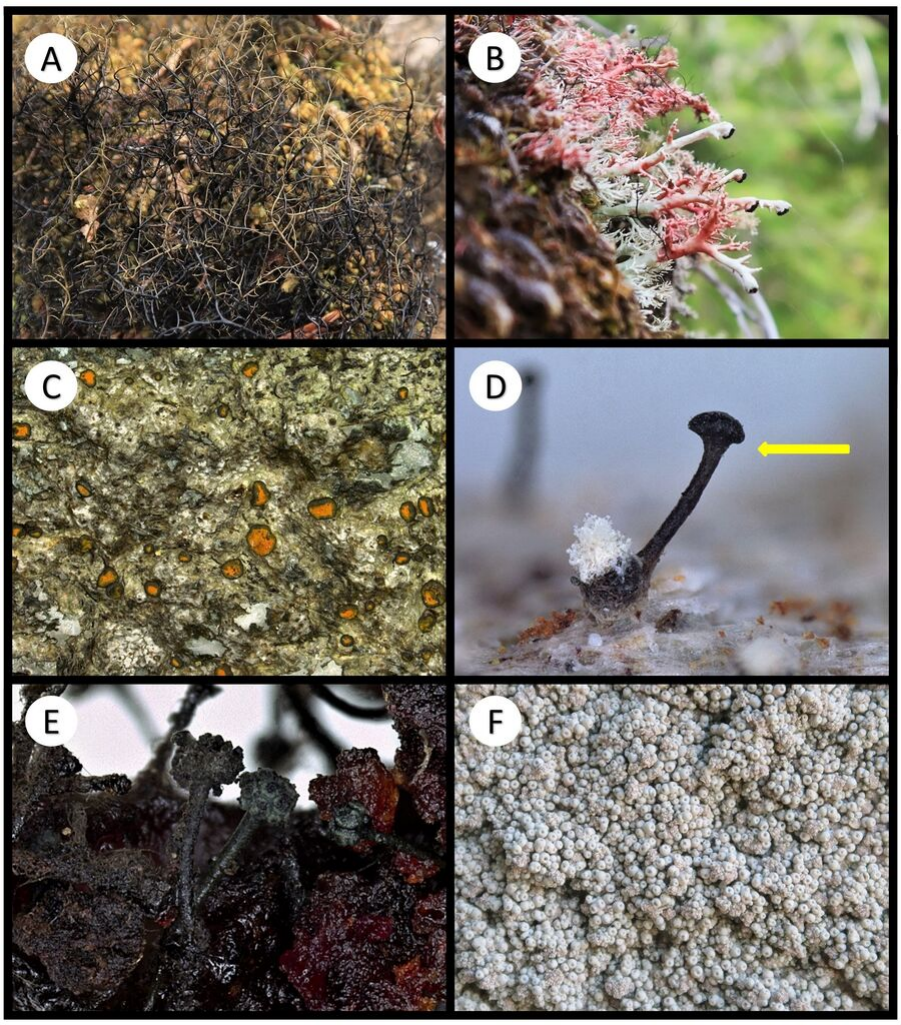
Lichens of Calvert Island. **A.** *Bryoriabicolor*, *McMullin 19567* (CANL). **B.** *Bunodophoronmelanocarpum*, *McMullin 19865* (CANL). **C.** *Caloplacalitoricola*, *McMullin 19883* (CANL). **D.** *Chaenothecopsislecanactidis* on a pycnidium of *Lecanactisabietina*, *McMullin 19628* (CANL). **E.** *Chaenothecopsisnigripunctata* on *Thuja* resin, *McMullin 19712* (CANL). **F.** *Coccotremamaritimum*, *McMullin 19750* (CANL).

- **Type status:** Other material. **Occurrence:** catalogNumber: BOLD CALV207-20; recordedBy: R.T. McMullin; otherCatalogNumbers: GenBank OQ843275; occurrenceID: FF7E723C-683E-5FD2-A0AF-DFEA6D674D33; **Location:** locationID: XX; decimalLatitude: 51.64809; decimalLongitude: -128.14378; **Event:** habitat: Corticolous on Pinuscontortassp.contorta; **Record Level:** institutionID: CANL; collectionID: McMullin 19500
- **Type status:** Other material. **Occurrence:** catalogNumber: BOLD CALV208-20; recordedBy: R.T. McMullin; otherCatalogNumbers: GenBank OQ843300; occurrenceID: 9F784AD3-7370-53AF-9EFD-B3F2506D8B77; **Location:** locationID: XIV; decimalLatitude: 51.66797; decimalLongitude: -128.12128; **Identification:** identificationRemarks: Figure 2A; **Event:** habitat: Terricolous; **Record Level:** institutionID: CANL; collectionID: McMullin 19567

#### Bryoria furcellata

(Fr.) Brodo & D. Hawksw.

33A6D56C-E702-5D2D-BCF8-6054033B6E28

##### Materials

- **Type status:** Other material. **Occurrence:** recordedBy: R.T. McMullin; occurrenceID: E27AF3E0-DF16-5B73-908D-90E7EF374D9D; **Location:** locationID: XX; decimalLatitude: 51.64809; decimalLongitude: -128.14378; **Event:** habitat: Corticolous on Pinuscontortassp.contorta; **Record Level:** institutionID: CANL; collectionID: McMullin 19507

#### Bryoria fuscescens

(Gyeln.) Brodo & D. Hawksw.

D51ECD11-FEC3-512D-BD29-9C5C9E2E8DC2

##### Materials

- **Type status:** Other material. **Occurrence:** recordedBy: R.T. McMullin; occurrenceID: FF009412-F0B6-58AA-842A-E531F1CD1F42; **Location:** locationID: XVI; decimalLatitude: 51.66051; decimalLongitude: -128.14587; **Event:** habitat: Lignicolous on a snag; **Record Level:** institutionID: CANL; collectionID: McMullin 19669
- **Type status:** Other material. **Occurrence:** recordedBy: R.T. McMullin; occurrenceID: D18558E8-9E69-58E8-8C0F-9C5BA9CB7BD3; **Location:** locationID: XXII; decimalLatitude: 51.64502; decimalLongitude: -128.15099; **Event:** habitat: Corticolous on *Thujaplicata*; **Record Level:** institutionID: CANL; collectionID: McMullin 19721
- **Type status:** Other material. **Occurrence:** recordedBy: R.T. McMullin; occurrenceID: 9D3CE791-E1FE-5A7C-A379-318C372ACD22; **Location:** locationID: XIX; decimalLatitude: 51.65065; decimalLongitude: -128.14241; **Event:** habitat: Corticolous on *Thujaplicata*; **Record Level:** institutionID: CANL; collectionID: McMullin 19792
- **Type status:** Other material. **Occurrence:** recordedBy: R.T. McMullin; occurrenceID: 7B41306F-984A-501E-9DC1-D13DE63F5DE5; **Location:** locationID: XII; decimalLatitude: 51.66040; decimalLongitude: -128.11688; **Event:** habitat: Corticolous on *Tsuga*; **Record Level:** institutionID: CANL; collectionID: McMullin 19849

#### Bryoria glabra

(Motyka) Brodo & D. Hawksw.

84F11215-3C92-5688-B192-B3BA39B66585

##### Materials

- **Type status:** Other material. **Occurrence:** catalogNumber: BOLD CALV209-20; recordedBy: R.T. McMullin; otherCatalogNumbers: GenBank OQ843331; occurrenceID: C53370AA-79D4-5154-A7B8-00C00F406007; **Location:** locationID: V; decimalLatitude: 51.62022; decimalLongitude: -127.93070; **Event:** habitat: Corticolous on *Piceasitchensis*; **Record Level:** institutionID: CANL; collectionID: McMullin 19592
- **Type status:** Other material. **Occurrence:** catalogNumber: BOLD CALV210-20; recordedBy: R.T. McMullin; otherCatalogNumbers: GenBank OQ843314; occurrenceID: 69ADFA09-1114-5BD0-978B-A1C56AE808D7; **Location:** locationID: XIV; decimalLatitude: 51.66797; decimalLongitude: -128.12128; **Event:** habitat: Corticolous on a conifer; **Record Level:** institutionID: CANL; collectionID: McMullin 19666
- **Type status:** Other material. **Occurrence:** catalogNumber: BOLD CALV212-20; recordedBy: R.T. McMullin; otherCatalogNumbers: GenBank OQ843266; occurrenceID: 34EC512E-B668-565A-87D5-AA187CE400AF; **Location:** locationID: XVI; decimalLatitude: 51.66051; decimalLongitude: -128.14587; **Event:** habitat: Lignicolous on a conifer snag; **Record Level:** institutionID: CANL; collectionID: McMullin 19877

#### Bryoria irwinii

Myllys & Goward

607258EB-6EB1-5665-B816-46B0CBD808FD

##### Materials

- **Type status:** Other material. **Occurrence:** catalogNumber: BOLD CALV213-20; recordedBy: R.T. McMullin; otherCatalogNumbers: GenBank OQ843278; occurrenceID: DB1F9106-2CC7-5E3E-A163-7CAAE769394A; **Location:** locationID: XVI; decimalLatitude: 51.66051; decimalLongitude: -128.14587; **Event:** habitat: Lignicolous on a snag; **Record Level:** institutionID: CANL; collectionID: McMullin 19574

#### Bryoria kockiana

Velmala, Myllys & Goward

62A346F4-B205-557B-88C5-9AC767AB6709

##### Materials

- **Type status:** Other material. **Occurrence:** recordedBy: R.T. McMullin; occurrenceID: 3140FB0A-2CEF-5DCF-B46B-3468F2F862FC; **Location:** locationID: XII; decimalLatitude: 51.66040; decimalLongitude: -128.11688; **Event:** habitat: Corticolous on *Tsuga*; **Record Level:** institutionID: CANL; collectionID: McMullin 19593

#### Bryoria pseudofuscescens var. pikei

(Brodo & D. Hawksw.) McCune

AEB75FDD-391C-53FA-A945-B96A16F96E9F

##### Materials

- **Type status:** Other material. **Occurrence:** catalogNumber: BOLD CALV215-20; recordedBy: R.T. McMullin; otherCatalogNumbers: GenBank OQ843367; occurrenceID: 9C18B499-9E6A-5152-A164-31589A1CB2FF; **Location:** locationID: XXVII; decimalLatitude: 51.65589; decimalLongitude: -128.13098; **Event:** habitat: Corticolous on *Piceasitchensis*; **Record Level:** institutionID: CANL; collectionID: McMullin 19781
- **Type status:** Other material. **Occurrence:** recordedBy: R.T. McMullin; occurrenceID: 625E3880-50FF-5B2C-8F6C-0DE0673E14B8; **Location:** locationID: XII; decimalLatitude: 51.66040; decimalLongitude: -128.11688; **Event:** habitat: Corticolous on *Tsuga*; **Record Level:** institutionID: CANL; collectionID: McMullin 19847
- **Type status:** Other material. **Occurrence:** recordedBy: A. Simon; occurrenceID: 991B8ECA-2317-5A3B-8040-77BE7016AF0B; **Location:** locationID: XII; decimalLatitude: 51.66040; decimalLongitude: -128.11688; **Record Level:** institutionID: UBC; collectionID: Simon 783

#### Bryoria trichodes trichodes

(Michx.) Brodo & D. Hawksw.

C11A2768-AE13-573B-B9ED-6989347E4A36

##### Materials

- **Type status:** Other material. **Occurrence:** recordedBy: R.T. McMullin; occurrenceID: CDBFA30B-0F79-558E-81CB-DAFF401BABAF; **Location:** locationID: V; decimalLatitude: 51.62022; decimalLongitude: -127.93070; **Event:** habitat: Corticolous on *Thujaplicata*; **Record Level:** institutionID: CANL; collectionID: McMullin 19616

#### Buellia muriformis

A. Nordin & Tønsberg

DC853F23-890E-5088-B8E4-12C2EA0F78F1

##### Materials

- **Type status:** Other material. **Occurrence:** catalogNumber: BOLD CALV217-20; recordedBy: R.T. McMullin; otherCatalogNumbers: GenBank OQ843358; occurrenceID: D55E99AE-6407-564B-900A-BB48E9D05AF2; **Location:** locationID: XIX; decimalLatitude: 51.65065; decimalLongitude: -128.14241; **Event:** habitat: Corticolous on a snag; **Record Level:** institutionID: CANL; collectionID: McMullin 19735

#### Bunodophoron melanocarpum

(Sw.) Wedin

7EAAA94A-FF83-5215-BEED-E895AE391397

##### Materials

- **Type status:** Other material. **Occurrence:** recordedBy: R.T. McMullin; occurrenceID: 7C241AD0-883B-5188-803B-D924A336C5D3; **Location:** locationID: XVI; decimalLatitude: 51.66051; decimalLongitude: -128.14587; **Event:** habitat: Corticolous on the base of a conifer; **Record Level:** institutionID: CANL; collectionID: McMullin 19562
- **Type status:** Other material. **Occurrence:** recordedBy: R.T. McMullin; occurrenceID: 46557DE8-0111-55EE-A35C-D223F9A95973; **Location:** locationID: XXIV; decimalLatitude: 51.65271; decimalLongitude: -128.12951; **Identification:** identificationRemarks: Figure 2B; **Event:** habitat: Corticolous on the base of *Thujaplicata*; **Record Level:** institutionID: CANL; collectionID: McMullin 19865
- **Type status:** Other material. **Occurrence:** catalogNumber: BOLD CALV223-20; recordedBy: R.T. McMullin; otherCatalogNumbers: GenBank OQ843366; occurrenceID: 47330294-E573-56B1-ADBE-3355BA578BB6; **Location:** locationID: XIX; decimalLatitude: 51.65065; decimalLongitude: -128.14241; **Event:** habitat: Corticolous on a conifer snag; **Record Level:** institutionID: CANL; collectionID: McMullin 19866
- **Type status:** Other material. **Occurrence:** catalogNumber: BOLD PHAK324-20; recordedBy: A. Simon; otherCatalogNumbers: GenBank OQ922933; occurrenceID: 7C75BFAE-31F8-54B9-AAEB-16F9D462357E; **Location:** locationID: XIX; decimalLatitude: 51.65065; decimalLongitude: -128.14241; **Record Level:** institutionID: UBC; collectionID: Simon 815

#### Calicium lenticulare

Ach.

53A92AE6-A806-5DA8-8D82-CA6655FBC70A

##### Materials

- **Type status:** Other material. **Occurrence:** recordedBy: R.T. McMullin; occurrenceID: 1E6BE4F4-5FCF-51E9-9FEC-FC9B826F7C02; **Location:** locationID: VIII; decimalLatitude: 51.61611; decimalLongitude: -127.93962; **Event:** habitat: Lignicolous; **Record Level:** institutionID: CANL; collectionID: McMullin 19611
- **Type status:** Other material. **Occurrence:** recordedBy: R.T. McMullin; occurrenceID: BEA3A5A8-9A56-573C-B8BD-92E23D24735A; **Location:** locationID: XVI; decimalLatitude: 51.66051; decimalLongitude: -128.14587; **Event:** habitat: Lignicolous on a snag; **Record Level:** institutionID: CANL; collectionID: McMullin 19621
- **Type status:** Other material. **Occurrence:** recordedBy: R.T. McMullin; occurrenceID: D4F4F6D4-1D45-52AA-8A47-4A632FACF095; **Location:** locationID: VIII; decimalLatitude: 51.61611; decimalLongitude: -127.93962; **Event:** habitat: Lignicolous on a snag; **Record Level:** institutionID: CANL; collectionID: McMullin 19638
- **Type status:** Other material. **Occurrence:** recordedBy: R.T. McMullin; occurrenceID: 50E85110-6827-58B3-8D4D-C1AA61DF1D21; **Location:** locationID: VIII; decimalLatitude: 51.61611; decimalLongitude: -127.93962; **Event:** habitat: Corticolous on *Thujaplicata*; **Record Level:** institutionID: CANL; collectionID: McMullin 19697
- **Type status:** Other material. **Occurrence:** catalogNumber: BOLD CALV220-20; recordedBy: R.T. McMullin; otherCatalogNumbers: GenBank OQ843318; occurrenceID: FBE283CF-E1B8-55B6-9503-45CCF8E4C89D; **Location:** locationID: XIX; decimalLatitude: 51.65065; decimalLongitude: -128.14241; **Event:** habitat: Lignicolous on *Thujaplicata*; **Record Level:** institutionID: CANL; collectionID: McMullin 19737
- **Type status:** Other material. **Occurrence:** recordedBy: R.T. McMullin; occurrenceID: 47A04A02-9281-52E7-A614-7776E6DF25EA; **Location:** locationID: XIX; decimalLatitude: 51.65065; decimalLongitude: -128.14241; **Event:** habitat: Lignicolous on *Thujaplicata*; **Record Level:** institutionID: CANL; collectionID: McMullin 19744
- **Type status:** Other material. **Occurrence:** recordedBy: R.T. McMullin; occurrenceID: 55BD2150-D976-5D41-8B84-61192BE2ABCA; **Location:** locationID: XIX; decimalLatitude: 51.65065; decimalLongitude: -128.14241; **Event:** habitat: Lignicolous on a stump; **Record Level:** institutionID: CANL; collectionID: McMullin 19748
- **Type status:** Other material. **Occurrence:** recordedBy: R.T. McMullin; occurrenceID: 8B05A843-460E-5CB6-9AEA-C06D6F275DEB; **Location:** locationID: XXIV; decimalLatitude: 51.65271; decimalLongitude: -128.12951; **Event:** habitat: Corticolous on a conifer; **Record Level:** institutionID: CANL; collectionID: McMullin 19862
- **Type status:** Other material. **Occurrence:** recordedBy: R.T. McMullin; occurrenceID: 85C56748-E490-52CF-BC33-80D1F724FA9B; **Location:** locationID: XXII; decimalLatitude: 51.64502; decimalLongitude: -128.15099; **Event:** habitat: Corticolous; **Record Level:** institutionID: CANL; collectionID: McMullin 19880

#### Caloplaca litoricola

Brodo

B0B28090-068B-5B71-8441-8BCF79876079

##### Materials

- **Type status:** Other material. **Occurrence:** recordedBy: R.T. McMullin; occurrenceID: 7E5C5138-C6EE-55FA-B494-820C087223FF; **Location:** locationID: V; decimalLatitude: 51.62022; decimalLongitude: -127.93070; **Identification:** identificationRemarks: Figure 2C; **Event:** habitat: Saxicolous; **Record Level:** institutionID: CANL; collectionID: McMullin 19883

#### Caloplaca obesimarginata

Søchting

B5D12661-9935-5B5D-9AF1-77F2976B195F

##### Materials

- **Type status:** Other material. **Occurrence:** recordedBy: R.T. McMullin; occurrenceID: 36176A40-8FB3-5E78-A593-A644CA7AF424; **Location:** locationID: II; decimalLatitude: 51.65285; decimalLongitude: -128.13873; **Identification:** identificationRemarks: TLC: methyl parietinate; **Event:** habitat: Corticolous on *Alnusrubra*; **Record Level:** institutionID: CANL; collectionID: McMullin 19736
- **Type status:** Other material. **Occurrence:** recordedBy: R.T. McMullin; occurrenceID: 1EAB3113-E9A7-5C8D-9DDB-23E47F6EF549; **Location:** locationID: XXII; decimalLatitude: 51.64502; decimalLongitude: -128.15099; **Identification:** identificationRemarks: TLC: methyl parietinate; **Event:** habitat: Corticolous on *Piceasitchensis*; **Record Level:** institutionID: CANL; collectionID: McMullin 19852

#### Caloplaca oregona

H. Magn.

CFF8DD41-AD93-541A-8783-87B9B50A0A6D

##### Materials

- **Type status:** Other material. **Occurrence:** recordedBy: A. Simon; occurrenceID: 618BF012-8749-5620-A30E-4242725DD7CB; **Location:** locationID: XXI; decimalLatitude: 51.64221; decimalLongitude: -128.15085; **Record Level:** institutionID: UBC; collectionID: Simon 839

#### Cetraria aculeata

(Schreb.) Fr.

EC2D1054-4605-5D71-86E3-62C9D81E6142

##### Materials

- **Type status:** Other material. **Occurrence:** catalogNumber: BOLD CALV222-20; recordedBy: R.T. McMullin; otherCatalogNumbers: GenBank OQ843302; occurrenceID: 5A393EA8-2051-5E4F-BFFF-800B7400431C; **Location:** locationID: XX; decimalLatitude: 51.64809; decimalLongitude: -128.14378; **Event:** habitat: Terricolous; **Record Level:** institutionID: CANL; collectionID: McMullin 19515

#### Chaenotheca brunneola

(Ach.) Müll. Arg.

88079A9E-E75D-5C12-B030-821275F9BD6F

##### Materials

- **Type status:** Other material. **Occurrence:** recordedBy: R.T. McMullin; occurrenceID: D93C64C1-7194-5E71-9DBA-4A9AA649EA75; **Location:** locationID: VIII; decimalLatitude: 51.61611; decimalLongitude: -127.93962; **Event:** habitat: Lignicolous on *Thujaplicata*; **Record Level:** institutionID: CANL; collectionID: McMullin 19612
- **Type status:** Other material. **Occurrence:** recordedBy: R.T. McMullin; occurrenceID: A5F622BA-84FD-53D9-8C51-7CC5F3F1C60D; **Location:** locationID: XVI; decimalLatitude: 51.66051; decimalLongitude: -128.14587; **Event:** habitat: Lignicolous; **Record Level:** institutionID: CANL; collectionID: McMullin 19619
- **Type status:** Other material. **Occurrence:** catalogNumber: BOLD CALV153-20; recordedBy: R.T. McMullin; otherCatalogNumbers: GenBank OQ843252; occurrenceID: 818BAAA7-972A-581D-A028-8E139E292F64; **Location:** locationID: XVI; decimalLatitude: 51.66051; decimalLongitude: -128.14587; **Event:** habitat: Lignicolous on snag; **Record Level:** institutionID: CANL; collectionID: McMullin 19635
- **Type status:** Other material. **Occurrence:** recordedBy: R.T. McMullin; occurrenceID: 526EDC51-2965-5260-A21E-D8D56B915046; **Location:** locationID: XXIV; decimalLatitude: 51.65271; decimalLongitude: -128.12951; **Event:** habitat: Lignicolous; **Record Level:** institutionID: CANL; collectionID: McMullin 19858
- **Type status:** Other material. **Occurrence:** recordedBy: R.T. McMullin; occurrenceID: 99BE8E96-1476-5375-B592-4B1804558450; **Location:** locationID: XVIII; decimalLatitude: 51.66476; decimalLongitude: -128.11798; **Event:** habitat: Lignicolous; **Record Level:** institutionID: CANL; collectionID: McMullin 19859

##### Symbiotic with

*Dictyochloropsis*

#### Chaenotheca furfuracea

(L.) Tibell

60906A81-1605-5AEF-9184-53FF9A765ECC

##### Materials

- **Type status:** Other material. **Occurrence:** recordedBy: R.T. McMullin; occurrenceID: CBA5D378-07C5-5916-9596-67B908399635; **Location:** locationID: X; decimalLatitude: 51.61977; decimalLongitude: -127.93245; **Event:** habitat: Terricolous; **Record Level:** institutionID: CANL; collectionID: McMullin 19620
- **Type status:** Other material. **Occurrence:** recordedBy: R.T. McMullin; occurrenceID: 711F45F3-B0D9-5C9E-BF8E-1830C8668AEA; **Location:** locationID: X; decimalLatitude: 51.61977; decimalLongitude: -127.93245; **Event:** habitat: Terricolous; **Record Level:** institutionID: CANL; collectionID: McMullin 19677

#### Chaenotheca stemonea

(Ach.) Müll. Arg.

8DF4604E-1FFD-53AF-B192-45F6E8BD4E2B

##### Materials

- **Type status:** Other material. **Occurrence:** recordedBy: R.T. McMullin; occurrenceID: E213CA41-5011-5189-8D9C-23782E11D08B; **Location:** locationID: XIX; decimalLatitude: 51.65065; decimalLongitude: -128.14241; **Event:** habitat: Corticolous on base of *Thujaplicata*; **Record Level:** institutionID: CANL; collectionID: McMullin 19537
- **Type status:** Other material. **Occurrence:** recordedBy: R.T. McMullin; occurrenceID: DB0376E4-800A-50EA-B08E-F6B4653B15B0; **Location:** locationID: X; decimalLatitude: 51.61977; decimalLongitude: -127.93245; **Event:** habitat: Corticolous on *Thujaplicata*; **Record Level:** institutionID: CANL; collectionID: McMullin 19864

##### Symbiotic with

*Stichococcus*

#### Chaenotheca trichialis

(Ach.) Th. Fr.

53286640-6A82-53D1-B73B-F2C72A10D179

##### Materials

- **Type status:** Other material. **Occurrence:** recordedBy: R.T. McMullin; occurrenceID: C2D4ACEF-DDBE-5816-BAA5-619B85FCCEBE; **Location:** locationID: XXIV; decimalLatitude: 51.65271; decimalLongitude: -128.12951; **Event:** habitat: Lignicolous; **Record Level:** institutionID: CANL; collectionID: McMullin 19863

##### Symbiotic with

*Stichococcus*

#### Chaenothecopsis debilis

(Turner & Borrer ex Sm.) Tibell

F349D034-32F6-577A-AA16-8012644849DF

##### Materials

- **Type status:** Other material. **Occurrence:** recordedBy: R.T. McMullin; occurrenceID: F7713568-3E01-5CF2-ACFF-4F62F5AA5505; **Location:** locationID: VIII; decimalLatitude: 51.61611; decimalLongitude: -127.93962; **Event:** habitat: Lignicolous; **Record Level:** institutionID: CANL; collectionID: McMullin 19591

##### Notes

Non-lichenised fungus.

#### Chaenothecopsis lecanactidis

Tibell

52621308-06B9-5D3B-ADF7-BC06B36422D1

##### Materials

- **Type status:** Other material. **Occurrence:** recordedBy: R.T. McMullin; occurrenceID: 8ABFFB63-1F56-5B8B-B5BA-1DB07AF0BAD9; **Location:** locationID: XVI; decimalLatitude: 51.66051; decimalLongitude: -128.14587; **Identification:** identificationRemarks: Figure 2D; **Event:** habitat: Lichenicolous on *Lecanactisabietina*; **Record Level:** institutionID: CANL; collectionID: McMullin 19628

##### Parasite of

*Lecanactisabietina*

##### Notes

Non-lichenised fungus.

#### Chaenothecopsis nigripunctata

Rikkinen

21EA40FF-7957-515E-8C3A-7D45AE739AD6

##### Materials

- **Type status:** Other material. **Occurrence:** recordedBy: R.T. McMullin; occurrenceID: 581A3CDD-0D45-55E7-BB41-E3585E4803E2; **Location:** locationID: VIII; decimalLatitude: 51.61611; decimalLongitude: -127.93962; **Identification:** identificationRemarks: Figure 2E; **Event:** habitat: Resinicolous on *Tsuga*; **Record Level:** institutionID: CANL; collectionID: McMullin 19712

##### Notes

Non-lichenised fungus.

#### Chaenothecopsis pusilla

(Ach.) A.F.W. Schmidt

3AE283BC-F54D-5DD6-9DD5-ED549F45E85F

##### Materials

- **Type status:** Other material. **Occurrence:** recordedBy: R.T. McMullin; occurrenceID: 08C5280A-C777-5421-95DF-997020E108C3; **Location:** locationID: VII; decimalLatitude: 51.61615; decimalLongitude: -127.93851; **Event:** habitat: Lignicolous on *Thujaplicata*; **Record Level:** institutionID: CANL; collectionID: McMullin 19641
- **Type status:** Other material. **Occurrence:** recordedBy: R.T. McMullin; occurrenceID: FFACAF3A-0162-506C-9E8A-C4B67221331D; **Location:** locationID: VIII; decimalLatitude: 51.61611; decimalLongitude: -127.93962; **Event:** habitat: Lignicolous on a snag; **Record Level:** institutionID: CANL; collectionID: McMullin 19874

##### Notes

Non-lichenised fungus.

#### Chaenothecopsis sp.

B2B0D989-444F-5840-8E53-20DBCFE85095

##### Materials

- **Type status:** Other material. **Occurrence:** recordedBy: R.T. McMullin; occurrenceID: D87A8245-022D-527B-A518-B5FEB959B689; **Location:** locationID: XVI; decimalLatitude: 51.66051; decimalLongitude: -128.14587; **Event:** habitat: Lichenicolous on *Arthoniaarthonioides* on a snag; **Record Level:** institutionID: CANL; collectionID: McMullin 19559
- **Type status:** Other material. **Occurrence:** recordedBy: R.T. McMullin; occurrenceID: 94F47B7D-8AE6-52DA-9E81-4BDBD26BE2EA; **Location:** locationID: XVI; decimalLatitude: 51.66051; decimalLongitude: -128.14587; **Event:** habitat: Lichenicolous on *Arthoniaarthonioides* on Thujaplicata; **Record Level:** institutionID: CANL; collectionID: McMullin 19615
- **Type status:** Other material. **Occurrence:** recordedBy: R.T. McMullin; occurrenceID: 90505922-4E29-5474-8E98-14D0D8673EEA; **Location:** locationID: XIX; decimalLatitude: 51.65065; decimalLongitude: -128.14241; **Event:** habitat: Lichenicolous on *Arthoniaarthonioides*; **Record Level:** institutionID: CANL; collectionID: McMullin 19746

##### Parasite of

*Arthoniaarthonioides*

##### Notes

Non-lichenised fungus. Apothecia black, epruinose, 0.4–0.9 mm tall, capitulum lenticular to subspherical, excipulum well developed, hypothecium brown. Asci 32.5–37.5 μm long (n = 10), ascospores uniseriate. Ascospores medium brown, 2-celled, 6.0–9.0 x 2.5–3 μm. Stalk and excipulum K− and not swelling in K. Appears to be a novel taxon.

#### Chrysothrix candelaris

(L.) J.R. Laundon

4249F512-6B4B-5CC2-8BB9-42CA7E3DDF84

##### Materials

- **Type status:** Other material. **Occurrence:** recordedBy: R.T. McMullin; occurrenceID: 3D898DB4-FBE4-5C14-A0F1-B2C0BAC94289; **Location:** locationID: V; decimalLatitude: 51.62022; decimalLongitude: -127.93070; **Identification:** identificationRemarks: TLC: calycin and zeorin; **Event:** habitat: Corticolous on *Piceasitchensis*; **Record Level:** institutionID: CANL; collectionID: McMullin 19832
- **Type status:** Other material. **Occurrence:** recordedBy: R.T. McMullin; occurrenceID: 83E6C241-2754-591A-9061-B017DB303BA5; **Location:** locationID: IV; decimalLatitude: 51.65514; decimalLongitude: -128.13243; **Identification:** identificationRemarks: TLC: calycin and zeorin; **Event:** habitat: Corticolous on *Thujaplicata*; **Record Level:** institutionID: CANL; collectionID: McMullin 19833
- **Type status:** Other material. **Occurrence:** recordedBy: R.T. McMullin; occurrenceID: CD5414EE-2271-56F3-A920-76CF26D36DCE; **Location:** locationID: XIX; decimalLatitude: 51.65065; decimalLongitude: -128.14241; **Identification:** identificationRemarks: TLC: calycin and zeorin; **Event:** habitat: Corticolous on *Piceasitchensis*; **Record Level:** institutionID: CANL; collectionID: McMullin 19837
- **Type status:** Other material. **Occurrence:** recordedBy: R.T. McMullin; occurrenceID: F6187858-0C5A-5F14-AB69-EF165D07D120; **Location:** locationID: XVIII; decimalLatitude: 51.66476; decimalLongitude: -128.11798; **Identification:** identificationRemarks: TLC: calycin and zeorin; **Event:** habitat: Corticolous on *Thujaplicata*; **Record Level:** institutionID: CANL; collectionID: McMullin 19838
- **Type status:** Other material. **Occurrence:** recordedBy: R.T. McMullin; occurrenceID: B690015D-9E26-557E-A167-E21437B57F14; **Location:** locationID: XXVI; decimalLatitude: 51.65466; decimalLongitude: -128.13051; **Identification:** identificationRemarks: TLC: calycin and zeorin; **Event:** habitat: Corticolous on *Piceasitchensis*; **Record Level:** institutionID: CANL; collectionID: McMullin 19839
- **Type status:** Other material. **Occurrence:** recordedBy: R.T. McMullin; occurrenceID: CF8BF8EB-3F5B-52B1-B05B-3E1349B50B2C; **Location:** locationID: III; decimalLatitude: 51.65486; decimalLongitude: -128.13907; **Identification:** identificationRemarks: TLC: calycin and zeorin; **Event:** habitat: Corticolous on *Piceasitchensis*; **Record Level:** institutionID: CANL; collectionID: McMullin 19840
- **Type status:** Other material. **Occurrence:** catalogNumber: BOLD PHAK323-20; recordedBy: A. Simon; otherCatalogNumbers: GenBank OQ922986; occurrenceID: E8F63137-82A5-538F-91D6-0C15BAF7FB72; **Location:** locationID: XX; decimalLatitude: 51.64809; decimalLongitude: -128.14378; **Record Level:** institutionID: UBC; collectionID: Simon 780
- **Type status:** Other material. **Occurrence:** recordedBy: A. Simon; occurrenceID: 3931FCA1-0CF8-51C1-A605-F6E7CA72D7F2; **Location:** locationID: XIII; decimalLatitude: 51.66854; decimalLongitude: -128.11832; **Record Level:** institutionID: UBC; collectionID: Simon 785

#### Cladonia arbuscula

(Wallr.) Flot.

6CDF7ED2-2169-55A2-8B1E-DCE86265E430

##### Materials

- **Type status:** Other material. **Occurrence:** catalogNumber: BOLD CALV231-20; recordedBy: R.T. McMullin; otherCatalogNumbers: GenBank OQ843222; occurrenceID: 1D5E68C1-35C7-5AB0-B882-E6185A37052C; **Location:** locationID: VIII; decimalLatitude: 51.61611; decimalLongitude: -127.93962; **Identification:** identificationRemarks: TLC: fumarprotocetraric and usnic acids; **Event:** habitat: Terricolous; **Record Level:** institutionID: CANL; collectionID: McMullin 19598

#### Cladonia bellidiflora

(Ach.) Schaer.

B0055595-D5CD-599B-8115-6F75564711E3

##### Materials

- **Type status:** Other material. **Occurrence:** recordedBy: R.T. McMullin; occurrenceID: CAB4C729-4EA2-5E93-9D3B-679343E80AF6; **Location:** locationID: XX; decimalLatitude: 51.64809; decimalLongitude: -128.14378; **Event:** habitat: Terricolous; **Record Level:** institutionID: CANL; collectionID: McMullin 19509
- **Type status:** Other material. **Occurrence:** catalogNumber: BOLD CALV232-20; recordedBy: R.T. McMullin; otherCatalogNumbers: GenBank OQ843323; occurrenceID: 24269BD3-0E1F-5242-866B-FA9E1EB362E7; **Location:** locationID: V; decimalLatitude: 51.62022; decimalLongitude: -127.93070; **Event:** habitat: Terricolous; **Record Level:** institutionID: CANL; collectionID: McMullin 19714
- **Type status:** Other material. **Occurrence:** catalogNumber: BOLD CALV234-20; recordedBy: R.T. McMullin; otherCatalogNumbers: GenBank OQ843196; occurrenceID: 60B4E7A4-5814-5C20-9756-DC4698950B26; **Location:** locationID: IX; decimalLatitude: 51.61638; decimalLongitude: -127.94035; **Event:** habitat: Terricolous; **Record Level:** institutionID: CANL; collectionID: McMullin 19715
- **Type status:** Other material. **Occurrence:** recordedBy: R.T. McMullin; occurrenceID: 4E65A208-5C25-5B95-A736-685B3087B3B2; **Location:** locationID: XIX; decimalLatitude: 51.65065; decimalLongitude: -128.14241; **Event:** habitat: Corticolous on *Piceasitchensis*; **Record Level:** institutionID: CANL; collectionID: McMullin 19738
- **Type status:** Other material. **Occurrence:** catalogNumber: BOLD PHAK344-20; recordedBy: A. Simon; otherCatalogNumbers: GenBank OQ922990; occurrenceID: D95532F3-928D-555E-AC5C-799456274BDA; **Location:** locationID: XXX; decimalLatitude: 51.63722; decimalLongitude: -128.09525; **Record Level:** institutionID: UBC; collectionID: Simon 766
- **Type status:** Other material. **Occurrence:** catalogNumber: BOLD PHAK364-20; recordedBy: A. Simon; otherCatalogNumbers: GenBank OQ922948; occurrenceID: 85E3775F-0F3C-51EF-B4FF-6676655FC707; **Location:** locationID: IX; decimalLatitude: 51.61638; decimalLongitude: -127.94035; **Record Level:** institutionID: UBC; collectionID: Simon 823

##### Notes

All specimens are the thamnolic acid chemotype.

#### Cladonia chlorophaea

(Flörke ex Sommerf.) Spreng.

F8FAFFE2-40D9-5CA9-81EA-6E042481F6CD

##### Materials

- **Type status:** Other material. **Occurrence:** recordedBy: R.T. McMullin; occurrenceID: B4B4B5A5-A025-5A60-BEEE-5D419A68B5E1; **Location:** locationID: XXI; decimalLatitude: 51.64221; decimalLongitude: -128.15085; **Identification:** identificationRemarks: TLC: fumarprotocetraric acid; **Event:** habitat: Corticolous; **Record Level:** institutionID: CANL; collectionID: McMullin 19841

#### Cladonia ciliata f. flavicans

(Flörke) Ahti & DePriest (syn. Cladonia ciliata var. tenuis (Flörke) Ahti)

1893B16B-7289-5217-B5EC-325FFF6B5B98

##### Materials

- **Type status:** Other material. **Occurrence:** recordedBy: R.T. McMullin; occurrenceID: 34F2BACC-D649-54B1-8DB8-CB5BCC155448; **Location:** locationID: XX; decimalLatitude: 51.64809; decimalLongitude: -128.14378; **Identification:** identificationRemarks: TLC: fumarprotocetraric and usnic acids; **Event:** habitat: Terricolous; **Record Level:** institutionID: CANL; collectionID: McMullin 19545

#### Cladonia cornuta cornuta

(L.) Hoffm.

BDB1976A-F5FF-5DDD-9F14-60DE48939A8B

##### Materials

- **Type status:** Other material. **Occurrence:** catalogNumber: BOLD CALV238-20; recordedBy: R.T. McMullin; otherCatalogNumbers: GenBank OQ843265; occurrenceID: 06AD18B6-47F0-5739-A94A-EBB6AC7ABE99; **Location:** locationID: XX; decimalLatitude: 51.64809; decimalLongitude: -128.14378; **Event:** habitat: Terricolous; **Record Level:** institutionID: CANL; collectionID: McMullin 19569

#### Cladonia crispata var. cetrariiformis

(Delise) Vain.

3CB809DE-8CA9-5A8D-A733-DCE855C9CACD

##### Materials

- **Type status:** Other material. **Occurrence:** catalogNumber: BOLD PHAK293-20; recordedBy: A. Simon; otherCatalogNumbers: GenBank OQ922951; occurrenceID: 4C49AB78-D7C2-5CAD-91CB-8D3C08EC0961; **Location:** locationID: XXX; decimalLatitude: 51.63722; decimalLongitude: -128.09525; **Record Level:** institutionID: UBC; collectionID: Simon 767

#### Cladonia crispata var. crispata

(Ach.) Flot.

E8F641F5-404B-5175-ABE5-5439002747C6

##### Materials

- **Type status:** Other material. **Occurrence:** catalogNumber: BOLD PHAK300-20; recordedBy: A. Simon; otherCatalogNumbers: GenBank OQ922956; occurrenceID: 34E3DE94-3073-51B2-900E-596BF093FD6F; **Location:** locationID: XIII; decimalLatitude: 51.66854; decimalLongitude: -128.11832; **Record Level:** institutionID: UBC; collectionID: Simon 786

#### Cladonia furcata

(Huds.) Schrad.

0CB282AD-80FC-584A-85D4-390C91BD6AA9

##### Materials

- **Type status:** Other material. **Occurrence:** catalogNumber: BOLD CALV239-20; recordedBy: R.T. McMullin; otherCatalogNumbers: GenBank OQ843327; occurrenceID: C5F50F49-13ED-5F23-9261-894CE82F6E5E; **Location:** locationID: II; decimalLatitude: 51.65285; decimalLongitude: -128.13873; **Event:** habitat: Terricolous; **Record Level:** institutionID: CANL; collectionID: McMullin 19797

#### Cladonia gracilis vulnerata

Ahti

F5C78BD8-4CCF-5510-B847-C8C86ED8B333

##### Materials

- **Type status:** Other material. **Occurrence:** catalogNumber: BOLD CALV240-20; recordedBy: R.T. McMullin; otherCatalogNumbers: GenBank OQ843311; occurrenceID: F6638CDA-0B6B-5237-8100-E59383E23D8F; **Location:** locationID: XX; decimalLatitude: 51.64809; decimalLongitude: -128.14378; **Event:** habitat: Terricolous; **Record Level:** institutionID: CANL; collectionID: McMullin 19517
- **Type status:** Other material. **Occurrence:** recordedBy: R.T. McMullin; occurrenceID: C9EC0B3B-445A-54CF-97A4-AC0D0CF90875; **Location:** locationID: XXV; decimalLatitude: 51.65180; decimalLongitude: -128.12865; **Event:** habitat: Terricolous; **Record Level:** institutionID: CANL; collectionID: McMullin 19532

#### Cladonia grayi

G. Merr. ex Sandst.

9FF08D6E-DEEC-588D-BF44-E1DDDD4738CC

##### Materials

- **Type status:** Other material. **Occurrence:** catalogNumber: BOLD CALV242-20; recordedBy: R.T. McMullin; otherCatalogNumbers: GenBank OQ843335; occurrenceID: 9F0C1D32-5787-5E28-AD19-6388B128A7FA; **Location:** locationID: XX; decimalLatitude: 51.64809; decimalLongitude: -128.14378; **Identification:** identificationRemarks: TLC: fumarprotocetraric and grayanic acids; **Event:** habitat: Terricolous; **Record Level:** institutionID: CANL; collectionID: McMullin 19843

#### Cladonia ochrochlora

Flörke

5FCC6C20-8D5A-5A88-B1A2-25939BC4CEB9

##### Materials

- **Type status:** Other material. **Occurrence:** catalogNumber: BOLD CALV243-20; recordedBy: R.T. McMullin; otherCatalogNumbers: GenBank OQ843346; occurrenceID: 1F22622F-B85B-5AC6-8FD2-210040B935C6; **Location:** locationID: XII; decimalLatitude: 51.66040; decimalLongitude: -128.11688; **Event:** habitat: Terricolous; **Record Level:** institutionID: CANL; collectionID: McMullin 19568
- **Type status:** Other material. **Occurrence:** recordedBy: R.T. McMullin; occurrenceID: 9083DE39-F349-5DE8-BA6C-1C2C496BE11E; **Location:** locationID: V; decimalLatitude: 51.62022; decimalLongitude: -127.93070; **Identification:** identificationRemarks: TLC: fumarprotocetraric acid; **Event:** habitat: Corticolous; **Record Level:** institutionID: CANL; collectionID: McMullin 19705

#### Cladonia pleurota

(Flörke) Schaer.

8F2DED16-AC63-5848-836D-D7E9ED72150E

##### Materials

- **Type status:** Other material. **Occurrence:** catalogNumber: BOLD CALV245-20; recordedBy: R.T. McMullin; otherCatalogNumbers: GenBank OQ843315; occurrenceID: 63E1FBD9-5485-562F-A7E9-3B2D310F5744; **Location:** locationID: XIV; decimalLatitude: 51.66797; decimalLongitude: -128.12128; **Identification:** identificationRemarks: TLC: usnic acid and zeorin; **Event:** habitat: Terricolous; **Record Level:** institutionID: CANL; collectionID: McMullin 19670

#### Cladonia portentosa pacifica

(Ahti) Ahti

47644E4F-122C-5E06-8EB0-BFC4886C5D49

##### Materials

- **Type status:** Other material. **Occurrence:** recordedBy: R.T. McMullin; occurrenceID: 305E0DAB-0F59-580F-8162-58D44710A553; **Location:** locationID: XX; decimalLatitude: 51.64809; decimalLongitude: -128.14378; **Event:** habitat: Terricolous; **Record Level:** institutionID: CANL; collectionID: McMullin 19520
- **Type status:** Other material. **Occurrence:** recordedBy: R.T. McMullin; occurrenceID: 71F787DD-8261-5D79-8E5D-C92B6F397399; **Location:** locationID: XI; decimalLatitude: 51.61622; decimalLongitude: -127.94227; **Event:** habitat: Terricolous; **Record Level:** institutionID: CANL; collectionID: McMullin 19599
- **Type status:** Other material. **Occurrence:** catalogNumber: BOLD CALV246-20; recordedBy: R.T. McMullin; otherCatalogNumbers: GenBank OQ843347; occurrenceID: 16243E51-5894-527A-A474-4BB572085DE5; **Location:** locationID: II; decimalLatitude: 51.65285; decimalLongitude: -128.13873; **Event:** habitat: Terricolous; **Record Level:** institutionID: CANL; collectionID: McMullin 19772
- **Type status:** Other material. **Occurrence:** recordedBy: R.T. McMullin; occurrenceID: 24541784-95BA-5E77-A637-9B608D553E6D; **Location:** locationID: XXV; decimalLatitude: 51.65180; decimalLongitude: -128.12865; **Event:** habitat: Terricolous; **Record Level:** institutionID: CANL; collectionID: McMullin 19868
- **Type status:** Other material. **Occurrence:** recordedBy: A. Simon; occurrenceID: EAFBB802-7EDD-533D-99E1-0AD47542FC11; **Location:** locationID: XXV; decimalLatitude: 51.65180; decimalLongitude: -128.12865; **Record Level:** institutionID: UBC; collectionID: Simon 763
- **Type status:** Other material. **Occurrence:** recordedBy: A. Simon; occurrenceID: 18AE2246-7B47-5743-BFCD-E0CB572A87FA; **Location:** locationID: XXII; decimalLatitude: 51.64502; decimalLongitude: -128.15099; **Record Level:** institutionID: UBC; collectionID: Simon 806
- **Type status:** Other material. **Occurrence:** recordedBy: A. Simon; occurrenceID: 6CE70ABF-BA99-5A17-AF7B-B80AD61B4DB9; **Location:** locationID: IX; decimalLatitude: 51.61638; decimalLongitude: -127.94035; **Record Level:** institutionID: UBC; collectionID: Simon 821

#### Cladonia rangiferina

(L.) F.H. Wigg.

8F706775-898A-56AA-99CE-D68C6F4A88FB

##### Materials

- **Type status:** Other material. **Occurrence:** recordedBy: R.T. McMullin; occurrenceID: 30EB7E57-3612-5590-969F-F1FE76CE9E99; **Location:** locationID: VIII; decimalLatitude: 51.61611; decimalLongitude: -127.93962; **Event:** habitat: Terricolous; **Record Level:** institutionID: CANL; collectionID: McMullin 19625
- **Type status:** Other material. **Occurrence:** recordedBy: A. Simon; occurrenceID: 6D66A960-2AA0-5245-B85D-C92AB770126F; **Location:** locationID: XXV; decimalLatitude: 51.65180; decimalLongitude: -128.12865; **Record Level:** institutionID: UBC; collectionID: Simon 764
- **Type status:** Other material. **Occurrence:** catalogNumber: BOLD PHAK365-20; recordedBy: A. Simon; otherCatalogNumbers: GenBank OQ922974; occurrenceID: 70962FEC-D4B4-5C1F-B853-CB4798CE4773; **Location:** locationID: XI; decimalLatitude: 51.61622; decimalLongitude: -127.94227; **Record Level:** institutionID: UBC; collectionID: Simon 822

#### Cladonia scabriuscula

(Delise) Nyl.

F6C0F898-29AD-56DD-9753-8B51D8137F17

##### Materials

- **Type status:** Other material. **Occurrence:** catalogNumber: BOLD PHAK339-20; recordedBy: A. Simon; otherCatalogNumbers: GenBank OQ922989; occurrenceID: 59AA7BD8-1EDA-555E-910B-25C4E099086E; **Location:** locationID: XXVIII; decimalLatitude: 51.66425; decimalLongitude: -128.12706; **Record Level:** institutionID: UBC; collectionID: Simon 750

#### Cladonia squamosa var. squamosa

Hoffm.

D95A4C1A-1E74-5E64-B85B-A3B2591122BB

##### Materials

- **Type status:** Other material. **Occurrence:** recordedBy: R.T. McMullin; occurrenceID: 1AF8B95A-713D-536F-B7BB-89D3A9074996; **Location:** locationID: III; decimalLatitude: 51.65486; decimalLongitude: -128.13907; **Event:** habitat: Terricolous; **Record Level:** institutionID: CANL; collectionID: McMullin 19539
- **Type status:** Other material. **Occurrence:** recordedBy: R.T. McMullin; occurrenceID: C243C8EA-D984-523F-83C7-EAA900438F88; **Location:** locationID: II; decimalLatitude: 51.65285; decimalLongitude: -128.13873; **Event:** habitat: Terricolous; **Record Level:** institutionID: CANL; collectionID: McMullin 19762
- **Type status:** Other material. **Occurrence:** catalogNumber: BOLD CALV251-20; recordedBy: R.T. McMullin; otherCatalogNumbers: GenBank OQ843305; occurrenceID: A358A7D0-ECA4-5CFC-AEC1-93256D95A8A2; **Location:** locationID: XXVI; decimalLatitude: 51.65466; decimalLongitude: -128.13051; **Event:** habitat: Corticolous on the base of a *Piceasitchensis*; **Record Level:** institutionID: CANL; collectionID: McMullin 19765

##### Notes

All specimens are the squamatic acid chemotype.

#### Cladonia straminea

(Sommerf.) Flörke

25A4902D-17E9-5365-B546-1C3A3319C641

##### Materials

- **Type status:** Other material. **Occurrence:** catalogNumber: BOLD CALV253-20; recordedBy: R.T. McMullin; otherCatalogNumbers: GenBank OQ843354; occurrenceID: 8F7B4F64-7248-5E8B-AB86-D74AF790BE9C; **Location:** locationID: XII; decimalLatitude: 51.66040; decimalLongitude: -128.11688; **Identification:** identificationRemarks: TLC: didymic, squamatic, and usnic acids; **Event:** habitat: Terricolous; **Record Level:** institutionID: CANL; collectionID: McMullin 19709
- **Type status:** Other material. **Occurrence:** recordedBy: R.T. McMullin; occurrenceID: 0E6D759B-E481-51A2-8034-C7B598CF1D35; **Location:** locationID: XX; decimalLatitude: 51.64809; decimalLongitude: -128.14378; **Identification:** identificationRemarks: TLC: didymic, squamatic, and usnic acids; **Event:** habitat: Terricolous; **Record Level:** institutionID: CANL; collectionID: McMullin 19809
- **Type status:** Other material. **Occurrence:** catalogNumber: BOLD CALV254-20; recordedBy: R.T. McMullin; otherCatalogNumbers: GenBank OQ843225; occurrenceID: 425DCA5F-ED01-5683-89E6-6C24A3AB8B6B; **Location:** locationID: XXV; decimalLatitude: 51.65180; decimalLongitude: -128.12865; **Identification:** identificationRemarks: TLC: didymic, squamatic, and usnic acids; **Event:** habitat: Terricolous; **Record Level:** institutionID: CANL; collectionID: McMullin 19844
- **Type status:** Other material. **Occurrence:** catalogNumber: BOLD CALV252-20; recordedBy: R.T. McMullin; otherCatalogNumbers: GenBank OQ843229; occurrenceID: 1DAC8CDF-7817-53CB-AC19-4B4BA2F67E99; **Location:** locationID: XX; decimalLatitude: 51.64809; decimalLongitude: -128.14378; **Identification:** identificationRemarks: TLC: didymic, squamatic, and usnic acids; **Event:** habitat: Terricolous; **Record Level:** institutionID: CANL; collectionID: McMullin 19845

#### Cladonia umbricola

Tønsberg & Ahti

14638BB0-3935-59F3-8016-C5F0A6CA975A

##### Materials

- **Type status:** Other material. **Occurrence:** recordedBy: Schofield; occurrenceID: 89CA8D9A-C96B-5CDD-A5CD-61611E069D90; **Location:** locationID: Safety Cove; **Event:** habitat: Terricolous; **Record Level:** institutionID: UBC; collectionID: Schofield 408262

##### Notes

Collected on Calvert Island prior to our survey, but we did not locate it.

#### Cladonia uncialis uncialis

(L.) F.H. Wigg.

151893A5-99C5-5D77-93C1-4DE71F487A44

##### Materials

- **Type status:** Other material. **Occurrence:** catalogNumber: BOLD CALV255-20; recordedBy: R.T. McMullin; otherCatalogNumbers: GenBank OQ843227; occurrenceID: 9139BD89-6F2E-5C05-9D01-D39327BA8095; **Location:** locationID: XX; decimalLatitude: 51.64809; decimalLongitude: -128.14378; **Event:** habitat: Terricolous; **Record Level:** institutionID: CANL; collectionID: McMullin 19558
- **Type status:** Other material. **Occurrence:** catalogNumber: BOLD CALV256-20; recordedBy: R.T. McMullin; otherCatalogNumbers: GenBank OQ843270; occurrenceID: 9029D600-0FB5-526B-A025-C9309E05A639; **Location:** locationID: XI; decimalLatitude: 51.61622; decimalLongitude: -127.94227; **Event:** habitat: Terricolous; **Record Level:** institutionID: CANL; collectionID: McMullin 19589
- **Type status:** Other material. **Occurrence:** recordedBy: A. Simon; occurrenceID: 8F0C7C81-E119-5014-AD74-6A3D963CBE44; **Location:** locationID: XXV; decimalLatitude: 51.65180; decimalLongitude: -128.12865; **Record Level:** institutionID: UBC; collectionID: Simon 765

#### Cladonia wainioi

Savicz

BA5D33A2-E852-51AA-AF27-39597F79001A

##### Materials

- **Type status:** Other material. **Occurrence:** catalogNumber: BOLD CALV257-20; recordedBy: R.T. McMullin; otherCatalogNumbers: GenBank OQ843364; occurrenceID: 6D59EC98-D8F0-51C3-98B9-10D512983D71; **Location:** locationID: XX; decimalLatitude: 51.64809; decimalLongitude: -128.14378; **Event:** habitat: Terricolous; **Record Level:** institutionID: CANL; collectionID: McMullin 19518
- **Type status:** Other material. **Occurrence:** catalogNumber: BOLD CALV258-20; recordedBy: R.T. McMullin; otherCatalogNumbers: GenBank OQ843257; occurrenceID: F5062931-6CCC-50C9-AE05-F41F3DA2F28C; **Location:** locationID: XX; decimalLatitude: 51.64809; decimalLongitude: -128.14378; **Event:** habitat: Terricolous; **Record Level:** institutionID: CANL; collectionID: McMullin 19557

#### Cliostomum leprosum

(Räsänen) Holien & Tønsberg

0BE2E2FF-2540-5647-B839-4127536B0C8B

##### Materials

- **Type status:** Other material. **Occurrence:** recordedBy: R.T. McMullin; occurrenceID: 2EFB36CF-809C-513B-A71E-00E779B69BC6; **Location:** locationID: VIII; decimalLatitude: 51.61611; decimalLongitude: -127.93962; **Event:** habitat: Corticolous on *Thujaplicata*; **Record Level:** institutionID: CANL; collectionID: McMullin 19710

#### Coccotrema maritimum

Brodo

05070FAC-A697-506D-B465-ABD1A3381037

##### Materials

- **Type status:** Other material. **Occurrence:** catalogNumber: BOLD CALV259-20; recordedBy: R.T. McMullin; otherCatalogNumbers: GenBank OQ843289; occurrenceID: D4EFC6FA-7D49-5090-AD24-E461E046B782; **Location:** locationID: II; decimalLatitude: 51.65285; decimalLongitude: -128.13873; **Identification:** identificationRemarks: Figure 2F; **Event:** habitat: Saxicolous; **Record Level:** institutionID: CANL; collectionID: McMullin 19750
- **Type status:** Other material. **Occurrence:** catalogNumber: BOLD PHAK315-20; recordedBy: A. Simon; otherCatalogNumbers: GenBank OQ922992; occurrenceID: 77C46E5D-262C-52D8-AF23-FFCB33A605B9; **Location:** locationID: II; decimalLatitude: 51.65285; decimalLongitude: -128.13873; **Record Level:** institutionID: UBC; collectionID: Simon 751
- **Type status:** Other material. **Occurrence:** recordedBy: A. Simon; occurrenceID: 82145624-A18D-5E47-B774-0F1ABA1F1205; **Location:** locationID: V; decimalLatitude: 51.62022; decimalLongitude: -127.93070; **Record Level:** institutionID: UBC; collectionID: Simon 824

#### Coccotrema pocillarium

(C.E. Cumm.) Brodo

0C857D78-78C8-5A53-BAE0-8DA0908A2DE7

##### Materials

- **Type status:** Other material. **Occurrence:** catalogNumber: BOLD CALV260-20; recordedBy: R.T. McMullin; otherCatalogNumbers: GenBank OQ843230; occurrenceID: FF664ABD-2CDF-5304-B825-D5F31AAF8997; **Location:** locationID: XIII; decimalLatitude: 51.66854; decimalLongitude: -128.11832; **Event:** habitat: Corticolous on *Piceasitchensis*; **Record Level:** institutionID: CANL; collectionID: McMullin 19571
- **Type status:** Other material. **Occurrence:** catalogNumber: BOLD CALV261-20; recordedBy: R.T. McMullin; otherCatalogNumbers: GenBank OQ843247; occurrenceID: 69E520A5-E184-5930-8F91-C1B4E7EE8639; **Location:** locationID: V; decimalLatitude: 51.62022; decimalLongitude: -127.93070; **Event:** habitat: Corticolous on a conifer snag; **Record Level:** institutionID: CANL; collectionID: McMullin 19623
- **Type status:** Other material. **Occurrence:** catalogNumber: BOLD CALV262-20; recordedBy: R.T. McMullin; otherCatalogNumbers: GenBank OQ843332; occurrenceID: ED8F91BC-2753-5AF1-A6F1-C53D23F96A71; **Location:** locationID: III; decimalLatitude: 51.65486; decimalLongitude: -128.13907; **Identification:** identificationRemarks: TLC: stictic acid; **Event:** habitat: Corticolous on a snag; **Record Level:** institutionID: CANL; collectionID: McMullin 19733
- **Type status:** Other material. **Occurrence:** catalogNumber: BOLD PHAK331-20; recordedBy: A. Simon; otherCatalogNumbers: GenBank OQ922977; occurrenceID: 6AC95A34-D624-554B-817C-124186DB5462; **Location:** locationID: XIV; decimalLatitude: 51.66797; decimalLongitude: -128.12128; **Record Level:** institutionID: UBC; collectionID: Simon 787
- **Type status:** Other material. **Occurrence:** recordedBy: A. Simon; occurrenceID: 45ACEB54-8737-5BDB-A001-D8AA61BC3784; **Location:** locationID: XXI; decimalLatitude: 51.64221; decimalLongitude: -128.15085; **Record Level:** institutionID: UBC; collectionID: Simon 807
- **Type status:** Other material. **Occurrence:** catalogNumber: BOLD PHAK332-20; recordedBy: A. Simon; otherCatalogNumbers: GenBank OQ922964; occurrenceID: 1F476120-F050-579A-833A-05DF835050F7; **Location:** locationID: XXIII; decimalLatitude: 51.64058; decimalLongitude: -128.14882; **Record Level:** institutionID: UBC; collectionID: Simon 841
- **Type status:** Other material. **Occurrence:** recordedBy: A. Simon; occurrenceID: 3E309E09-37C1-5826-A681-DF8B1786EFD4; **Location:** locationID: XXIII; decimalLatitude: 51.64058; decimalLongitude: -128.14882; **Record Level:** institutionID: UBC; collectionID: Simon 842

#### Collema subflaccidum

Degel.

A12F32B3-BE5F-5936-8D2C-6B56B34013A5

##### Materials

- **Type status:** Other material. **Occurrence:** recordedBy: A. Simon; occurrenceID: 4629BF70-9E89-5912-8B19-C95CF51720B8; **Location:** locationID: XXIII; decimalLatitude: 51.64058; decimalLongitude: -128.14882; **Record Level:** institutionID: UBC; collectionID: Simon 843

#### Collemopsidium foveolatum

(A.L. Sm.) F. Mohr.

CB79B5B4-3AE1-5552-9E41-E274982C242F

##### Materials

- **Type status:** Other material. **Occurrence:** recordedBy: R.T. McMullin; occurrenceID: 34B44369-1DCE-56CC-B63C-BC92B1A154EE; **Location:** locationID: V; decimalLatitude: 51.62022; decimalLongitude: -127.93070; **Event:** habitat: Calcicolous on a barnacle; **Record Level:** institutionID: CANL; collectionID: McMullin 19679
- **Type status:** Other material. **Occurrence:** recordedBy: A. Simon; occurrenceID: EE91DCBA-9250-587E-AB39-863DA19015CF; **Location:** locationID: V; decimalLatitude: 51.62022; decimalLongitude: -127.93070; **Record Level:** institutionID: UBC; collectionID: Simon 825

#### Ephebe lanata

(L.) Vain.

A8DB0EB4-9346-5015-8320-C13D440F9161

##### Materials

- **Type status:** Other material. **Occurrence:** recordedBy: Schofield; occurrenceID: 87853255-4715-5971-94AB-297FB5FCC1E3; **Location:** locationID: Keith Anchorage Area, Kwakshua Channel; **Event:** habitat: Saxicolous; **Record Level:** institutionID: UBC; collectionID: Schofield 27007

##### Notes

Collected on Calvert Island prior to our survey, but we did not locate it.

#### Felipes leucopellaeus

(Ach.) Frisch & G. Thor

E3D1BDD4-0E3B-5201-AAF5-DC769CDD415A

##### Materials

- **Type status:** Other material. **Occurrence:** catalogNumber: BOLD CALV264-20; recordedBy: R.T. McMullin; otherCatalogNumbers: GenBank OQ843307; occurrenceID: 2EA92798-E66C-5570-8F4C-C038FDD00869; **Location:** locationID: XXVI; decimalLatitude: 51.65466; decimalLongitude: -128.13051; **Identification:** identificationRemarks: TLC: no substance detected; **Event:** habitat: Corticolous on *Thujaplicata*; **Record Level:** institutionID: CANL; collectionID: McMullin 19779

#### Fuscopannaria pacifica

P.M. Jørg.

4490BCE3-4A73-5C18-8A15-376E092A2D53

##### Materials

- **Type status:** Other material. **Occurrence:** catalogNumber: BOLD CALV268-20; recordedBy: R.T. McMullin; otherCatalogNumbers: GenBank OQ843365; occurrenceID: FD006A69-978A-560B-97A6-901F077A9203; **Location:** locationID: XVI; decimalLatitude: 51.66051; decimalLongitude: -128.14587; **Identification:** identificationRemarks: TLC: atranorin absent; **Event:** habitat: Corticolous on *Alnusrubra*; **Record Level:** institutionID: CANL; collectionID: McMullin 19578
- **Type status:** Other material. **Occurrence:** catalogNumber: BOLD CALV180-20; recordedBy: R.T. McMullin; otherCatalogNumbers: GenBank OQ843273; occurrenceID: B77814C6-5F97-5E56-BD67-3DDAFB9E58C8; **Location:** locationID: V; decimalLatitude: 51.62022; decimalLongitude: -127.93070; **Event:** habitat: Saxicolous; **Record Level:** institutionID: CANL; collectionID: McMullin 19652
- **Type status:** Other material. **Occurrence:** catalogNumber: BOLD CALV271-20; recordedBy: R.T. McMullin; otherCatalogNumbers: GenBank OQ843250; occurrenceID: 67DA3783-C1C2-5099-B307-B8BB1BE601CC; **Location:** locationID: V; decimalLatitude: 51.62022; decimalLongitude: -127.93070; **Event:** habitat: Saxicolous; **Record Level:** institutionID: CANL; collectionID: McMullin 19685
- **Type status:** Other material. **Occurrence:** recordedBy: A. Simon; occurrenceID: DEE891CC-1CA4-5D82-8C60-E925E8EDA13F; **Location:** locationID: II; decimalLatitude: 51.65285; decimalLongitude: -128.13873; **Record Level:** institutionID: UBC; collectionID: Simon 752
- **Type status:** Other material. **Occurrence:** catalogNumber: BOLD PHAK356-20; recordedBy: A. Simon; otherCatalogNumbers: GenBank OQ922966; occurrenceID: 602C7FCC-3CC6-5B68-A7B0-7F4E771BF80B; **Location:** locationID: V; decimalLatitude: 51.62022; decimalLongitude: -127.93070; **Record Level:** institutionID: UBC; collectionID: Simon 826

#### Graphis elegans

(Borrer ex Sm.) Ach.

B5252A81-6F4C-54B7-A668-B54E93E162EA

##### Materials

- **Type status:** Other material. **Occurrence:** catalogNumber: BOLD CALV162-20; recordedBy: R.T. McMullin; otherCatalogNumbers: GenBank OQ843338; occurrenceID: A92DF64F-7127-57BC-A4D5-23E9AEDBF588; **Location:** locationID: IV; decimalLatitude: 51.65514; decimalLongitude: -128.13243; **Identification:** identificationRemarks: TLC: norstictic acid; **Event:** habitat: Corticolous on *Alnusrubra*; **Record Level:** institutionID: CANL; collectionID: McMullin 19882

#### Herteliana alaskensis

(Nyl.) S. Ekman

07ED6B7D-FD25-513B-B9B6-92E5B7312543

##### Materials

- **Type status:** Other material. **Occurrence:** catalogNumber: BOLD CALV159-20; recordedBy: R.T. McMullin; otherCatalogNumbers: GenBank OQ843210; occurrenceID: F11C6D5A-D582-5A55-8034-355FCF5CE7CC; **Location:** locationID: V; decimalLatitude: 51.62022; decimalLongitude: -127.93070; **Event:** habitat: Saxicolous; **Record Level:** institutionID: CANL; collectionID: McMullin 19608

#### Hertelidea botryosa

(Fr.) Printzen & Kantvilas

B543D37A-925E-5EFF-B02D-52C5AB6E78B9

##### Materials

- **Type status:** Other material. **Occurrence:** recordedBy: R.T. McMullin; occurrenceID: 154D1DDA-0EB9-53E3-9385-77FD0F954B6C; **Location:** locationID: XIX; decimalLatitude: 51.65065; decimalLongitude: -128.14241; **Identification:** identificationRemarks: TLC: perlatolic acid; **Event:** habitat: Saxicolous; **Record Level:** institutionID: CANL; collectionID: McMullin 19788

#### Hydropunctaria maura

(Wahlenb.) C. Keller, Gueidan & Thüs

6E5926A2-9F23-542B-A322-14AC248B328C

##### Materials

- **Type status:** Other material. **Occurrence:** catalogNumber: BOLD PHAK327-20; recordedBy: A. Simon; otherCatalogNumbers: GenBank OQ922994; occurrenceID: 833DEA04-BDA1-554E-9A6E-D29550EB94AA; **Location:** locationID: XIV; decimalLatitude: 51.66797; decimalLongitude: -128.12128; **Record Level:** institutionID: UBC; collectionID: Simon 781

#### Hypogymnia apinnata

Goward & McCune

7E2F41F7-3B1E-556E-A0C7-C52B3DA73B97

##### Materials

- **Type status:** Other material. **Occurrence:** catalogNumber: BOLD CALV273-20; recordedBy: R.T. McMullin; otherCatalogNumbers: GenBank OQ843343; occurrenceID: 8FF22C06-B94A-5A1F-9181-C2E5B4F7EA50; **Location:** locationID: XX; decimalLatitude: 51.64809; decimalLongitude: -128.14378; **Event:** habitat: Corticolous on Pinuscontortassp.contorta; **Record Level:** institutionID: CANL; collectionID: McMullin 19514
- **Type status:** Other material. **Occurrence:** catalogNumber: BOLD CALV285-20; recordedBy: R.T. McMullin; otherCatalogNumbers: GenBank OQ843286; occurrenceID: 438A303D-D28B-58B7-96BD-29403815F74D; **Location:** locationID: XXIII; decimalLatitude: 51.64058; decimalLongitude: -128.14882; **Event:** habitat: Corticolous on *Piceasitchensis*; **Record Level:** institutionID: CANL; collectionID: McMullin 19588
- **Type status:** Other material. **Occurrence:** recordedBy: R.T. McMullin; occurrenceID: 68D6B54D-6F0A-567F-8784-2706378F28FB; **Location:** locationID: XIII; decimalLatitude: 51.66854; decimalLongitude: -128.11832; **Event:** habitat: Corticolous on *Piceasitchensis*; **Record Level:** institutionID: CANL; collectionID: McMullin 19618
- **Type status:** Other material. **Occurrence:** catalogNumber: BOLD CALV160-20; recordedBy: R.T. McMullin; otherCatalogNumbers: GenBank OQ843313; occurrenceID: 25440590-2C20-5D5E-B51E-81A08E264A81; **Location:** locationID: XXVII; decimalLatitude: 51.65589; decimalLongitude: -128.13098; **Event:** habitat: Corticolous on a snag; **Record Level:** institutionID: CANL; collectionID: McMullin 19799
- **Type status:** Other material. **Occurrence:** catalogNumber: BOLD PHAK371-20; recordedBy: A. Simon; otherCatalogNumbers: GenBank OQ922950; occurrenceID: 96C4AB68-F54A-5C27-9025-7B1A77E566DA; **Location:** locationID: XXIII; decimalLatitude: 51.64058; decimalLongitude: -128.14882; **Record Level:** institutionID: UBC; collectionID: Simon 845

#### Hypogymnia duplicata

(Ach.) Rass.

7622DAA3-0259-5A5C-B38D-AAC226820C42

##### Materials

- **Type status:** Other material. **Occurrence:** recordedBy: R.T. McMullin; occurrenceID: 09156575-A0B3-56C9-B4B0-909A96C4B0EF; **Location:** locationID: XIV; decimalLatitude: 51.66797; decimalLongitude: -128.12128; **Event:** habitat: Corticolous on a conifer; **Record Level:** institutionID: CANL; collectionID: McMullin 19582
- **Type status:** Other material. **Occurrence:** recordedBy: R.T. McMullin; occurrenceID: ACA93DC6-91E7-5AFD-BF8B-070247658A6D; **Location:** locationID: XIV; decimalLatitude: 51.66797; decimalLongitude: -128.12128; **Event:** habitat: Corticolous on a conifer; **Record Level:** institutionID: CANL; collectionID: McMullin 19610
- **Type status:** Other material. **Occurrence:** catalogNumber: BOLD CALV274-20; recordedBy: R.T. McMullin; otherCatalogNumbers: GenBank OQ843296; occurrenceID: AA223E6C-944F-50C6-A174-BA38148415CD; **Location:** locationID: XIX; decimalLatitude: 51.65065; decimalLongitude: -128.14241; **Event:** habitat: Corticolous on a snag; **Record Level:** institutionID: CANL; collectionID: McMullin 19723
- **Type status:** Other material. **Occurrence:** recordedBy: R.T. McMullin; occurrenceID: B599A549-65C3-5D80-BFCF-E2C9BFB5C510; **Location:** locationID: XXIV; decimalLatitude: 51.65271; decimalLongitude: -128.12951; **Event:** habitat: Corticolous on *Thujaplicata*; **Record Level:** institutionID: CANL; collectionID: McMullin 19743
- **Type status:** Other material. **Occurrence:** recordedBy: R.T. McMullin; occurrenceID: 25A2C609-A536-5F25-9B27-E616E3A0906B; **Location:** locationID: XVIII; decimalLatitude: 51.66476; decimalLongitude: -128.11798; **Event:** habitat: Corticolous on *Thujaplicata*; **Record Level:** institutionID: CANL; collectionID: McMullin 19769

#### Hypogymnia enteromorpha

(Ach.) Nyl.

F5372920-DC24-5E3A-AA54-A7394CFB559B

##### Materials

- **Type status:** Other material. **Occurrence:** catalogNumber: BOLD CALV277-20; recordedBy: R.T. McMullin; otherCatalogNumbers: GenBank OQ843217; occurrenceID: 2A6D771C-61B6-56FB-88D9-16A7773C1DBB; **Location:** locationID: XI; decimalLatitude: 51.61622; decimalLongitude: -127.94227; **Event:** habitat: Corticolous on *Alnusrubra*; **Record Level:** institutionID: CANL; collectionID: McMullin 19631
- **Type status:** Other material. **Occurrence:** recordedBy: A. Simon; occurrenceID: 75AC6E8E-5976-5175-BA27-39433AE2415B; **Location:** locationID: XXIX; decimalLatitude: 51.65271; decimalLongitude: -128.12951; **Record Level:** institutionID: UBC; collectionID: Simon 753

#### Hypogymnia hultenii

(Degel.) Krog

5C461BAB-2799-5513-A45F-2A034491CF10

##### Materials

- **Type status:** Other material. **Occurrence:** catalogNumber: BOLD CALV278-20; recordedBy: R.T. McMullin; otherCatalogNumbers: GenBank OQ843345; occurrenceID: 615A1ADE-C8C9-5634-AACD-C6E0BC6AC6A7; **Location:** locationID: XVIII; decimalLatitude: 51.66476; decimalLongitude: -128.11798; **Event:** habitat: Corticolous on *Piceasitchensis*; **Record Level:** institutionID: CANL; collectionID: McMullin 19576
- **Type status:** Other material. **Occurrence:** catalogNumber: BOLD CALV279-20; recordedBy: R.T. McMullin; otherCatalogNumbers: GenBank OQ843255; occurrenceID: 5DBC611F-9F50-5731-AA40-E539566859A1; **Location:** locationID: XIII; decimalLatitude: 51.66854; decimalLongitude: -128.11832; **Event:** habitat: Corticolous on *Alnusrubra*; **Record Level:** institutionID: CANL; collectionID: McMullin 19713
- **Type status:** Other material. **Occurrence:** catalogNumber: BOLD CALV150-20; recordedBy: R.T. McMullin; otherCatalogNumbers: GenBank OQ843309; occurrenceID: 77939DAF-81DE-5D71-A172-52AAC0EEE20D; **Location:** locationID: II; decimalLatitude: 51.65285; decimalLongitude: -128.13873; **Event:** habitat: Corticolous on *Piceasitchensis*; **Record Level:** institutionID: CANL; collectionID: McMullin 19751
- **Type status:** Other material. **Occurrence:** catalogNumber: BOLD PHAK320-20; recordedBy: A. Simon; otherCatalogNumbers: GenBank OQ922961; occurrenceID: FDDDD5DD-C321-5874-B693-EC1160E00A76; **Location:** locationID: XXI; decimalLatitude: 51.64221; decimalLongitude: -128.15085; **Record Level:** institutionID: UBC; collectionID: Simon 805
- **Type status:** Other material. **Occurrence:** catalogNumber: BOLD PHAK360-20; recordedBy: A. Simon; otherCatalogNumbers: GenBank OQ922993; occurrenceID: D6A9307B-1995-5DA9-B512-C6A318A5A0DE; **Location:** locationID: XII; decimalLatitude: 51.66040; decimalLongitude: -128.11688; **Record Level:** institutionID: UBC; collectionID: Simon 840

#### Hypogymnia cf. imshaugii

Krog

5EAE3A4A-6CCF-502E-87ED-03665C51A0D2

##### Materials

- **Type status:** Other material. **Occurrence:** catalogNumber: BOLD CALV280-20; recordedBy: R.T. McMullin; otherCatalogNumbers: GenBank OQ843213; occurrenceID: 50472DAB-C6ED-58EE-B3CD-113449870A78; **Location:** locationID: XX; decimalLatitude: 51.64809; decimalLongitude: -128.14378; **Event:** habitat: Corticolous on Pinuscontortassp.contorta; **Record Level:** institutionID: CANL; collectionID: McMullin 19513
- **Type status:** Other material. **Occurrence:** catalogNumber: BOLD CALV281-20; recordedBy: R.T. McMullin; otherCatalogNumbers: GenBank OQ843244; occurrenceID: F09945D0-CDD7-5939-BACF-3801294A5E61; **Location:** locationID: XII; decimalLatitude: 51.66040; decimalLongitude: -128.11688; **Event:** habitat: Corticolous on Pinuscontortassp.contorta; **Record Level:** institutionID: CANL; collectionID: McMullin 19637

#### Hypogymnia lophyrea

(Ach.) Krog

ABC10F35-3D04-5FF9-942D-0B3CB533DC8B

##### Materials

**Figure 3.**
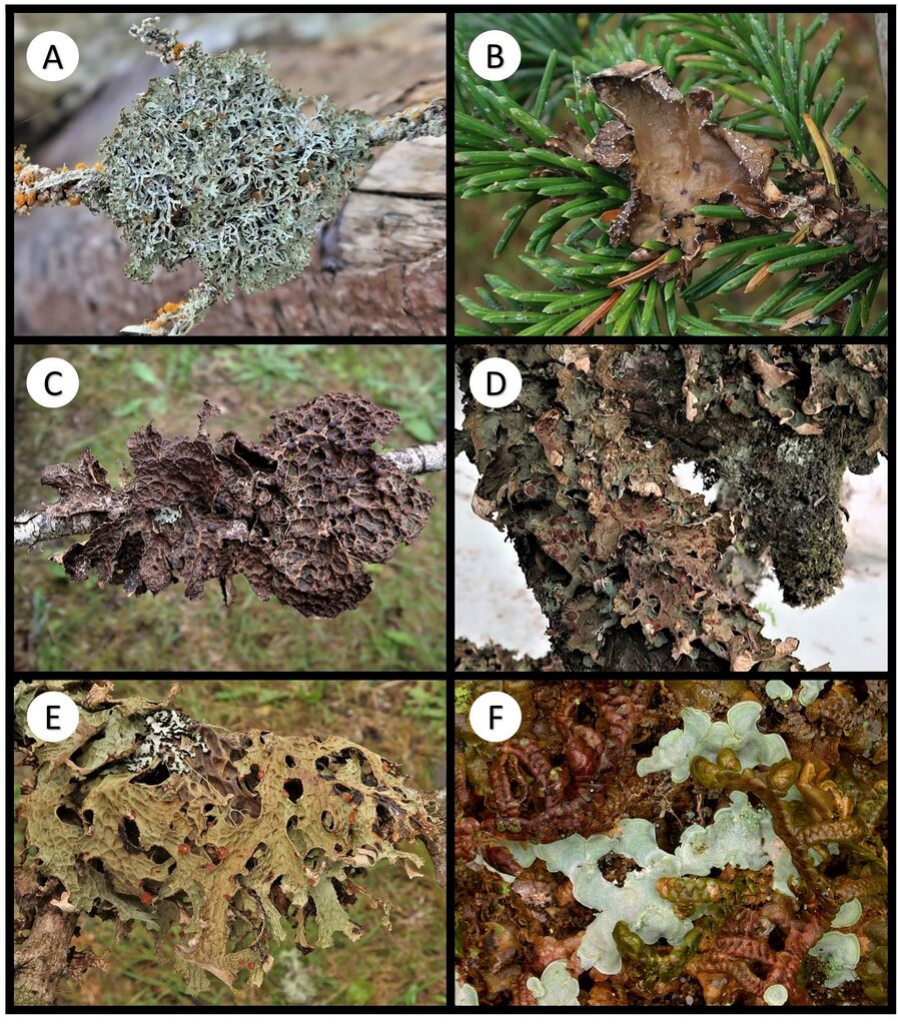
Lichens of Calvert Island. **A.** *Hypogymnialophyrea*, *McMullin 19770* (CANL). **B.** *Lobariaanomala*, *McMullin 19563* (CANL). **C.** *Lobariaanthraspis*, *McMullin 19674* (CANL). **D.** *Lobarialinita*, *McMullin 19543* (CANL). **E.** *Lobariapulmonaria*, *McMullin 19656* (CANL). **F.** *Normandinapulchella*, *McMullin 19718* (CANL).

- **Type status:** Other material. **Occurrence:** catalogNumber: BOLD CALV282-20; recordedBy: R.T. McMullin; otherCatalogNumbers: GenBank OQ843306; occurrenceID: 08C79C6E-B5D8-5605-941C-CD0979A64BCF; **Location:** locationID: XIII; decimalLatitude: 51.66854; decimalLongitude: -128.11832; **Event:** habitat: Corticolous on *Alnusrubra*; **Record Level:** institutionID: CANL; collectionID: McMullin 19597
- **Type status:** Other material. **Occurrence:** recordedBy: R.T. McMullin; occurrenceID: 4322D907-E3C1-5377-9459-175318D61082; **Location:** locationID: XVIII; decimalLatitude: 51.66476; decimalLongitude: -128.11798; **Event:** habitat: Corticolous on *Piceasitchensis*; **Record Level:** institutionID: CANL; collectionID: McMullin 19690
- **Type status:** Other material. **Occurrence:** catalogNumber: BOLD CALV284-20; recordedBy: R.T. McMullin; otherCatalogNumbers: GenBank OQ843337; occurrenceID: 52BD0688-70F7-5AD5-938A-F2EFFC93E23A; **Location:** locationID: II; decimalLatitude: 51.65285; decimalLongitude: -128.13873; **Identification:** identificationRemarks: Figure 3A; **Event:** habitat: Corticolous on *Piceasitchensis*; **Record Level:** institutionID: CANL; collectionID: McMullin 19770
- **Type status:** Other material. **Occurrence:** recordedBy: A. Simon; occurrenceID: 0C3C1AE1-4425-53FF-8747-DCBE050BFD37; **Location:** locationID: XIII; decimalLatitude: 51.66854; decimalLongitude: -128.11832; **Record Level:** institutionID: UBC; collectionID: Simon 784

#### Hypogymnia physodes

(L.) Nyl.

B0FE7B22-808F-5484-9C67-DE25A4165DE3

##### Materials

- **Type status:** Other material. **Occurrence:** recordedBy: R.T. McMullin; occurrenceID: 886AD329-0B16-506D-B529-DB78D770F20D; **Location:** locationID: XX; decimalLatitude: 51.64809; decimalLongitude: -128.14378; **Event:** habitat: Corticolous on a snag; **Record Level:** institutionID: CANL; collectionID: McMullin 19504

#### Hypogymnia vittata

(Ach.) Parrique

00DEC804-4918-593B-8AAC-D2ACEFF00BCC

##### Materials

- **Type status:** Other material. **Occurrence:** recordedBy: Schofield; occurrenceID: 3E092520-D632-5DD7-87AE-653891DC0474; **Location:** locationID: On the slopes of Mt. Buxton; **Record Level:** institutionID: UBC; collectionID: Schofield 27996a

##### Notes

Collected on Calvert Island prior to our survey, but we did not locate it.

#### Icmadophila ericetorum

(L.) Zahlbr.

AB4B83EB-45ED-58D6-B77F-B95479BEC0B4

##### Materials

- **Type status:** Other material. **Occurrence:** catalogNumber: BOLD CALV001-20; recordedBy: R.T. McMullin; otherCatalogNumbers: GenBank OQ843269; occurrenceID: 246957CE-8DCE-5FE9-924B-06CC86D11772; **Location:** locationID: XIX; decimalLatitude: 51.65065; decimalLongitude: -128.14241; **Event:** habitat: Lignicolous on a stump; **Record Level:** institutionID: CANL; collectionID: McMullin 19747
- **Type status:** Other material. **Occurrence:** catalogNumber: BOLD PHAK341-20; recordedBy: A. Simon; otherCatalogNumbers: GenBank OQ922953; occurrenceID: C63949D1-FFEF-56E7-BC89-4CBA94A13829; **Location:** locationID: XIV; decimalLatitude: 51.66797; decimalLongitude: -128.12128; **Record Level:** institutionID: UBC; collectionID: Simon 788

#### Kaernefeltia californica

(Tuck.) A. Thell & Goward

C60D60A9-7C88-568D-8EAB-691C95D978FF

##### Materials

- **Type status:** Other material. **Occurrence:** catalogNumber: BOLD CALV002-20; recordedBy: R.T. McMullin; otherCatalogNumbers: GenBank OQ843342; occurrenceID: 18853D71-CDA5-5071-B56D-5B1B464DC3F8; **Location:** locationID: XXIII; decimalLatitude: 51.64058; decimalLongitude: -128.14882; **Event:** habitat: Corticolous on a conifer snag; **Record Level:** institutionID: CANL; collectionID: McMullin 19692

#### Lecanactis abietina

(Ach.) Körb.

E21B716B-0F3B-57D6-A2BF-677E1BE155D3

##### Materials

- **Type status:** Other material. **Occurrence:** recordedBy: R.T. McMullin; occurrenceID: F0447C1F-3DDB-56E5-84ED-6F5A880C641A; **Location:** locationID: VIII; decimalLatitude: 51.61611; decimalLongitude: -127.93962; **Event:** habitat: Lignicolous on *Thujaplicata*; **Record Level:** institutionID: CANL; collectionID: McMullin 19660

#### Lecania hydrophobica

T. Sprib. & Fryday

A7FC9DDD-B764-570A-A561-B076A28932E2

##### Materials

- **Type status:** Other material. **Occurrence:** recordedBy: R.T. McMullin; occurrenceID: D438C6C5-DBB1-5EEE-80D5-2A33BF6B87AE; **Location:** locationID: V; decimalLatitude: 51.62022; decimalLongitude: -127.93070; **Identification:** identificationRemarks: TLC: atranorin, gangaleoidin, and norgangaleoidin; **Event:** habitat: Saxicolous; **Record Level:** institutionID: CANL; collectionID: McMullin 19639
- **Type status:** Other material. **Occurrence:** recordedBy: R.T. McMullin; occurrenceID: 774074D2-0B59-5D95-97D5-F7F00F4132D3; **Location:** locationID: II; decimalLatitude: 51.65285; decimalLongitude: -128.13873; **Identification:** identificationRemarks: TLC: atranorin, gangaleoidin, and norgangaleoidin; **Event:** habitat: Saxicolous; **Record Level:** institutionID: CANL; collectionID: McMullin 19774

#### Lecanora atrosulphurea

(Wahlenb.) Ach.

EB0790D0-8994-5BB2-A6CE-E1426E41F885

##### Materials

- **Type status:** Other material. **Occurrence:** recordedBy: R.T. McMullin; occurrenceID: D6E847AD-0AD5-58BB-8694-0BC0D3E827D5; **Location:** locationID: XIV; decimalLatitude: 51.66797; decimalLongitude: -128.12128; **Identification:** identificationRemarks: TLC: thiophanic and usnic acids, zeorin, and unknown fatty acids; **Event:** habitat: Saxicolous; **Record Level:** institutionID: CANL; collectionID: McMullin 19689

#### Lecanora cinereofusca

H. Magn.

B66FFF5B-62FD-5FEC-A3F6-174918CA23F6

##### Materials

- **Type status:** Other material. **Occurrence:** recordedBy: R.T. McMullin; occurrenceID: 5268648D-7229-5AAD-9DE9-6FCBDF53710F; **Location:** locationID: V; decimalLatitude: 51.62022; decimalLongitude: -127.93070; **Event:** habitat: Corticolous on *Alnusrubra*; **Record Level:** institutionID: CANL; collectionID: McMullin 19649
- **Type status:** Other material. **Occurrence:** recordedBy: A. Simon; occurrenceID: 94652EEE-634C-5FC6-AB83-0BEB89C931CD; **Location:** locationID: V; decimalLatitude: 51.62022; decimalLongitude: -127.93070; **Record Level:** institutionID: UBC; collectionID: Simon 827

##### Notes

Epihymenium with pannarin.

#### Lecanora poliophaea

(Wahlenb.) Ach.

72DA8327-96F3-53FA-87EA-7E44DEA67047

##### Materials

- **Type status:** Other material. **Occurrence:** catalogNumber: BOLD PHAK379-20; recordedBy: A. Simon; otherCatalogNumbers: GenBank OQ922965; occurrenceID: 252C82F0-DFA7-5364-A2F2-A43C395C0E41; **Location:** locationID: V; decimalLatitude: 51.62022; decimalLongitude: -127.93070; **Record Level:** institutionID: UBC; collectionID: Simon 820

#### Lecanora symmicta

(Ach.) Ach.

6FEB8B10-BF98-5BDC-B061-6A777908694A

##### Materials

- **Type status:** Other material. **Occurrence:** catalogNumber: BOLD CALV006-20; recordedBy: R.T. McMullin; otherCatalogNumbers: GenBank OQ843220; occurrenceID: 85B136E9-8CC9-54E3-9A26-81B8F8ED31E6; **Location:** locationID: III; decimalLatitude: 51.65486; decimalLongitude: -128.13907; **Event:** habitat: Lignicolous on a log; **Record Level:** institutionID: CANL; collectionID: McMullin 19752

#### Lecanora xylophila

Hue

31A5679F-CCF4-5C2C-9159-582CDE7DD222

##### Materials

- **Type status:** Other material. **Occurrence:** recordedBy: R.T. McMullin; occurrenceID: C1273A93-3CBB-54A8-B0F9-CD6B590EF992; **Location:** locationID: V; decimalLatitude: 51.62022; decimalLongitude: -127.93070; **Event:** habitat: Saxicolous; **Record Level:** institutionID: CANL; collectionID: McMullin 19627
- **Type status:** Other material. **Occurrence:** recordedBy: R.T. McMullin; occurrenceID: 6BB7FDC4-1F5B-581A-A9B8-C1E932EBA357; **Location:** locationID: XII; decimalLatitude: 51.66040; decimalLongitude: -128.11688; **Event:** habitat: Lignicolous on a log; **Record Level:** institutionID: CANL; collectionID: McMullin 19646
- **Type status:** Other material. **Occurrence:** recordedBy: R.T. McMullin; occurrenceID: 484C9AD5-1380-52EB-9BFA-71C2D8D1E034; **Location:** locationID: III; decimalLatitude: 51.65486; decimalLongitude: -128.13907; **Event:** habitat: Lignicolous on a log; **Record Level:** institutionID: CANL; collectionID: McMullin 19777
- **Type status:** Other material. **Occurrence:** catalogNumber: BOLD PHAK338-20; recordedBy: A. Simon; otherCatalogNumbers: GenBank OQ922959; occurrenceID: A38933B7-124E-5427-A012-E53CC03BD08C; **Location:** locationID: XXXI; decimalLatitude: 51.64512; decimalLongitude: -128.09463; **Record Level:** institutionID: UBC; collectionID: Simon 768

#### Lepra amara

(Ach.) Hafellner

1D85FE21-22E8-5F97-B8B7-457BF9DEFF34

##### Materials

- **Type status:** Other material. **Occurrence:** recordedBy: R.T. McMullin; occurrenceID: 2BF83F69-C0FB-5202-8A5B-746BEF8F5203; **Location:** locationID: XXI; decimalLatitude: 51.64221; decimalLongitude: -128.15085; **Event:** habitat: Corticolous on *Alnusrubra*; **Record Level:** institutionID: CANL; collectionID: McMullin 19526
- **Type status:** Other material. **Occurrence:** recordedBy: R.T. McMullin; occurrenceID: F5081B85-8963-50F3-8855-F36E4A702271; **Location:** locationID: V; decimalLatitude: 51.62022; decimalLongitude: -127.93070; **Event:** habitat: Corticolous on a snag; **Record Level:** institutionID: CANL; collectionID: McMullin 19682
- **Type status:** Other material. **Occurrence:** recordedBy: R.T. McMullin; occurrenceID: 294F1FE1-6B07-5433-B58F-6C678AD2F323; **Location:** locationID: XII; decimalLatitude: 51.66040; decimalLongitude: -128.11688; **Event:** habitat: Corticolous on a deciduous tree; **Record Level:** institutionID: CANL; collectionID: McMullin 19879
- **Type status:** Other material. **Occurrence:** recordedBy: A. Simon; occurrenceID: 3A269BCE-81CB-526C-8DA5-B3957B238B34; **Location:** locationID: XXI; decimalLatitude: 51.64221; decimalLongitude: -128.15085; **Record Level:** institutionID: UBC; collectionID: Simon 813

##### Notes

Soralia with picrolichenic acid.

#### Lepra borealis

(Erichsen) I. Schmitt, B.P. Hodk. & Lumbsch

56932938-50C5-584A-A6F5-8182299046AD

##### Materials

- **Type status:** Other material. **Occurrence:** recordedBy: R.T. McMullin; occurrenceID: B9B9D907-7F70-5080-87E8-F4008BF5AB08; **Location:** locationID: V; decimalLatitude: 51.62022; decimalLongitude: -127.93070; **Identification:** identificationRemarks: TLC: fumarprotocetraric and protocetraric acids; **Event:** habitat: Lignicolous; **Record Level:** institutionID: CANL; collectionID: McMullin 19647
- **Type status:** Other material. **Occurrence:** recordedBy: R.T. McMullin; occurrenceID: 9D4E1B91-AD66-53B9-A9B6-0B4C0714F3D4; **Location:** locationID: V; decimalLatitude: 51.62022; decimalLongitude: -127.93070; **Identification:** identificationRemarks: TLC: fumarprotocetraric and protocetraric acids; **Event:** habitat: Corticolous on *Alnusrubra*; **Record Level:** institutionID: CANL; collectionID: McMullin 19704

#### Lepra subvelata

(G.K. Merr.) T. Sprib.

89975894-8EDF-59A5-8525-2B38DCF24DE1

##### Materials

- **Type status:** Other material. **Occurrence:** recordedBy: R.T. McMullin; occurrenceID: 0B89078F-E4DC-5D83-BDE5-B92931C0BBA4; **Location:** locationID: V; decimalLatitude: 51.62022; decimalLongitude: -127.93070; **Identification:** identificationRemarks: TLC: nephrosteranic acid; **Event:** habitat: Lignicolous; **Record Level:** institutionID: CANL; collectionID: McMullin 19624
- **Type status:** Other material. **Occurrence:** recordedBy: R.T. McMullin; occurrenceID: 13ACBEDC-46E5-5E4A-A2A5-567B42B27EA9; **Location:** locationID: XII; decimalLatitude: 51.66040; decimalLongitude: -128.11688; **Identification:** identificationRemarks: TLC: nephrosteranic acid; **Event:** habitat: Lignicolous on a snag; **Record Level:** institutionID: CANL; collectionID: McMullin 19630
- **Type status:** Other material. **Occurrence:** recordedBy: R.T. McMullin; occurrenceID: 7F653207-D9F6-539E-BDB6-2C5A7B0C6B31; **Location:** locationID: XII; decimalLatitude: 51.66040; decimalLongitude: -128.11688; **Identification:** identificationRemarks: TLC: nephrosteranic acid; **Event:** habitat: Corticolous on Pinuscontortassp.contorta; **Record Level:** institutionID: CANL; collectionID: McMullin 19645
- **Type status:** Other material. **Occurrence:** recordedBy: R.T. McMullin; occurrenceID: D7BA0327-FD5D-5A2E-B500-2C3CEA88F2DE; **Location:** locationID: XIX; decimalLatitude: 51.65065; decimalLongitude: -128.14241; **Identification:** identificationRemarks: TLC: nephrosteranic acid; **Event:** habitat: Lignicolous on a snag; **Record Level:** institutionID: CANL; collectionID: McMullin 19851
- **Type status:** Other material. **Occurrence:** catalogNumber: BOLD PHAK359-20; recordedBy: A. Simon; otherCatalogNumbers: GenBank OQ922939; occurrenceID: 66E2D24A-DE18-5A5C-8E6E-7675A8529E91; **Location:** locationID: V; decimalLatitude: 51.62022; decimalLongitude: -127.93070; **Record Level:** institutionID: UBC; collectionID: Simon 835
- **Type status:** Other material. **Occurrence:** recordedBy: A. Simon; occurrenceID: 6998A5B5-2D43-578A-BB3A-3C2EED88A4E0; **Location:** locationID: V; decimalLatitude: 51.62022; decimalLongitude: -127.93070; **Record Level:** institutionID: UBC; collectionID: Simon 836

##### Notes

Spribille et al. (2020) point out the distinctions of this rarely reported, but probably common coastal species from *Lepraophthalmiza* (Nyl.) Hafellner.

#### Lepraria brodoi

Lendemer & Tønsberg

5FBC674D-14B3-5949-9ED2-973262CD24E8

##### Materials

- **Type status:** Other material. **Occurrence:** recordedBy: R.T. McMullin; occurrenceID: B4494135-B77E-5503-8979-42FD73F4B0D9; **Location:** locationID: XIX; decimalLatitude: 51.65065; decimalLongitude: -128.14241; **Identification:** identificationRemarks: TLC: alectorialic acid was the major substance detected, minor substances were not always clear, and no psoromic acid was detected.; **Event:** habitat: Corticolous on *Tsuga*; **Record Level:** institutionID: CANL; collectionID: McMullin 19805
- **Type status:** Other material. **Occurrence:** recordedBy: R.T. McMullin; occurrenceID: 294822CC-7C5A-57B6-97D2-359E83AF6FEF; **Location:** locationID: XXI; decimalLatitude: 51.64221; decimalLongitude: -128.15085; **Identification:** identificationRemarks: TLC: alectorialic acid was the major substance detected, minor substances were not always clear, and no psoromic acid was detected.; **Event:** habitat: Bryicolous on *Tsuga*; **Record Level:** institutionID: CANL; collectionID: McMullin 19812
- **Type status:** Other material. **Occurrence:** recordedBy: R.T. McMullin; occurrenceID: F2C9AC47-DD1B-5907-B745-EAEBE17A3EDB; **Location:** locationID: XXIV; decimalLatitude: 51.65271; decimalLongitude: -128.12951; **Identification:** identificationRemarks: TLC: alectorialic acid was the major substance detected, minor substances were not always clear, and no psoromic acid was detected.; **Event:** habitat: Corticolous on a conifer snag; **Record Level:** institutionID: CANL; collectionID: McMullin 19813
- **Type status:** Other material. **Occurrence:** recordedBy: R.T. McMullin; occurrenceID: C0915F00-FC7F-5E0F-9BC2-1354B6A1E964; **Location:** locationID: VII; decimalLatitude: 51.61615; decimalLongitude: -127.93851; **Identification:** identificationRemarks: TLC: alectorialic acid was the major substance detected, minor substances were not always clear, and no psoromic acid was detected.; **Event:** habitat: Corticolous on the base of *Tsuga*; **Record Level:** institutionID: CANL; collectionID: McMullin 19817
- **Type status:** Other material. **Occurrence:** recordedBy: R.T. McMullin; occurrenceID: 2141B7F9-9B75-5FAB-9911-A4A7C9294D8D; **Location:** locationID: X; decimalLatitude: 51.61977; decimalLongitude: -127.93245; **Identification:** identificationRemarks: TLC: alectorialic acid was the major substance detected, minor substances were not always clear, and no psoromic acid was detected.; **Event:** habitat: Saxicolous on a cliff; **Record Level:** institutionID: CANL; collectionID: McMullin 19818
- **Type status:** Other material. **Occurrence:** catalogNumber: BOLD CALV176-20; recordedBy: R.T. McMullin; otherCatalogNumbers: GenBank OQ843285; occurrenceID: D35CB757-6DD2-5A19-B628-5A5D84D557DA; **Location:** locationID: III; decimalLatitude: 51.65486; decimalLongitude: -128.13907; **Identification:** identificationRemarks: TLC: alectorialic acid was the major substance detected, minor substances were not always clear, and no psoromic acid was detected.; **Event:** habitat: Terricolous; **Record Level:** institutionID: CANL; collectionID: McMullin 19820
- **Type status:** Other material. **Occurrence:** recordedBy: R.T. McMullin; occurrenceID: 27C44044-4C67-5B09-A4C6-97816514DA5B; **Location:** locationID: VII; decimalLatitude: 51.61615; decimalLongitude: -127.93851; **Identification:** identificationRemarks: TLC: alectorialic acid was the major substance detected, minor substances were not always clear, and no psoromic acid was detected.; **Event:** habitat: Corticolous on the base of *Tsuga*; **Record Level:** institutionID: CANL; collectionID: McMullin 19821

#### Lepraria finkii

(de Lesl.) R.C. Harris

1E693C78-59D5-5FC0-8D37-F2510EA259B9

##### Materials

- **Type status:** Other material. **Occurrence:** catalogNumber: BOLD CALV018-20; recordedBy: R.T. McMullin; otherCatalogNumbers: GenBank OQ843351; occurrenceID: 25B47FCF-85F9-5A98-A85D-690C749A46CF; **Location:** locationID: V; decimalLatitude: 51.62022; decimalLongitude: -127.93070; **Identification:** identificationRemarks: TLC: atranorin, stictic acid, and zeorin; **Event:** habitat: Terricolous; **Record Level:** institutionID: CANL; collectionID: McMullin 19824
- **Type status:** Other material. **Occurrence:** catalogNumber: BOLD CALV019-20; recordedBy: R.T. McMullin; otherCatalogNumbers: GenBank OQ843341; occurrenceID: 40E6020D-4C92-5CB2-9BA3-9B85B216BC45; **Location:** locationID: V; decimalLatitude: 51.62022; decimalLongitude: -127.93070; **Identification:** identificationRemarks: TLC: atranorin, stictic acid, and zeorin; **Event:** habitat: Terricolous; **Record Level:** institutionID: CANL; collectionID: McMullin 19825

#### Lepraria jackii

Tønsberg

08C5D611-DBEF-575D-8900-7EAE49FFAA2A

##### Materials

- **Type status:** Other material. **Occurrence:** catalogNumber: BOLD CALV020-20; recordedBy: R.T. McMullin; otherCatalogNumbers: GenBank OQ843245; occurrenceID: 790AC607-8E2F-5E1F-BBA9-FBC4BBAB94B9; **Location:** locationID: III; decimalLatitude: 51.65486; decimalLongitude: -128.13907; **Identification:** identificationRemarks: TLC: atranorin, jackinic acid, and roccellic/angardianic acid; **Event:** habitat: Terricolous; **Record Level:** institutionID: CANL; collectionID: McMullin 19834

#### Lepraria membranacea

(Dicks.) Vain.

49E3E914-498C-5591-BDC4-F60857A3D995

##### Materials

- **Type status:** Other material. **Occurrence:** catalogNumber: BOLD CALV184-20; recordedBy: R.T. McMullin; otherCatalogNumbers: GenBank OQ843312; occurrenceID: 2144AE05-996E-5AF0-98F1-841618D85BA6; **Location:** locationID: VII; decimalLatitude: 51.61615; decimalLongitude: -127.93851; **Identification:** identificationRemarks: TLC: unknown substances at RF 1 & 7; **Event:** habitat: Corticolous on a conifer snag; **Record Level:** institutionID: CANL; collectionID: McMullin 19815

#### Lepraria neglecta

(Nyl.) Erichsen

1DC8873E-6E56-5E3A-AF84-33C8CF53C8EF

##### Materials

- **Type status:** Other material. **Occurrence:** recordedBy: R.T. McMullin; occurrenceID: 5A4FF5B8-F5AF-5240-BE15-FD9480073496; **Location:** locationID: XV; decimalLatitude: 51.65616; decimalLongitude: -128.14052; **Identification:** identificationRemarks: TLC: fumarprotocetraric, and roccellic/angardianic acids; no atranorin; **Event:** habitat: Saxicolous and terricolous; **Record Level:** institutionID: CANL; collectionID: McMullin 19816

#### Lepraria nivalis

J.R. Laundon

C28288F5-3442-5AFA-A09D-6FF53FA5D445

##### Materials

- **Type status:** Other material. **Occurrence:** catalogNumber: BOLD CALV023-20; recordedBy: R.T. McMullin; otherCatalogNumbers: GenBank OQ843321; occurrenceID: 8A886F9D-29BE-5A0E-90FA-8FC09B5E48D6; **Location:** locationID: VIII; decimalLatitude: 51.61611; decimalLongitude: -127.93962; **Identification:** identificationRemarks: TLC: atranorin and protocetraric acid; **Event:** habitat: Corticolous on *Thujaplicata*; **Record Level:** institutionID: CANL; collectionID: McMullin 19827

#### Lepraria pacifica

Lendemer

40015E61-1AB8-5F3B-A39D-941FFF507A23

##### Materials

- **Type status:** Other material. **Occurrence:** recordedBy: R.T. McMullin; occurrenceID: 8607B28E-E3B6-5FA0-9422-502DBAC3AFA5; **Location:** locationID: III; decimalLatitude: 51.65486; decimalLongitude: -128.13907; **Identification:** identificationRemarks: TLC: divaricatic acid and zeorin; **Event:** habitat: Corticolous on *Alnusrubra*; **Record Level:** institutionID: CANL; collectionID: McMullin 19811
- **Type status:** Other material. **Occurrence:** recordedBy: R.T. McMullin; occurrenceID: 1FCCC5C3-1EE5-50F7-AB34-00A02F2588A5; **Location:** locationID: XIX; decimalLatitude: 51.65065; decimalLongitude: -128.14241; **Identification:** identificationRemarks: TLC: divaricatic acid and zeorin; **Event:** habitat: Corticolous on a stump; **Record Level:** institutionID: CANL; collectionID: McMullin 19814
- **Type status:** Other material. **Occurrence:** catalogNumber: BOLD CALV025-20; recordedBy: R.T. McMullin; otherCatalogNumbers: GenBank OQ843360; occurrenceID: 8DB40252-9866-5C9D-B63C-10850B548DCA; **Location:** locationID: IV; decimalLatitude: 51.65514; decimalLongitude: -128.13243; **Identification:** identificationRemarks: TLC: divaricatic acid and zeorin; **Event:** habitat: Corticolous on *Thujaplicata*; **Record Level:** institutionID: CANL; collectionID: McMullin 19823
- **Type status:** Other material. **Occurrence:** recordedBy: R.T. McMullin; occurrenceID: FF349D63-CCA2-548C-A161-F56E8AEA8664; **Location:** locationID: V; decimalLatitude: 51.62022; decimalLongitude: -127.93070; **Identification:** identificationRemarks: TLC: divaricatic acid and zeorin; **Event:** habitat: Corticolous on *Thujaplicata*; **Record Level:** institutionID: CANL; collectionID: McMullin 19826

#### Lepraria torii

Pérez-Ortega & T. Sprib.

DB7F3CCC-0220-512D-809B-26B674176B6A

##### Materials

- **Type status:** Other material. **Occurrence:** catalogNumber: BOLD CALV026-20; recordedBy: R.T. McMullin; otherCatalogNumbers: GenBank OQ843277; occurrenceID: 94BBDAB2-2DFC-586A-97FD-10B0AAF10DF2; **Location:** locationID: XIX; decimalLatitude: 51.65065; decimalLongitude: -128.14241; **Identification:** identificationRemarks: TLC: fumarprotocetraric and roccellic/angardianic acids; **Event:** habitat: Terricolous; **Record Level:** institutionID: CANL; collectionID: McMullin 19810
- **Type status:** Other material. **Occurrence:** catalogNumber: BOLD CALV027-20; recordedBy: R.T. McMullin; otherCatalogNumbers: GenBank OQ843199; occurrenceID: 7EE14A08-B3A5-5597-AD9C-11C64736327E; **Location:** locationID: VII; decimalLatitude: 51.61615; decimalLongitude: -127.93851; **Identification:** identificationRemarks: TLC: fumarprotocetraric and roccellic/angardianic acids; **Event:** habitat: Corticolous on the base of a snag; **Record Level:** institutionID: CANL; collectionID: McMullin 19835
- **Type status:** Other material. **Occurrence:** catalogNumber: BOLD CALV028-20; recordedBy: R.T. McMullin; otherCatalogNumbers: GenBank OQ843237; occurrenceID: 6236897F-5D53-5A96-87B3-9B0BB31D4986; **Location:** locationID: IV; decimalLatitude: 51.65514; decimalLongitude: -128.13243; **Identification:** identificationRemarks: TLC: fumarprotocetraric and roccellic/angardianic acids; **Event:** habitat: Corticolous on a conifer snag; **Record Level:** institutionID: CANL; collectionID: McMullin 19836

#### Lobaria anomala

(Brodo & Ahti) T. Sprib. & McCune

151925FB-E19C-5EC4-A179-8473525BF967

##### Materials

- **Type status:** Other material. **Occurrence:** catalogNumber: BOLD CALV032-20; recordedBy: R.T. McMullin; otherCatalogNumbers: GenBank OQ843253; occurrenceID: 38D827D3-72C3-59E5-A63F-D0BE94A53880; **Location:** locationID: XXI; decimalLatitude: 51.64221; decimalLongitude: -128.15085; **Event:** habitat: Corticolous on *Alnusrubra*; **Record Level:** institutionID: CANL; collectionID: McMullin 19541
- **Type status:** Other material. **Occurrence:** catalogNumber: BOLD CALV033-20; recordedBy: R.T. McMullin; otherCatalogNumbers: GenBank OQ843232; occurrenceID: D91DA74C-22D7-58F8-8B7C-7793AE5F9ECA; **Location:** locationID: XVII; decimalLatitude: 51.66213; decimalLongitude: -128.14492; **Identification:** identificationRemarks: Figure 3B; **Event:** habitat: Corticolous on *Piceasitchensis*; **Record Level:** institutionID: CANL; collectionID: McMullin 19563
- **Type status:** Other material. **Occurrence:** catalogNumber: BOLD CALV034-20; recordedBy: R.T. McMullin; otherCatalogNumbers: GenBank OQ843294; occurrenceID: 56CC278D-355A-571E-8512-CB9A953084BA; **Location:** locationID: XIII; decimalLatitude: 51.66854; decimalLongitude: -128.11832; **Event:** habitat: Corticolous on *Alnusrubra*; **Record Level:** institutionID: CANL; collectionID: McMullin 19695

#### Lobaria anthraspis

(Ach.) T. Sprib. & McCune

6CF9652F-927D-5016-9AD8-B53C5D5925F4

##### Materials

- **Type status:** Other material. **Occurrence:** catalogNumber: BOLD CALV035-20; recordedBy: R.T. McMullin; otherCatalogNumbers: GenBank OQ843259; occurrenceID: B43038FD-119D-5D65-970F-DB1EE8537631; **Location:** locationID: V; decimalLatitude: 51.62022; decimalLongitude: -127.93070; **Event:** habitat: Corticolous; **Record Level:** institutionID: CANL; collectionID: McMullin 19604
- **Type status:** Other material. **Occurrence:** catalogNumber: BOLD CALV036-20; recordedBy: R.T. McMullin; otherCatalogNumbers: GenBank OQ843216; occurrenceID: 3A65CE59-C28E-5CA2-A764-5D7E20435B5B; **Location:** locationID: XVII; decimalLatitude: 51.66213; decimalLongitude: -128.14492; **Event:** habitat: Corticolous on *Piceasitchensis*; **Record Level:** institutionID: CANL; collectionID: McMullin 19607
- **Type status:** Other material. **Occurrence:** catalogNumber: BOLD CALV037-20; recordedBy: R.T. McMullin; otherCatalogNumbers: GenBank OQ843206; occurrenceID: 96BA659A-D673-553D-B175-000A159C9862; **Location:** locationID: XIII; decimalLatitude: 51.66854; decimalLongitude: -128.11832; **Identification:** identificationRemarks: Figure 3C; **Event:** habitat: Corticolous on *Piceasitchensis*; **Record Level:** institutionID: CANL; collectionID: McMullin 19674
- **Type status:** Other material. **Occurrence:** recordedBy: R.T. McMullin; occurrenceID: 64710ACE-9CB9-5D6C-A82E-2EDF657750DC; **Location:** locationID: III; decimalLatitude: 51.65486; decimalLongitude: -128.13907; **Event:** habitat: Corticolous on *Piceasitchensis*; **Record Level:** institutionID: CANL; collectionID: McMullin 19778
- **Type status:** Other material. **Occurrence:** recordedBy: R.T. McMullin; occurrenceID: 286F4E79-68D1-5EA1-9269-487BF7D223FB; **Location:** locationID: XVII; decimalLatitude: 51.66213; decimalLongitude: -128.14492; **Event:** habitat: Corticolous on *Alnusrubra*; **Record Level:** institutionID: CANL; collectionID: McMullin 19867
- **Type status:** Other material. **Occurrence:** recordedBy: A. Simon; occurrenceID: 154090BA-9AE0-5116-A785-54CD5B4D391B; **Location:** locationID: XIII; decimalLatitude: 51.66854; decimalLongitude: -128.11832; **Record Level:** institutionID: UBC; collectionID: Simon 789
- **Type status:** Other material. **Occurrence:** catalogNumber: BOLD PHAK328-20; recordedBy: A. Simon; otherCatalogNumbers: GenBank OQ922955; occurrenceID: B1F87AFF-280F-59C1-887B-4B7AFEEEBBF4; **Location:** locationID: XIII; decimalLatitude: 51.66854; decimalLongitude: -128.11832; **Record Level:** institutionID: UBC; collectionID: Simon 790

#### Lobaria linita

(Ach.) Rabenh.

7BC65324-1E10-5430-87B1-4E6CC133EE98

##### Materials

- **Type status:** Other material. **Occurrence:** catalogNumber: BOLD CALV038-20; recordedBy: R.T. McMullin; otherCatalogNumbers: GenBank OQ843340; occurrenceID: 1EC64B98-6210-55DC-9278-2E28ABD1E5C6; **Location:** locationID: XXI; decimalLatitude: 51.64221; decimalLongitude: -128.15085; **Identification:** identificationRemarks: Figure 3D; **Event:** habitat: Corticolous on *Tsuga*; **Record Level:** institutionID: CANL; collectionID: McMullin 19543

#### Lobaria oregana

(Tuck.) Müll. Arg.

F8C60B76-477C-586B-A7CC-644BE97BEE09

##### Materials

- **Type status:** Other material. **Occurrence:** recordedBy: R.T. McMullin; occurrenceID: D4C7ABE9-D2FD-5E42-A376-0F1873278687; **Location:** locationID: X; decimalLatitude: 51.61977; decimalLongitude: -127.93245; **Event:** habitat: Corticolous on *Tsuga*; **Record Level:** institutionID: CANL; collectionID: McMullin 19634
- **Type status:** Other material. **Occurrence:** recordedBy: R.T. McMullin; occurrenceID: 92388F94-00ED-500A-AA1F-8EA7861BA5B0; **Location:** locationID: XIX; decimalLatitude: 51.65065; decimalLongitude: -128.14241; **Event:** habitat: Corticolous on *Tsuga*; **Record Level:** institutionID: CANL; collectionID: McMullin 19749
- **Type status:** Other material. **Occurrence:** catalogNumber: BOLD PHAK288-20; recordedBy: A. Simon; otherCatalogNumbers: GenBank OQ922984; occurrenceID: 4CFCD705-69E3-55BA-95BF-38ED4852CA44; **Location:** locationID: XXVIII; decimalLatitude: 51.66425; decimalLongitude: -128.12706; **Record Level:** institutionID: UBC; collectionID: Simon 754

#### Lobaria pulmonaria

(L.) Hoffm.

2658C5B9-2BDB-52F0-98AD-CB6ED27E6129

##### Materials

- **Type status:** Other material. **Occurrence:** recordedBy: R.T. McMullin; occurrenceID: 5BC8ED45-8D61-5AFE-8B9C-BC1F62B4CA18; **Location:** locationID: XIII; decimalLatitude: 51.66854; decimalLongitude: -128.11832; **Identification:** identificationRemarks: Figure 3E; **Event:** habitat: Corticolous on *Piceasitchensis*; **Record Level:** institutionID: CANL; collectionID: McMullin 19656
- **Type status:** Other material. **Occurrence:** catalogNumber: BOLD CALV043-20; recordedBy: R.T. McMullin; otherCatalogNumbers: GenBank OQ843228; occurrenceID: 7118D5C4-1921-508B-8E3D-60662988997A; **Location:** locationID: III; decimalLatitude: 51.65486; decimalLongitude: -128.13907; **Event:** habitat: Corticolous on *Piceasitchensis*; **Record Level:** institutionID: CANL; collectionID: McMullin 19726
- **Type status:** Other material. **Occurrence:** catalogNumber: BOLD PHAK302-20; recordedBy: A. Simon; otherCatalogNumbers: GenBank OQ922949; occurrenceID: 5EB5CE7B-8B34-50B1-8751-8B0430416F52; **Location:** locationID: I; decimalLatitude: 51.65501; decimalLongitude: -128.13799; **Record Level:** institutionID: UBC; collectionID: Simon 808
- **Type status:** Other material. **Occurrence:** recordedBy: A. Simon; occurrenceID: 4E05BE43-AD1C-54B0-A601-421FDEB874C9; **Location:** locationID: XXIII; decimalLatitude: 51.64058; decimalLongitude: -128.14882; **Record Level:** institutionID: UBC; collectionID: Simon 847

#### Lobarina scrobiculata

(Scop.) Nyl. ex Cromb.

359B96C8-00F3-5159-A014-29F7168DB6E0

##### Materials

- **Type status:** Other material. **Occurrence:** recordedBy: R.T. McMullin; occurrenceID: B4817CCD-976E-5D81-B252-850FC14CE670; **Location:** locationID: XXI; decimalLatitude: 51.64221; decimalLongitude: -128.15085; **Event:** habitat: Corticolous on *Alnusrubra*; **Record Level:** institutionID: CANL; collectionID: McMullin 19542
- **Type status:** Other material. **Occurrence:** catalogNumber: BOLD PHAK336-20; recordedBy: A. Simon; otherCatalogNumbers: GenBank OQ922970; occurrenceID: B7DB147F-FD70-5F53-A280-D26768DD84C2; **Location:** locationID: XXI; decimalLatitude: 51.64221; decimalLongitude: -128.15085; **Record Level:** institutionID: UBC; collectionID: Simon 809

#### Loxosporopsis corallifera

Brodo, Henssen & Imshaug

B9722035-C8DD-533B-BB65-C7E8AB9D8E17

##### Materials

- **Type status:** Other material. **Occurrence:** recordedBy: R.T. McMullin; occurrenceID: FC98A58A-DA94-5795-9A58-8754D2ED0ACE; **Location:** locationID: VIII; decimalLatitude: 51.61611; decimalLongitude: -127.93962; **Event:** habitat: Corticolous on *Thujaplicata*; **Record Level:** institutionID: CANL; collectionID: McMullin 19661
- **Type status:** Other material. **Occurrence:** catalogNumber: BOLD CALV046-20; recordedBy: R.T. McMullin; otherCatalogNumbers: GenBank OQ843272; occurrenceID: B12E5C79-C692-54AB-835D-77805E36B7C9; **Location:** locationID: IX; decimalLatitude: 51.61638; decimalLongitude: -127.94035; **Event:** habitat: Corticolous on conifer snag; **Record Level:** institutionID: CANL; collectionID: McMullin 19676
- **Type status:** Other material. **Occurrence:** catalogNumber: BOLD CALV045-20; recordedBy: R.T. McMullin; otherCatalogNumbers: GenBank OQ843204; occurrenceID: 815CB21A-4D72-56E8-929F-DF6EEE12FDD2; **Location:** locationID: XXVI; decimalLatitude: 51.65466; decimalLongitude: -128.13051; **Event:** habitat: Corticolous on *Piceasitchensis*; **Record Level:** institutionID: CANL; collectionID: McMullin 19780
- **Type status:** Other material. **Occurrence:** recordedBy: R.T. McMullin; occurrenceID: F65A1752-7F0E-5447-B3DB-01E849E76535; **Location:** locationID: III; decimalLatitude: 51.65486; decimalLongitude: -128.13907; **Event:** habitat: Corticolous on *Thujaplicata*; **Record Level:** institutionID: CANL; collectionID: McMullin 19808
- **Type status:** Other material. **Occurrence:** catalogNumber: BOLD CALV147-20; recordedBy: R.T. McMullin; otherCatalogNumbers: GenBank OQ843267; occurrenceID: 7B8C1F9F-AF81-5614-B915-63E47CB591B1; **Location:** locationID: XVIII; decimalLatitude: 51.66476; decimalLongitude: -128.11798; **Event:** habitat: Lignicolous; **Record Level:** institutionID: CANL; collectionID: McMullin 19861

#### Melanelia hepatizon

(Ach.) A. Thell

073FD578-2DF9-5571-9EBC-878C5608EEF8

##### Materials

- **Type status:** Other material. **Occurrence:** catalogNumber: BOLD CALV047-20; recordedBy: R.T. McMullin; otherCatalogNumbers: GenBank OQ843262; occurrenceID: E8709658-CF0B-589F-BC26-914A9281CBC0; **Location:** locationID: XX; decimalLatitude: 51.64809; decimalLongitude: -128.14378; **Event:** habitat: Saxicolous on a rock; **Record Level:** institutionID: CANL; collectionID: McMullin 19503

#### Menegazzia terebrata

(Hoffm.) A. Massal.

4A086D43-9131-551B-B919-3A22EB5C401A

##### Materials

- **Type status:** Other material. **Occurrence:** recordedBy: A. Simon; occurrenceID: 83613D2F-273D-557C-8A01-019D1CE9E8C9; **Location:** locationID: XII; decimalLatitude: 51.66040; decimalLongitude: -128.11688; **Record Level:** institutionID: UBC; collectionID: Simon 791

#### Micarea micrococca

(Körb.) Gams ex Coppins

CD9A8646-ED57-58ED-9B06-82903EC401A8

##### Materials

- **Type status:** Other material. **Occurrence:** recordedBy: R.T. McMullin; occurrenceID: A9E62B9B-8B7B-54D5-95A3-AB7F8C0DE6C2; **Location:** locationID: II; decimalLatitude: 51.65285; decimalLongitude: -128.13873; **Identification:** identificationRemarks: TLC: methoxymicareic acid; **Event:** habitat: Lignicolous; **Record Level:** institutionID: CANL; collectionID: McMullin 19782

#### Micarea prasina

Fr.

98723A0C-6722-595F-ADA5-11BF1EABF166

##### Materials

- **Type status:** Other material. **Occurrence:** recordedBy: R.T. McMullin; occurrenceID: F01F62FB-296C-5409-8213-B2B9C28E226C; **Location:** locationID: XIX; decimalLatitude: 51.65065; decimalLongitude: -128.14241; **Identification:** identificationRemarks: TLC: micareic acid; **Event:** habitat: Corticolous on a snag; **Record Level:** institutionID: CANL; collectionID: McMullin 19789

#### Microcalicium disseminatum

(Ach.) Vain.

09052F00-8FC1-540D-BC96-28C686233058

##### Materials

- **Type status:** Other material. **Occurrence:** catalogNumber: BOLD CALV174-20; recordedBy: R.T. McMullin; otherCatalogNumbers: GenBank OQ843254; occurrenceID: E6B6193F-2F15-5381-8307-0981A2B08246; **Location:** locationID: VIII; decimalLatitude: 51.61611; decimalLongitude: -127.93962; **Event:** habitat: Lignicolous on a snag; **Record Level:** institutionID: CANL; collectionID: McMullin 19617
- **Type status:** Other material. **Occurrence:** recordedBy: R.T. McMullin; occurrenceID: DDA35AAD-DDBD-58E1-88D5-8C06F889E11A; **Location:** locationID: VIII; decimalLatitude: 51.61611; decimalLongitude: -127.93962; **Event:** habitat: Lignicolous on a snag; **Record Level:** institutionID: CANL; collectionID: McMullin 19653
- **Type status:** Other material. **Occurrence:** catalogNumber: BOLD CALV172-20; recordedBy: R.T. McMullin; otherCatalogNumbers: GenBank OQ843248; occurrenceID: D14440A9-A9AC-58D0-938A-AEC85B38A06E; **Location:** locationID: VIII; decimalLatitude: 51.61611; decimalLongitude: -127.93962; **Event:** habitat: Lignicolous on a snag; **Record Level:** institutionID: CANL; collectionID: McMullin 19856

##### Notes

Non-lichenised fungus.

#### Mycoblastus affinis

(Schaer.) T. Schauer

DD377583-0CAF-51B6-9AA3-E1388C48AB1F

##### Materials

- **Type status:** Other material. **Occurrence:** catalogNumber: BOLD CALV050-20; recordedBy: R.T. McMullin; otherCatalogNumbers: GenBank OQ843224; occurrenceID: 11EEF2A3-FBF5-5BE6-874C-1E0CB5F44B29; **Location:** locationID: V; decimalLatitude: 51.62022; decimalLongitude: -127.93070; **Identification:** identificationRemarks: TLC: atranorin and planaic acid; **Event:** habitat: Corticolous on *Piceasitchensis*; **Record Level:** institutionID: CANL; collectionID: McMullin 19573
- **Type status:** Other material. **Occurrence:** recordedBy: R.T. McMullin; occurrenceID: A1C96C40-E1D9-56FC-A24E-C65BBFA62A4E; **Location:** locationID: XIX; decimalLatitude: 51.65065; decimalLongitude: -128.14241; **Identification:** identificationRemarks: TLC: atranorin and planaic acid; **Event:** habitat: Lignicolous on a stump; **Record Level:** institutionID: CANL; collectionID: McMullin 19725
- **Type status:** Other material. **Occurrence:** recordedBy: R.T. McMullin; occurrenceID: F1B58D9D-BF3D-5F1E-9853-230D5F8A9EC9; **Location:** locationID: XXII; decimalLatitude: 51.64502; decimalLongitude: -128.15099; **Identification:** identificationRemarks: TLC: atranorin and planaic acid; **Event:** habitat: Corticolous on *Piceasitchensis*; **Record Level:** institutionID: CANL; collectionID: McMullin 19729
- **Type status:** Other material. **Occurrence:** catalogNumber: BOLD PHAK348-20; recordedBy: A. Simon; otherCatalogNumbers: GenBank OQ922957; occurrenceID: EA6F3125-FFE7-50FB-8728-C8CADC4B915D; **Location:** locationID: XII; decimalLatitude: 51.66040; decimalLongitude: -128.11688; **Record Level:** institutionID: UBC; collectionID: Simon 792

#### Mycoblastus caesius

(Coppins & P. James) Tønsberg

E05DBC85-AB29-53A0-A49F-07C462E82F70

##### Materials

- **Type status:** Other material. **Occurrence:** recordedBy: R.T. McMullin; occurrenceID: 3325F05E-6A78-5054-9D8E-758231D4A758; **Location:** locationID: XX; decimalLatitude: 51.64809; decimalLongitude: -128.14378; **Event:** habitat: Corticolous on Pinuscontortassp.contorta; **Record Level:** institutionID: CANL; collectionID: McMullin 19516
- **Type status:** Other material. **Occurrence:** catalogNumber: BOLD CALV053-20; recordedBy: R.T. McMullin; otherCatalogNumbers: GenBank OQ843308; occurrenceID: AA7F8D42-A170-5353-8DEC-CA67EDB0F13C; **Location:** locationID: V; decimalLatitude: 51.62022; decimalLongitude: -127.93070; **Event:** habitat: Corticolous on *Alnusrubra*; **Record Level:** institutionID: CANL; collectionID: McMullin 19585
- **Type status:** Other material. **Occurrence:** recordedBy: R.T. McMullin; occurrenceID: 79B4303F-3FA2-5E6B-A865-EBAF633B9940; **Location:** locationID: V; decimalLatitude: 51.62022; decimalLongitude: -127.93070; **Event:** habitat: Lignicolous; **Record Level:** institutionID: CANL; collectionID: McMullin 19673
- **Type status:** Other material. **Occurrence:** recordedBy: R.T. McMullin; occurrenceID: 66F9F58E-F6EE-5622-B21C-535C5CBD99DD; **Location:** locationID: XI; decimalLatitude: 51.61622; decimalLongitude: -127.94227; **Event:** habitat: Corticolous on *Alnusrubra*; **Record Level:** institutionID: CANL; collectionID: McMullin 19701
- **Type status:** Other material. **Occurrence:** recordedBy: R.T. McMullin; occurrenceID: 4BC2E017-7546-533D-9681-E561ABA1B319; **Location:** locationID: IV; decimalLatitude: 51.65514; decimalLongitude: -128.13243; **Event:** habitat: Corticolous on *Alnusrubra*; **Record Level:** institutionID: CANL; collectionID: McMullin 19884

#### Mycoblastus sanguinarius

(L.) Norman

638E6EEB-7555-5300-815E-D682B1F618BA

##### Materials

- **Type status:** Other material. **Occurrence:** catalogNumber: BOLD CALV057-20; recordedBy: R.T. McMullin; otherCatalogNumbers: GenBank OQ843261; occurrenceID: 04C917ED-AAC4-5A45-A1BB-A9727E5CC066; **Location:** locationID: XX; decimalLatitude: 51.64809; decimalLongitude: -128.14378; **Event:** habitat: Corticolous on Pinuscontortassp.contorta; **Record Level:** institutionID: CANL; collectionID: McMullin 19510
- **Type status:** Other material. **Occurrence:** catalogNumber: BOLD CALV171-20; recordedBy: R.T. McMullin; otherCatalogNumbers: GenBank OQ843219; occurrenceID: E6113E1A-F0DE-5879-A8DF-41A87FD38005; **Location:** locationID: XII; decimalLatitude: 51.66040; decimalLongitude: -128.11688; **Identification:** identificationRemarks: TLC: atranorin; **Event:** habitat: Corticolous and lignicolous on a conifer snag; **Record Level:** institutionID: CANL; collectionID: McMullin 19675
- **Type status:** Other material. **Occurrence:** catalogNumber: BOLD CALV055-20; recordedBy: R.T. McMullin; otherCatalogNumbers: GenBank OQ843223; occurrenceID: 1BD37A4A-9CA3-51E2-A250-CE81D255B409; **Location:** locationID: XXIII; decimalLatitude: 51.64058; decimalLongitude: -128.14882; **Event:** habitat: Lignicolous on a conifer snag; **Record Level:** institutionID: CANL; collectionID: McMullin 19691
- **Type status:** Other material. **Occurrence:** catalogNumber: BOLD CALV056-20; recordedBy: R.T. McMullin; otherCatalogNumbers: GenBank OQ843263; occurrenceID: A1EA8120-A5F1-5E12-B1F9-F752C7A1B627; **Location:** locationID: XIX; decimalLatitude: 51.65065; decimalLongitude: -128.14241; **Event:** habitat: Lignicolous on a snag; **Record Level:** institutionID: CANL; collectionID: McMullin 19741

#### Nephroma helveticum sipeanum

(Gyeln.) Goward & Ahti

A49178BA-49E7-5409-B57A-056DC9BDE6A1

##### Materials

- **Type status:** Other material. **Occurrence:** catalogNumber: BOLD CALV185-20; recordedBy: R.T. McMullin; otherCatalogNumbers: GenBank OQ843288; occurrenceID: B6BAEB56-AC14-593D-B7C3-27E6C7A3E567; **Location:** locationID: XIII; decimalLatitude: 51.66854; decimalLongitude: -128.11832; **Event:** habitat: Corticolous on *Alnusrubra*; **Record Level:** institutionID: CANL; collectionID: McMullin 19601
- **Type status:** Other material. **Occurrence:** catalogNumber: BOLD CALV058-20; recordedBy: R.T. McMullin; otherCatalogNumbers: GenBank OQ843211; occurrenceID: 16CC286A-CEA0-54B8-841B-B7963220E815; **Location:** locationID: XIII; decimalLatitude: 51.66854; decimalLongitude: -128.11832; **Event:** habitat: Corticolous on *Alnusrubra*; **Record Level:** institutionID: CANL; collectionID: McMullin 19696
- **Type status:** Other material. **Occurrence:** catalogNumber: BOLD CALV061-20; recordedBy: R.T. McMullin; otherCatalogNumbers: GenBank OQ843281; occurrenceID: 8D9B0705-0619-5140-8859-85D1D5C4F5C0; **Location:** locationID: XVII; decimalLatitude: 51.66213; decimalLongitude: -128.14492; **Event:** habitat: Corticolous on *Alnusrubra*; **Record Level:** institutionID: CANL; collectionID: McMullin 19727
- **Type status:** Other material. **Occurrence:** recordedBy: R.T. McMullin; occurrenceID: 1D547A22-A48B-5B9C-A683-37AE51969D14; **Location:** locationID: XIII; decimalLatitude: 51.66854; decimalLongitude: -128.11832; **Event:** habitat: Corticolous on *Alnusrubra*; **Record Level:** institutionID: CANL; collectionID: McMullin 19850

##### Notes

Our specimens were somewhat intermediate between subsp. helveticum and subsp. sipeanum. We have assigned subsp. sipeanum since it appears to be the dominant taxon in the region.

#### Nephroma laevigatum

Ach.

552EDBAD-22CE-500B-A28A-D559B571DAE2

##### Materials

- **Type status:** Other material. **Occurrence:** catalogNumber: BOLD CALV062-20; recordedBy: R.T. McMullin; otherCatalogNumbers: GenBank OQ843328; occurrenceID: DFD83A1A-FAAE-5274-961D-0F9C38DA1080; **Location:** locationID: XIII; decimalLatitude: 51.66854; decimalLongitude: -128.11832; **Event:** habitat: Corticolous on *Alnusrubra*; **Record Level:** institutionID: CANL; collectionID: McMullin 19594
- **Type status:** Other material. **Occurrence:** catalogNumber: BOLD CALV063-20; recordedBy: R.T. McMullin; otherCatalogNumbers: GenBank OQ843209; occurrenceID: 95F69E9A-4307-5EB0-9A2D-AC5D59BB1BDB; **Location:** locationID: III; decimalLatitude: 51.65486; decimalLongitude: -128.13907; **Event:** habitat: Corticolous on *Piceasitchensis*; **Record Level:** institutionID: CANL; collectionID: McMullin 19732
- **Type status:** Other material. **Occurrence:** catalogNumber: BOLD PHAK380-20; recordedBy: A. Simon; otherCatalogNumbers: GenBank OQ922946; occurrenceID: 7419E7BD-BDE7-5F18-A716-9DAD030D860E; **Location:** locationID: XXI; decimalLatitude: 51.64221; decimalLongitude: -128.15085; **Record Level:** institutionID: UBC; collectionID: Simon 810

#### Nephroma parile

(Ach.) Ach.

87AAF73C-C24D-54A0-B832-CF2CF0F13C6E

##### Materials

- **Type status:** Other material. **Occurrence:** catalogNumber: BOLD CALV064-20; recordedBy: R.T. McMullin; otherCatalogNumbers: GenBank OQ843231; occurrenceID: F176FFFD-560E-5C9E-ADF9-8B01DDB18995; **Location:** locationID: XVI; decimalLatitude: 51.66051; decimalLongitude: -128.14587; **Event:** habitat: Corticolous on *Alnusrubra*; **Record Level:** institutionID: CANL; collectionID: McMullin 19502
- **Type status:** Other material. **Occurrence:** catalogNumber: BOLD CALV065-20; recordedBy: R.T. McMullin; otherCatalogNumbers: GenBank OQ843274; occurrenceID: 4200F7D9-F1C5-54AB-9AAF-E1C40CE76371; **Location:** locationID: XIII; decimalLatitude: 51.66854; decimalLongitude: -128.11832; **Event:** habitat: Bryicolous on *Alnusrubra*; **Record Level:** institutionID: CANL; collectionID: McMullin 19575

#### Nephroma resupinatum

(L.) Ach.

54C4B0C8-0208-51CA-B8D4-9D8772426397

##### Materials

- **Type status:** Other material. **Occurrence:** catalogNumber: BOLD CALV066-20; recordedBy: R.T. McMullin; otherCatalogNumbers: GenBank OQ843287; occurrenceID: 1EF8F8FD-3054-533E-8C9F-54C9FAE3CB07; **Location:** locationID: III; decimalLatitude: 51.65486; decimalLongitude: -128.13907; **Event:** habitat: Corticolous on *Alnusrubra*; **Record Level:** institutionID: CANL; collectionID: McMullin 19553
- **Type status:** Other material. **Occurrence:** catalogNumber: BOLD PHAK354-20; recordedBy: A. Simon; otherCatalogNumbers: GenBank OQ922983; occurrenceID: 9245EBED-CD61-57FB-A3B0-FBC8F624ADCC; **Location:** locationID: XXI; decimalLatitude: 51.64221; decimalLongitude: -128.15085; **Record Level:** institutionID: UBC; collectionID: Simon 848

#### Normandina pulchella

(Borrer) Nyl.

5E6C1297-C3FA-5728-8D27-4074574E8C3D

##### Materials

- **Type status:** Other material. **Occurrence:** recordedBy: R.T. McMullin; occurrenceID: A26DEFEF-CB59-5452-964B-F46013027051; **Location:** locationID: III; decimalLatitude: 51.65486; decimalLongitude: -128.13907; **Event:** habitat: Lichenicolous on *Fuscopannaria* on *Alnusrubra*; **Record Level:** institutionID: CANL; collectionID: McMullin 19554
- **Type status:** Other material. **Occurrence:** recordedBy: R.T. McMullin; occurrenceID: 485F0FCF-7624-5309-B73B-A3EEFE0E60B8; **Location:** locationID: XVI; decimalLatitude: 51.66051; decimalLongitude: -128.14587; **Identification:** identificationRemarks: Figure 3F; **Event:** habitat: Bryicolous on *Alnusrubra*; **Record Level:** institutionID: CANL; collectionID: McMullin 19718
- **Type status:** Other material. **Occurrence:** recordedBy: R.T. McMullin; occurrenceID: FE243E91-AD43-5400-923F-288347272897; **Location:** locationID: II; decimalLatitude: 51.65285; decimalLongitude: -128.13873; **Event:** habitat: Lichenicolous; **Record Level:** institutionID: CANL; collectionID: McMullin 19761

#### Ochrolechia frigida

(Sw.) Lynge

E180AAE0-F3CD-54BF-895D-F2BBE1A70E93

##### Materials

- **Type status:** Other material. **Occurrence:** recordedBy: R.T. McMullin; occurrenceID: D6A80105-C731-575B-B175-F0114F3A613A; **Location:** locationID: XIV; decimalLatitude: 51.66797; decimalLongitude: -128.12128; **Identification:** identificationRemarks: TLC: gyrophoric acid; **Event:** habitat: Terricolous; **Record Level:** institutionID: CANL; collectionID: McMullin 19806
- **Type status:** Other material. **Occurrence:** recordedBy: A. Simon; occurrenceID: 6F4248DD-2322-5075-B767-1B5C049B15CF; **Location:** locationID: XI; decimalLatitude: 51.61622; decimalLongitude: -127.94227; **Record Level:** institutionID: UBC; collectionID: Simon 829

#### Ochrolechia juvenalis

Brodo

87A9FAC2-A2F9-5483-BD46-F425BE65B23D

##### Materials

- **Type status:** Other material. **Occurrence:** recordedBy: R.T. McMullin; occurrenceID: CDDC4CAE-58EC-58F2-A5F0-FA0ADE4423BB; **Location:** locationID: XX; decimalLatitude: 51.64809; decimalLongitude: -128.14378; **Event:** habitat: Lignicolous on Pinuscontortassp.contorta; **Record Level:** institutionID: CANL; collectionID: McMullin 19828

#### Ochrolechia mahluensis

Räsänen

5444D8D6-78F0-5655-B61F-05E1439C5CF4

##### Materials

- **Type status:** Other material. **Occurrence:** recordedBy: R.T. McMullin; occurrenceID: F399720E-E542-51D0-BBBB-504F92E8FACF; **Location:** locationID: XX; decimalLatitude: 51.64809; decimalLongitude: -128.14378; **Identification:** identificationRemarks: TLC: gyrophoric acid, no fatty acids; **Event:** habitat: Bryicolous; **Record Level:** institutionID: CANL; collectionID: McMullin 19807

#### Ochrolechia oregonensis

H. Magn.

F811438B-8430-5575-B2A4-D3BE8E570437

##### Materials

- **Type status:** Other material. **Occurrence:** catalogNumber: BOLD CALV072-20; recordedBy: R.T. McMullin; otherCatalogNumbers: GenBank OQ843356; occurrenceID: 4E246810-2D9A-5C5C-B667-E89AFB700A4D; **Location:** locationID: XXVI; decimalLatitude: 51.65466; decimalLongitude: -128.13051; **Event:** habitat: Corticolous on *Piceasitchensis*; **Record Level:** institutionID: CANL; collectionID: McMullin 19766
- **Type status:** Other material. **Occurrence:** catalogNumber: BOLD CALV073-20; recordedBy: R.T. McMullin; otherCatalogNumbers: GenBank OQ843355; occurrenceID: 9EEB44B5-1DB9-5A8C-B5CC-CE8A4C7935B5; **Location:** locationID: XVI; decimalLatitude: 51.66051; decimalLongitude: -128.14587; **Event:** habitat: Corticolous on a conifer; **Record Level:** institutionID: CANL; collectionID: McMullin 19829
- **Type status:** Other material. **Occurrence:** recordedBy: R.T. McMullin; occurrenceID: 981A54DC-23FF-54E2-977F-0CA4D383A530; **Location:** locationID: VII; decimalLatitude: 51.61615; decimalLongitude: -127.93851; **Event:** habitat: Corticolous on a conifer snag; **Record Level:** institutionID: CANL; collectionID: McMullin 19830
- **Type status:** Other material. **Occurrence:** catalogNumber: BOLD CALV074-20; recordedBy: R.T. McMullin; otherCatalogNumbers: GenBank OQ843243; occurrenceID: CE1C4380-EAAE-5B54-A0AD-CFE6B0F7428A; **Location:** locationID: XII; decimalLatitude: 51.66040; decimalLongitude: -128.11688; **Event:** habitat: Lignicolous on a snag; **Record Level:** institutionID: CANL; collectionID: McMullin 19831
- **Type status:** Other material. **Occurrence:** catalogNumber: BOLD PHAK301-20; recordedBy: A. Simon; otherCatalogNumbers: GenBank OQ922942; occurrenceID: 5DD9E8B9-F3F3-5BB6-B855-7D79404A7761; **Location:** locationID: XII; decimalLatitude: 51.66040; decimalLongitude: -128.11688; **Record Level:** institutionID: UBC; collectionID: Simon 793

#### Ochrolechia subathallina

H. Magn.

A84E2E4B-53E0-5BD8-841A-1562DD7B09C3

##### Materials

- **Type status:** Other material. **Occurrence:** catalogNumber: BOLD PHAK367-20; recordedBy: A. Simon; otherCatalogNumbers: GenBank OQ922944; occurrenceID: B685BBFE-92F3-5110-9732-0B25F97C8B05; **Location:** locationID: V; decimalLatitude: 51.62022; decimalLongitude: -127.93070; **Record Level:** institutionID: UBC; collectionID: Simon 828

#### Opegrapha sphaerophoricola

Isbrand & Alstrup

B39F314D-6628-5109-959B-74997D2FD89A

##### Materials

**Figure 4.**
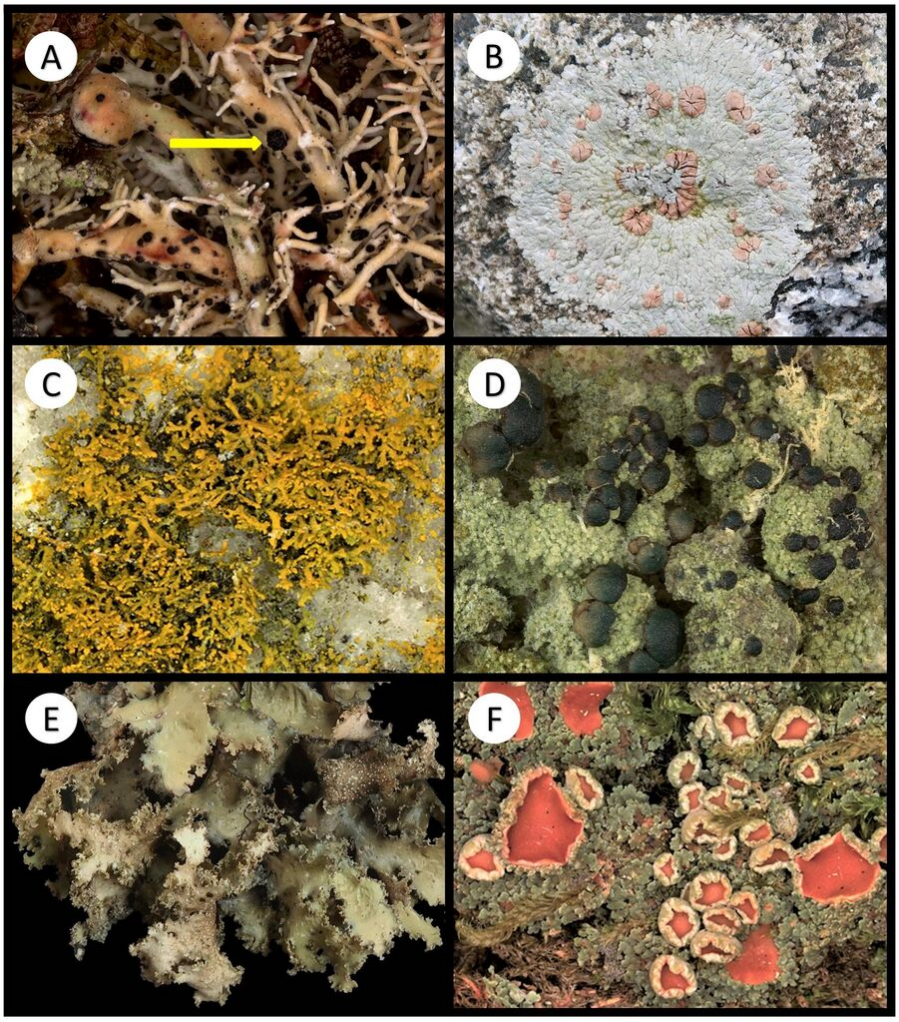
Lichens of Calvert Island. **A.** *Opegraphasphaerophoricola* on *Sphaerophorustuckermanii*, *McMullin 19508* (CANL). **B.** *Placopsislambii*, *McMullin 19667* (CANL). **C.** *Polycauliona* sp., McMullin *19716* (CANL). **D.** *Protomicarealimosa*, *McMullin 19698* (CANL). **E.** *Pseudocyphellariarainierensis*, *McMullin 19606* (CANL). **F.** *Psoromahypnorum*, *McMullin 19719* (CANL).

- **Type status:** Other material. **Occurrence:** recordedBy: R.T. McMullin; occurrenceID: EDD494BE-5A0B-59BD-B2CB-F4D7F11F1EBA; **Location:** locationID: III; decimalLatitude: 51.65486; decimalLongitude: -128.13907; **Identification:** identificationRemarks: Figure 4A; **Event:** habitat: Lichenicolous on *Sphaerophorustuckermanii*; **Record Level:** institutionID: CANL; collectionID: McMullin 19508

##### Parasite of

*Sphaerophorustuckermanii*

##### Notes

Non-lichenised fungus.

#### Parmelia saxatilis

(L.) Ach.

1E170C52-8D23-57A2-B0ED-27AEEFD532C6

##### Materials

- **Type status:** Other material. **Occurrence:** catalogNumber: BOLD CALV076-20; recordedBy: R.T. McMullin; otherCatalogNumbers: GenBank OQ843242; occurrenceID: B796A5F3-AB4D-54E8-AE40-B0BAC6076839; **Location:** locationID: XII; decimalLatitude: 51.66040; decimalLongitude: -128.11688; **Event:** habitat: Terricolous; **Record Level:** institutionID: CANL; collectionID: McMullin 19706
- **Type status:** Other material. **Occurrence:** catalogNumber: BOLD CALV077-20; recordedBy: R.T. McMullin; otherCatalogNumbers: GenBank OQ843212; occurrenceID: 9797F779-3B4D-51EF-9091-C6E8D7E7ACBE; **Location:** locationID: II; decimalLatitude: 51.65285; decimalLongitude: -128.13873; **Event:** habitat: Saxicolous; **Record Level:** institutionID: CANL; collectionID: McMullin 19764
- **Type status:** Other material. **Occurrence:** catalogNumber: BOLD PHAK330-20; recordedBy: A. Simon; otherCatalogNumbers: GenBank OQ922981; occurrenceID: 5557354F-8B48-58BE-A8C7-D15203EF7CF8; **Location:** locationID: XXX; decimalLatitude: 51.63722; decimalLongitude: -128.09525; **Record Level:** institutionID: UBC; collectionID: Simon 770
- **Type status:** Other material. **Occurrence:** recordedBy: A. Simon; occurrenceID: BCA65FB9-CCF3-5F77-B4E1-1C46E94E7E88; **Location:** locationID: XII; decimalLatitude: 51.66040; decimalLongitude: -128.11688; **Record Level:** institutionID: UBC; collectionID: Simon 794
- **Type status:** Other material. **Occurrence:** catalogNumber: BOLD PHAK296-20; recordedBy: A. Simon; otherCatalogNumbers: GenBank OQ922969; occurrenceID: 5C0DCB6D-3F5D-55AD-AFF4-BAC97C44422F; **Location:** locationID: XII; decimalLatitude: 51.66040; decimalLongitude: -128.11688; **Record Level:** institutionID: UBC; collectionID: Simon 795
- **Type status:** Other material. **Occurrence:** recordedBy: A. Simon; occurrenceID: F08CAB54-A96F-58AA-B129-E271DA7D08A6; **Location:** locationID: XXI; decimalLatitude: 51.64221; decimalLongitude: -128.15085; **Record Level:** institutionID: UBC; collectionID: Simon 811
- **Type status:** Other material. **Occurrence:** recordedBy: A. Simon; occurrenceID: 8A76EE48-EF84-5AB9-92C0-677922F61BB9; **Location:** locationID: XIV; decimalLatitude: 51.66797; decimalLongitude: -128.12128; **Record Level:** institutionID: UBC; collectionID: Simon 812

#### Parmelia squarrosa

Hale

F9CADCE8-E976-5407-98E1-E979D6B7C9E8

##### Materials

- **Type status:** Other material. **Occurrence:** recordedBy: A. Simon; occurrenceID: B448DB8F-55D9-5742-BAEC-1B8DC5AA8012; **Location:** locationID: XVII; decimalLatitude: 51.66213; decimalLongitude: -128.14492; **Record Level:** institutionID: UBC; collectionID: Simon 755
- **Type status:** Other material. **Occurrence:** recordedBy: A. Simon; occurrenceID: E225A68A-5536-5700-9239-2341E2AA7B0A; **Location:** locationID: XXI; decimalLatitude: 51.64221; decimalLongitude: -128.15085; **Record Level:** institutionID: UBC; collectionID: Simon 854

#### Parmelia sulcata

Taylor

AF881259-CF6B-5A90-922C-DB3262F8B9A0

##### Materials

- **Type status:** Other material. **Occurrence:** catalogNumber: BOLD CALV080-20; recordedBy: R.T. McMullin; otherCatalogNumbers: GenBank OQ843234; occurrenceID: AFE29DEB-9F32-5F72-BCF8-9DEFF034DB01; **Location:** locationID: XI; decimalLatitude: 51.61622; decimalLongitude: -127.94227; **Event:** habitat: Corticolous on *Alnusrubra*; **Record Level:** institutionID: CANL; collectionID: McMullin 19654
- **Type status:** Other material. **Occurrence:** catalogNumber: BOLD CALV081-20; recordedBy: R.T. McMullin; otherCatalogNumbers: GenBank OQ843198; occurrenceID: E929096D-407E-50E7-8AE1-C457FDD9177D; **Location:** locationID: XIII; decimalLatitude: 51.66854; decimalLongitude: -128.11832; **Event:** habitat: Corticolous on *Alnusrubra*; **Record Level:** institutionID: CANL; collectionID: McMullin 19702
- **Type status:** Other material. **Occurrence:** catalogNumber: BOLD PHAK369-20; recordedBy: A. Simon; otherCatalogNumbers: GenBank OQ922952; occurrenceID: BB9E80B1-6576-52B8-BB5C-D81A6472BA0E; **Location:** locationID: XI; decimalLatitude: 51.61622; decimalLongitude: -127.94227; **Record Level:** institutionID: UBC; collectionID: Simon 830

#### Parmeliopsis hyperopta

(Ach.) Arnold

C6427F61-3F22-566F-9247-1F0335CFF5DA

##### Materials

- **Type status:** Other material. **Occurrence:** catalogNumber: BOLD CALV082-20; recordedBy: R.T. McMullin; otherCatalogNumbers: GenBank OQ843299; occurrenceID: B8851B01-5653-5D72-A779-A9E15116072D; **Location:** locationID: IX; decimalLatitude: 51.61638; decimalLongitude: -127.94035; **Event:** habitat: Lignicolous on a snag; **Record Level:** institutionID: CANL; collectionID: McMullin 19678
- **Type status:** Other material. **Occurrence:** catalogNumber: BOLD PHAK366-20; recordedBy: A. Simon; otherCatalogNumbers: GenBank OQ922934; occurrenceID: 3C4FF613-A98D-5F8C-90B8-3D9FCF8BF43B; **Location:** locationID: IX; decimalLatitude: 51.61638; decimalLongitude: -127.94035; **Record Level:** institutionID: UBC; collectionID: Simon 831

#### Peltigera britannica

(Gyeln.) Holt.-Hartw. & Tønsberg

B57F8B38-9BF6-5DEB-8D22-0D845328C5BB

##### Materials

- **Type status:** Other material. **Occurrence:** recordedBy: R.T. McMullin; occurrenceID: 4ABC469B-7428-52D0-B5B5-8F7D4F6EE1F3; **Location:** locationID: V; decimalLatitude: 51.62022; decimalLongitude: -127.93070; **Event:** habitat: Terricolous; **Record Level:** institutionID: CANL; collectionID: McMullin 19648
- **Type status:** Other material. **Occurrence:** catalogNumber: BOLD CALV084-20; recordedBy: R.T. McMullin; otherCatalogNumbers: GenBank OQ843353; occurrenceID: 00FAC3C1-21CD-59E3-862B-29458BDCA5C2; **Location:** locationID: XIII; decimalLatitude: 51.66854; decimalLongitude: -128.11832; **Event:** habitat: Bryicolous; **Record Level:** institutionID: CANL; collectionID: McMullin 19657
- **Type status:** Other material. **Occurrence:** catalogNumber: BOLD CALV085-20; recordedBy: R.T. McMullin; otherCatalogNumbers: GenBank OQ843284; occurrenceID: 39C0F557-AADB-52BA-84F5-496A76544A35; **Location:** locationID: XIX; decimalLatitude: 51.65065; decimalLongitude: -128.14241; **Event:** habitat: Terricolous; **Record Level:** institutionID: CANL; collectionID: McMullin 19742
- **Type status:** Other material. **Occurrence:** catalogNumber: BOLD PHAK340-20; recordedBy: A. Simon; otherCatalogNumbers: GenBank OQ922945; occurrenceID: F27B63DC-2BC5-5E13-8848-25FA9BC85CF0; **Location:** locationID: XVI; decimalLatitude: 51.66051; decimalLongitude: -128.14587; **Record Level:** institutionID: UBC; collectionID: Simon 756

#### Peltigera collina

(Ach.) Schrad.

EC983999-A9D5-50F5-BC9A-E8DA7B2AA49F

##### Materials

- **Type status:** Other material. **Occurrence:** catalogNumber: BOLD CALV086-20; recordedBy: R.T. McMullin; otherCatalogNumbers: GenBank OQ843282; occurrenceID: E8C2B173-84CB-5BED-95FC-B76039FB459F; **Location:** locationID: XIII; decimalLatitude: 51.66854; decimalLongitude: -128.11832; **Event:** habitat: Corticolous on *Alnusrubra*; **Record Level:** institutionID: CANL; collectionID: McMullin 19605
- **Type status:** Other material. **Occurrence:** recordedBy: A. Simon; occurrenceID: 23415AFE-4B14-55EB-9118-C1D32B09C227; **Location:** locationID: XIII; decimalLatitude: 51.66854; decimalLongitude: -128.11832; **Record Level:** institutionID: UBC; collectionID: Simon 796

#### Peltigera membranacea

(Ach.) Nyl.

C02A907C-6021-519E-9F50-D51D4736028F

##### Materials

- **Type status:** Other material. **Occurrence:** catalogNumber: BOLD CALV087-20; recordedBy: R.T. McMullin; otherCatalogNumbers: GenBank OQ843260; occurrenceID: 63853F3D-99C5-57EF-A34B-AA740F16E83C; **Location:** locationID: III; decimalLatitude: 51.65486; decimalLongitude: -128.13907; **Event:** habitat: Terricolous; **Record Level:** institutionID: CANL; collectionID: McMullin 19536
- **Type status:** Other material. **Occurrence:** catalogNumber: BOLD CALV088-20; recordedBy: R.T. McMullin; otherCatalogNumbers: GenBank OQ843214; occurrenceID: 24A79817-44C1-58AC-898B-05E409323F1A; **Location:** locationID: I; decimalLatitude: 51.65501; decimalLongitude: -128.13799; **Event:** habitat: Lignicolous on a stump; **Record Level:** institutionID: CANL; collectionID: McMullin 19740
- **Type status:** Other material. **Occurrence:** catalogNumber: BOLD PHAK347-20; recordedBy: A. Simon; otherCatalogNumbers: GenBank OQ922940; occurrenceID: 60A68791-167E-5B8F-AF52-FB62AC2443FE; **Location:** locationID: XVI; decimalLatitude: 51.66051; decimalLongitude: -128.14587; **Record Level:** institutionID: UBC; collectionID: Simon 798

#### Peltigera nigriventris

Magain, Goward, Miadl. & Sérus.

E939AC2C-3871-5FCA-B4D1-6746A1CAD281

##### Materials

- **Type status:** Other material. **Occurrence:** catalogNumber: BOLD CALV089-20; recordedBy: R.T. McMullin; otherCatalogNumbers: GenBank OQ843238; occurrenceID: FD857C76-D260-5549-8450-0AB2DF0818D9; **Location:** locationID: III; decimalLatitude: 51.65486; decimalLongitude: -128.13907; **Event:** habitat: Terricolous; **Record Level:** institutionID: CANL; collectionID: McMullin 19505
- **Type status:** Other material. **Occurrence:** catalogNumber: BOLD CALV090-20; recordedBy: R.T. McMullin; otherCatalogNumbers: GenBank OQ843249; occurrenceID: 4C0AFA40-3247-5D66-B9F2-2AE51FEA90D3; **Location:** locationID: III; decimalLatitude: 51.65486; decimalLongitude: -128.13907; **Event:** habitat: Terricolous; **Record Level:** institutionID: CANL; collectionID: McMullin 19527
- **Type status:** Other material. **Occurrence:** catalogNumber: BOLD CALV091-20; recordedBy: R.T. McMullin; otherCatalogNumbers: GenBank OQ843324; occurrenceID: 4B192EAF-E585-5917-959E-157C901063CD; **Location:** locationID: XVI; decimalLatitude: 51.66051; decimalLongitude: -128.14587; **Event:** habitat: Terricolous; **Record Level:** institutionID: CANL; collectionID: McMullin 19662

#### Peltigera pacifica

Vitik.

9B1741F6-D597-5DE2-B597-FEC596FBE17C

##### Materials

- **Type status:** Other material. **Occurrence:** catalogNumber: BOLD CALV092-20; recordedBy: R.T. McMullin; otherCatalogNumbers: GenBank OQ843319; occurrenceID: 69C1681E-7DAF-5710-8BDE-1DCED36B0558; **Location:** locationID: X; decimalLatitude: 51.61977; decimalLongitude: -127.93245; **Event:** habitat: Terricolous; **Record Level:** institutionID: CANL; collectionID: McMullin 19626

#### Peltigera scabrosa

Th. Fr.

504F66F4-2E40-5566-BC4E-1986B0380EE2

##### Materials

- **Type status:** Other material. **Occurrence:** recordedBy: R.T. McMullin; occurrenceID: CD9878C8-F77F-5E89-A0D6-F26E84EE175C; **Location:** locationID: III; decimalLatitude: 51.65486; decimalLongitude: -128.13907; **Event:** habitat: Terricolous; **Record Level:** institutionID: CANL; collectionID: McMullin 19552
- **Type status:** Other material. **Occurrence:** catalogNumber: BOLD CALV161-20; recordedBy: R.T. McMullin; otherCatalogNumbers: GenBank OQ843233; occurrenceID: A38ED9FF-52E8-5999-B570-BB7A3CD50474; **Location:** locationID: XVII; decimalLatitude: 51.66213; decimalLongitude: -128.14492; **Event:** habitat: Terricolous; **Record Level:** institutionID: CANL; collectionID: McMullin 19730
- **Type status:** Other material. **Occurrence:** catalogNumber: BOLD PHAK325-20; recordedBy: A. Simon; otherCatalogNumbers: GenBank OQ922988; occurrenceID: C3EE719D-CF72-57C3-BC24-93AC06228E88; **Location:** locationID: XIII; decimalLatitude: 51.66854; decimalLongitude: -128.11832; **Record Level:** institutionID: UBC; collectionID: Simon 797
- **Type status:** Other material. **Occurrence:** catalogNumber: BOLD PHAK378-20; recordedBy: A. Simon; otherCatalogNumbers: GenBank OQ922938; occurrenceID: F1A959BB-63D7-5C18-9B5B-0B8403CA1554; **Location:** locationID: X; decimalLatitude: 51.61977; decimalLongitude: -127.93245; **Record Level:** institutionID: UBC; collectionID: Simon 833

#### Peltigera vitikainenii

Magain, Miadl., Goward & Sérus.

C46B5F79-ECC5-52E4-B44F-C5AD2FEE87BA

##### Materials

- **Type status:** Other material. **Occurrence:** catalogNumber: BOLD CALV167-20; recordedBy: R.T. McMullin; otherCatalogNumbers: GenBank OQ843304; occurrenceID: 23CD97F0-9130-5067-A5E5-BB697E4945B4; **Location:** locationID: II; decimalLatitude: 51.65285; decimalLongitude: -128.13873; **Event:** habitat: Terricolous on a rock; **Record Level:** institutionID: CANL; collectionID: McMullin 19753

#### Pertusaria sp.

284CA9B8-4EB8-52EF-B64F-4FE76CB2869A

##### Materials

- **Type status:** Other material. **Occurrence:** recordedBy: R.T. McMullin; occurrenceID: 18130673-DF4D-5253-BC72-7B77193CB15A; **Location:** locationID: V; decimalLatitude: 51.62022; decimalLongitude: -127.93070; **Event:** habitat: Corticolous on *Alnusrubra*; **Record Level:** institutionID: CANL; collectionID: McMullin 19584
- **Type status:** Other material. **Occurrence:** recordedBy: R.T. McMullin; occurrenceID: AA778450-CBE7-5300-A7C9-AD1509AB932A; **Location:** locationID: XIII; decimalLatitude: 51.66854; decimalLongitude: -128.11832; **Event:** habitat: Corticolous on *Alnusrubra*; **Record Level:** institutionID: CANL; collectionID: McMullin 19876

##### Notes

These specimens were not developed enough for species level identification.

#### Phaeocalicium compressulum

(Nyl. ex Vain.) A.F.W. Schmidt

8ECE80C6-F7FB-5261-95C2-B6EAE42E04A5

##### Materials

- **Type status:** Other material. **Occurrence:** recordedBy: R.T. McMullin; occurrenceID: 3BFE38ED-4E41-538D-8D0A-115305ABBEAA; **Location:** locationID: V; decimalLatitude: 51.62022; decimalLongitude: -127.93070; **Event:** habitat: Corticolous on *Alnusrubra*; **Record Level:** institutionID: CANL; collectionID: McMullin 19642
- **Type status:** Other material. **Occurrence:** catalogNumber: BOLD CALV095-20; recordedBy: R.T. McMullin; otherCatalogNumbers: GenBank OQ843226; occurrenceID: 551AAE36-6666-55A8-919D-411B67EC853B; **Location:** locationID: IX; decimalLatitude: 51.61638; decimalLongitude: -127.94035; **Event:** habitat: Corticolous on *Alnusrubra*; **Record Level:** institutionID: CANL; collectionID: McMullin 19659
- **Type status:** Other material. **Occurrence:** catalogNumber: BOLD CALV104-20; recordedBy: R.T. McMullin; otherCatalogNumbers: GenBank OQ843295; occurrenceID: D6616230-5D06-5568-B278-639C8212F60D; **Location:** locationID: XII; decimalLatitude: 51.66040; decimalLongitude: -128.11688; **Event:** habitat: Corticolous on *Alnusrubra*; **Record Level:** institutionID: CANL; collectionID: McMullin 19708
- **Type status:** Other material. **Occurrence:** recordedBy: R.T. McMullin; occurrenceID: 3CB27AFD-BA62-53D1-A804-746A96277B3F; **Location:** locationID: III; decimalLatitude: 51.65486; decimalLongitude: -128.13907; **Event:** habitat: Corticolous on *Alnusrubra*; **Record Level:** institutionID: CANL; collectionID: McMullin 19871

##### Notes

Non-lichenised fungus.

#### Physcia adscendens

(Fr.) H. Olivier

E0D96AC6-DE04-56B5-AD87-0F347765C4D2

##### Materials

- **Type status:** Other material. **Occurrence:** catalogNumber: BOLD CALV106-20; recordedBy: R.T. McMullin; otherCatalogNumbers: GenBank OQ843363; occurrenceID: 6532E83F-3EF1-5EC4-BB47-229F35426D60; **Location:** locationID: XII; decimalLatitude: 51.66040; decimalLongitude: -128.11688; **Event:** habitat: Saxicolous; **Record Level:** institutionID: CANL; collectionID: McMullin 19521

#### Physcia caesia

(Hoffm.) Hampe ex Fürnr.

EA2CEB21-315D-567E-8415-AC58CD726899

##### Materials

- **Type status:** Other material. **Occurrence:** catalogNumber: BOLD CALV105-20; recordedBy: R.T. McMullin; otherCatalogNumbers: GenBank OQ843203; occurrenceID: 25DA9697-D967-5399-9395-A1442C736C62; **Location:** locationID: V; decimalLatitude: 51.62022; decimalLongitude: -127.93070; **Event:** habitat: Saxicolous; **Record Level:** institutionID: CANL; collectionID: McMullin 19683

#### Pilophorus acicularis

(Ach.) Th. Fr.

65D0A7AD-23F5-5299-91F7-57D1A52E57BB

##### Materials

- **Type status:** Other material. **Occurrence:** catalogNumber: BOLD CALV107-20; recordedBy: R.T. McMullin; otherCatalogNumbers: GenBank OQ843349; occurrenceID: 7BB86EEF-0007-5714-8795-7B6AC37EDD4B; **Location:** locationID: V; decimalLatitude: 51.62022; decimalLongitude: -127.93070; **Event:** habitat: Saxicolous; **Record Level:** institutionID: CANL; collectionID: McMullin 19632
- **Type status:** Other material. **Occurrence:** catalogNumber: BOLD CALV108-20; recordedBy: R.T. McMullin; otherCatalogNumbers: GenBank OQ843208; occurrenceID: AA3DE571-0B9E-5351-9D82-046657339547; **Location:** locationID: VII; decimalLatitude: 51.61615; decimalLongitude: -127.93851; **Event:** habitat: Saxicolous; **Record Level:** institutionID: CANL; collectionID: McMullin 19643
- **Type status:** Other material. **Occurrence:** catalogNumber: BOLD PHAK362-20; recordedBy: A. Simon; otherCatalogNumbers: GenBank OQ922962; occurrenceID: 35B54FA3-F60A-5FE5-AF76-601DFA457D23; **Location:** locationID: X; decimalLatitude: 51.61977; decimalLongitude: -127.93245; **Record Level:** institutionID: UBC; collectionID: Simon 837

#### Pilophorus clavatus

Th. Fr.

01FA6C66-CB05-5A76-B323-8EF4DD65DA51

##### Materials

- **Type status:** Other material. **Occurrence:** catalogNumber: BOLD CALV109-20; recordedBy: R.T. McMullin; otherCatalogNumbers: GenBank OQ843322; occurrenceID: 3BE0892C-1655-52B0-AEEA-F98227D212A5; **Location:** locationID: VII; decimalLatitude: 51.61615; decimalLongitude: -127.93851; **Event:** habitat: Saxicolous; **Record Level:** institutionID: CANL; collectionID: McMullin 19658

#### Placopsis lambii

Hertel & V. Wirth

FD5B2CFD-32F2-569F-9CD8-BD59064924A0

##### Materials

- **Type status:** Other material. **Occurrence:** recordedBy: R.T. McMullin; occurrenceID: AE20719B-EB51-50AF-8B58-C1C0469E62ED; **Location:** locationID: V; decimalLatitude: 51.62022; decimalLongitude: -127.93070; **Identification:** identificationRemarks: Figure 4B. TLC: 5-0-methylhiasic and gyrophoric acids; **Event:** habitat: Saxicolous; **Record Level:** institutionID: CANL; collectionID: McMullin 19667
- **Type status:** Other material. **Occurrence:** recordedBy: A. Simon; occurrenceID: B3F6B3A2-58E8-5337-A19A-8456AE2D30EB; **Location:** locationID: IX; decimalLatitude: 51.61638; decimalLongitude: -127.94035; **Record Level:** institutionID: UBC; collectionID: Simon 838

#### Platismatia glauca

(L.) W.L. Culb. & C.F. Culb.

7D32DA27-1DB0-567B-9424-BB9EC1D19392

##### Materials

- **Type status:** Other material. **Occurrence:** recordedBy: R.T. McMullin; occurrenceID: 2E994D22-859B-5B6C-B6C9-FD386064F531; **Location:** locationID: XI; decimalLatitude: 51.61622; decimalLongitude: -127.94227; **Event:** habitat: Corticolous on *Alnusrubra*; **Record Level:** institutionID: CANL; collectionID: McMullin 19609
- **Type status:** Other material. **Occurrence:** catalogNumber: BOLD PHAK316-20; recordedBy: A. Simon; otherCatalogNumbers: GenBank OQ922976; occurrenceID: BDB1E005-8095-5046-AE6E-EA3E33404437; **Location:** locationID: XII; decimalLatitude: 51.66040; decimalLongitude: -128.11688; **Record Level:** institutionID: UBC; collectionID: Simon 799

#### Platismatia herrei

(Imshaug) W.L. Culb. & C.F. Culb.

662D7AED-BC4A-5FA7-8F84-A4EF37F0A8C2

##### Materials

- **Type status:** Other material. **Occurrence:** recordedBy: R.T. McMullin; occurrenceID: 782093DD-2C56-5ED3-91A5-FE184E1672B7; **Location:** locationID: III; decimalLatitude: 51.65486; decimalLongitude: -128.13907; **Event:** habitat: Corticolous on *Alnusrubra*; **Record Level:** institutionID: CANL; collectionID: McMullin 19550
- **Type status:** Other material. **Occurrence:** recordedBy: R.T. McMullin; occurrenceID: C1B7ECE1-933A-5667-A101-398DDE265F9A; **Location:** locationID: XX; decimalLatitude: 51.64809; decimalLongitude: -128.14378; **Event:** habitat: Corticolous on Pinuscontortassp.contorta; **Record Level:** institutionID: CANL; collectionID: McMullin 19566
- **Type status:** Other material. **Occurrence:** recordedBy: R.T. McMullin; occurrenceID: 58EC83DA-178A-5DFF-B54E-368A8E04BBA9; **Location:** locationID: V; decimalLatitude: 51.62022; decimalLongitude: -127.93070; **Event:** habitat: Corticolous on *Piceasitchensis*; **Record Level:** institutionID: CANL; collectionID: McMullin 19595
- **Type status:** Other material. **Occurrence:** recordedBy: R.T. McMullin; occurrenceID: 774B35CE-6393-55E8-8D1B-4EAA8FDBA48C; **Location:** locationID: XII; decimalLatitude: 51.66040; decimalLongitude: -128.11688; **Event:** habitat: Corticolous on Pinuscontortassp.contorta; **Record Level:** institutionID: CANL; collectionID: McMullin 19707
- **Type status:** Other material. **Occurrence:** catalogNumber: BOLD PHAK304-20; recordedBy: A. Simon; otherCatalogNumbers: GenBank OQ922991; occurrenceID: B1F51667-043B-5A1B-81F5-8379E1B31B25; **Location:** locationID: XXIX; decimalLatitude: 51.65271; decimalLongitude: -128.12951; **Record Level:** institutionID: UBC; collectionID: Simon 758

#### Platismatia lacunosa

(Ach.) W.L. Culb. & C.F. Culb.

D95E7C7F-631D-53BE-9E0F-7C1EE84F4E23

##### Materials

- **Type status:** Other material. **Occurrence:** catalogNumber: BOLD CALV111-20; recordedBy: R.T. McMullin; otherCatalogNumbers: GenBank OQ843239; occurrenceID: 16500457-E401-5366-BC71-9C37D543CD31; **Location:** locationID: XX; decimalLatitude: 51.64809; decimalLongitude: -128.14378; **Event:** habitat: Saxicolous; **Record Level:** institutionID: CANL; collectionID: McMullin 19564
- **Type status:** Other material. **Occurrence:** recordedBy: A. Simon; occurrenceID: 80FEA0FD-5E38-5BF4-B6BE-641ADF7ECCF4; **Location:** locationID: XXVIII; decimalLatitude: 51.66425; decimalLongitude: -128.12706; **Record Level:** institutionID: UBC; collectionID: Simon 759

#### Platismatia norvegica

(Lynge) W.L. Culb. & C.F. Culb.

50DBF4F7-3F71-5B36-BCA5-FAC269DF605F

##### Materials

- **Type status:** Other material. **Occurrence:** recordedBy: R.T. McMullin; occurrenceID: 37706F42-484D-5F35-9754-7023F625D624; **Location:** locationID: XIII; decimalLatitude: 51.66854; decimalLongitude: -128.11832; **Event:** habitat: Corticolous on *Alnusrubra*; **Record Level:** institutionID: CANL; collectionID: McMullin 19570
- **Type status:** Other material. **Occurrence:** recordedBy: R.T. McMullin; occurrenceID: 26FA83F1-5AAD-59D4-B46F-D07015A640B8; **Location:** locationID: V; decimalLatitude: 51.62022; decimalLongitude: -127.93070; **Event:** habitat: Corticolous on *Piceasitchensis*; **Record Level:** institutionID: CANL; collectionID: McMullin 19655
- **Type status:** Other material. **Occurrence:** recordedBy: R.T. McMullin; occurrenceID: F63EF47B-722A-5A74-9C65-749FB322506D; **Location:** locationID: V; decimalLatitude: 51.62022; decimalLongitude: -127.93070; **Event:** habitat: Corticolous; **Record Level:** institutionID: CANL; collectionID: McMullin 19681

#### Plectocarpon sp.

B8AE8459-A5BD-5216-B345-DBDC1278C5DA

##### Materials

- **Type status:** Other material. **Occurrence:** recordedBy: R.T. McMullin; occurrenceID: A24A2792-5B3F-57D3-91DD-C7CF979CF3FA; **Location:** locationID: XXI; decimalLatitude: 51.64221; decimalLongitude: -128.15085; **Event:** habitat: Lichenicolous on *Lobarialinita* on *Tsuga*; **Record Level:** institutionID: CANL; collectionID: McMullin 19535

##### Parasite of

*Lobarialinita*

##### Notes

Non-lichenised fungus. This specimen’s morphology and host correspond with *Plectocarpon* (Ertz et al. 2005), but no ascospores were seen to confirm its identity.

#### Polycauliona flavogranulosa

(Arup) Arup, Frödén & Søchting

03B4553E-77D3-5387-BC8C-DAE28BCD985C

##### Materials

- **Type status:** Other material. **Occurrence:** catalogNumber: BOLD PHAK353-20; recordedBy: A. Simon; otherCatalogNumbers: GenBank OQ922967; occurrenceID: A36882B0-D90D-5CB0-9707-C1C52B105CD2; **Location:** locationID: V; decimalLatitude: 51.62022; decimalLongitude: -127.93070; **Record Level:** institutionID: UBC; collectionID: Simon 818

#### Polycauliona sp.

1A0A9490-8CE6-546B-A2EE-12D49E1E08AA

##### Materials

- **Type status:** Other material. **Occurrence:** recordedBy: R.T. McMullin; occurrenceID: B6ED6743-9B56-5C36-9B9A-90CD92240CD1; **Location:** locationID: XII; decimalLatitude: 51.66040; decimalLongitude: -128.11688; **Event:** habitat: Saxicolous; **Record Level:** institutionID: CANL; collectionID: McMullin 19650
- **Type status:** Other material. **Occurrence:** recordedBy: R.T. McMullin; occurrenceID: D9A25924-C6A2-51B7-9F4F-6E0D2DAC988C; **Location:** locationID: XII; decimalLatitude: 51.66040; decimalLongitude: -128.11688; **Identification:** identificationRemarks: Figure 4C; **Event:** habitat: Saxicolous; **Record Level:** institutionID: CANL; collectionID: McMullin 19716

##### Notes

This taxon appears to be consistent with *Polycauliona* sp. S39572 in Spribille et al. (2020). Its bright orange thallus and elongated lobes are morphologically distinct and its habitat in the supralittoral zone is ecologically distinct. Appears to be a novel taxon.

#### Porina pacifica

Brodo

EAFB0653-E9DF-5DF1-90C6-61166ED0158B

##### Materials

- **Type status:** Other material. **Occurrence:** recordedBy: R.T. McMullin; occurrenceID: 582EEE15-8EC6-5F4E-91CC-FA32F67D3B7A; **Location:** locationID: V; decimalLatitude: 51.62022; decimalLongitude: -127.93070; **Event:** habitat: Saxicolous; **Record Level:** institutionID: CANL; collectionID: McMullin 19672

#### Protomicarea limosa

(Ach.) Hafellner

3759F7FE-45DB-5F3B-9DB9-0DF1BD896EEC

##### Materials

- **Type status:** Other material. **Occurrence:** catalogNumber: BOLD CALV187-20; recordedBy: R.T. McMullin; otherCatalogNumbers: GenBank OQ843251; occurrenceID: 61DBD3B9-61DE-5799-84FC-6C3015D1A988; **Location:** locationID: X; decimalLatitude: 51.61977; decimalLongitude: -127.93245; **Identification:** identificationRemarks: Figure 4D. TLC: no substance detected; **Event:** habitat: Bryicolous on rock; **Record Level:** institutionID: CANL; collectionID: McMullin 19698

#### Protopannaria pezizoides

(Weber) P.M. Jørg. & S. Ekman

B591309F-58CE-5B76-8BAA-30A34C1AD369

##### Materials

- **Type status:** Other material. **Occurrence:** recordedBy: R.T. McMullin; occurrenceID: 8AACC14C-3CCD-592A-A0FD-877AD6E97A0F; **Location:** locationID: V; decimalLatitude: 51.62022; decimalLongitude: -127.93070; **Event:** habitat: Saxicolous; **Record Level:** institutionID: CANL; collectionID: McMullin 19668

#### Pseudephebe pubescens

(L.) M. Choisy

7B496CAD-9383-5E70-A79C-22A78780BCA8

##### Materials

- **Type status:** Other material. **Occurrence:** recordedBy: Schofield; occurrenceID: 14E8277C-D0FB-5142-8566-9B9C1D0575BE; **Location:** locationID: Tundra on Mt. Buxton; **Record Level:** institutionID: UBC; collectionID: Schofield 28005
- **Type status:** Other material. **Occurrence:** recordedBy: Schofield; occurrenceID: 66864BAD-C6F3-5C4F-A70F-F370082ABAAB; **Location:** locationID: Tundra on Mt. Buxton; **Record Level:** institutionID: UBC; collectionID: Schofield 28009

##### Notes

Collected on Calvert Island prior to our survey, but we did not locate it.

#### Pseudocyphellaria citrina

(Gyeln.) Lücking, Moncada & S. Stenroos

503FA02F-2E16-5BAA-9CD6-4A6C431D649A

##### Materials

- **Type status:** Other material. **Occurrence:** catalogNumber: BOLD CALV114-20; recordedBy: R.T. McMullin; otherCatalogNumbers: GenBank OQ843361; occurrenceID: 231A893A-9A9A-5806-AA53-D14489A527F4; **Location:** locationID: XXI; decimalLatitude: 51.64221; decimalLongitude: -128.15085; **Event:** habitat: Corticolous on *Piceasitchensis*; **Record Level:** institutionID: CANL; collectionID: McMullin 19547
- **Type status:** Other material. **Occurrence:** catalogNumber: BOLD PHAK322-20; recordedBy: A. Simon; otherCatalogNumbers: GenBank OQ922979; occurrenceID: 0D19EE1E-BCE8-575C-A158-40C8F059A4EC; **Location:** locationID: XXI; decimalLatitude: 51.64221; decimalLongitude: -128.15085; **Record Level:** institutionID: UBC; collectionID: Simon 814

#### Pseudocyphellaria hawaiiensis

H. Magn.

0D903E74-FBAE-58CE-A03E-B31424CDB801

##### Materials

- **Type status:** Other material. **Occurrence:** catalogNumber: BOLD PHAK314-20; recordedBy: A. Simon; otherCatalogNumbers: GenBank OQ922975; occurrenceID: 5D150507-F97F-55E0-920F-01D394CFC11D; **Location:** locationID: XIII; decimalLatitude: 51.66854; decimalLongitude: -128.11832; **Record Level:** institutionID: UBC; collectionID: Simon 800

#### Pseudocyphellaria rainierensis

Imshaug

401E4862-4AAD-5C57-B1C1-DCC4AA8FD612

##### Materials

- **Type status:** Other material. **Occurrence:** catalogNumber: BOLD CALV039-20; recordedBy: R.T. McMullin; otherCatalogNumbers: GenBank OQ843297; occurrenceID: D9721117-53AA-53C6-B5AC-47003EE24930; **Location:** locationID: XIII; decimalLatitude: 51.66854; decimalLongitude: -128.11832; **Identification:** identificationRemarks: Figure 4E; **Event:** habitat: Corticolous on *Piceasitchensis*; **Record Level:** institutionID: CANL; collectionID: McMullin 19606

##### Notes

Federally listed as Special Concern on the Species at Risk Act (Government of Canada 2023).

#### Psoroma hypnorum

(Vahl) Gray

0AB4D34A-19BE-53B7-B4D5-8305090C0E14

##### Materials

- **Type status:** Other material. **Occurrence:** catalogNumber: BOLD CALV115-20; recordedBy: R.T. McMullin; otherCatalogNumbers: GenBank OQ843330; occurrenceID: FB6AEADD-B8D5-5B61-A74B-B9B591A826C2; **Location:** locationID: XXVI; decimalLatitude: 51.65466; decimalLongitude: -128.13051; **Identification:** identificationRemarks: Figure 4F; **Event:** habitat: Corticolous on the base of *Piceasitchensis*; **Record Level:** institutionID: CANL; collectionID: McMullin 19719

#### Pyrenula occidentalis

(R.C. Harris) R.C. Harris

5058CAAF-FE0A-5A74-826C-BF4C039016D5

##### Materials

- **Type status:** Other material. **Occurrence:** recordedBy: R.T. McMullin; occurrenceID: 7BFA14D1-29A5-57A9-A6F5-F4566C7F01A6; **Location:** locationID: XXI; decimalLatitude: 51.64221; decimalLongitude: -128.15085; **Event:** habitat: Corticolous on *Alnusrubra*; **Record Level:** institutionID: CANL; collectionID: McMullin 19548
- **Type status:** Other material. **Occurrence:** recordedBy: R.T. McMullin; occurrenceID: 46BB48E6-F593-5F62-9A63-28A778BA49F7; **Location:** locationID: X; decimalLatitude: 51.61977; decimalLongitude: -127.93245; **Event:** habitat: Corticolous on *Alnusrubra*; **Record Level:** institutionID: CANL; collectionID: McMullin 19699

#### Raesaenenia huuskonenii

(Räsänen) D. Hawksw., Boluda & H. Lindgr.

B095AE1E-B54B-53D4-ACCD-7259962FC368

##### Materials

- **Type status:** Other material. **Occurrence:** recordedBy: R.T. McMullin; occurrenceID: 6806267B-DADE-5085-9E9E-64E364D47517; **Location:** locationID: XII; decimalLatitude: 51.66040; decimalLongitude: -128.11688; **Event:** habitat: Lichenicolous on *Bryoriakockiana* on *Tsuga*; **Record Level:** institutionID: CANL; collectionID: McMullin 19878

##### Parasite of

*Bryoriakockiana*

##### Notes

Non-lichenised fungus.

#### Ramalina farinacea

(L.) Ach.

1DE3541A-F08F-5B85-AA1B-D7E7954AC139

##### Materials

- **Type status:** Other material. **Occurrence:** catalogNumber: BOLD CALV118-20; recordedBy: R.T. McMullin; otherCatalogNumbers: GenBank OQ843256; occurrenceID: 271CE6DC-C428-5E7F-BCF7-1A0E88778179; **Location:** locationID: III; decimalLatitude: 51.65486; decimalLongitude: -128.13907; **Event:** habitat: Corticolous on *Alnusrubra*; **Record Level:** institutionID: CANL; collectionID: McMullin 19549
- **Type status:** Other material. **Occurrence:** catalogNumber: BOLD CALV119-20; recordedBy: R.T. McMullin; otherCatalogNumbers: GenBank OQ843310; occurrenceID: C1D6D694-19CA-5BA8-8838-C25720471FCF; **Location:** locationID: III; decimalLatitude: 51.65486; decimalLongitude: -128.13907; **Event:** habitat: Corticolous on *Piceasitchensis*; **Record Level:** institutionID: CANL; collectionID: McMullin 19756
- **Type status:** Other material. **Occurrence:** catalogNumber: BOLD PHAK368-20; recordedBy: A. Simon; otherCatalogNumbers: GenBank OQ922943; occurrenceID: 97F57BD5-BFF1-5432-AB13-3563B8F864A0; **Location:** locationID: XXI; decimalLatitude: 51.64221; decimalLongitude: -128.15085; **Record Level:** institutionID: UBC; collectionID: Simon 850

#### Ramalina menziesii

Taylor

E2E99AA5-8243-5288-8D50-25AD939E7193

##### Materials

- **Type status:** Other material. **Occurrence:** catalogNumber: BOLD CALV120-20; recordedBy: R.T. McMullin; otherCatalogNumbers: GenBank OQ843298; occurrenceID: 0AC810A7-7770-5741-AEDB-1D3138AB4367; **Location:** locationID: XXIII; decimalLatitude: 51.64058; decimalLongitude: -128.14882; **Event:** habitat: Corticolous on a conifer snag; **Record Level:** institutionID: CANL; collectionID: McMullin 19693
- **Type status:** Other material. **Occurrence:** catalogNumber: BOLD PHAK377-20; recordedBy: A. Simon; otherCatalogNumbers: GenBank OQ922987; occurrenceID: 044A73F0-E668-55C7-97F7-3A23057749CF; **Location:** locationID: XXIII; decimalLatitude: 51.64058; decimalLongitude: -128.14882; **Record Level:** institutionID: UBC; collectionID: Simon 851

#### Ramalina roesleri

(Hochst. ex Schaer.) Hue

E12890A6-F181-5241-ADB8-97FECDEAE665

##### Materials

- **Type status:** Other material. **Occurrence:** recordedBy: R.T. McMullin; occurrenceID: 986B0C95-96C4-531C-9F06-4870DB29D832; **Location:** locationID: III; decimalLatitude: 51.65486; decimalLongitude: -128.13907; **Event:** habitat: Corticolous on *Piceasitchensis*; **Record Level:** institutionID: CANL; collectionID: McMullin 19754
- **Type status:** Other material. **Occurrence:** recordedBy: R.T. McMullin; occurrenceID: 746D417D-F039-50AF-9610-47E1FDA2347D; **Location:** locationID: II; decimalLatitude: 51.65285; decimalLongitude: -128.13873; **Event:** habitat: Corticolous on *Piceasitchensis*; **Record Level:** institutionID: CANL; collectionID: McMullin 19767
- **Type status:** Other material. **Occurrence:** recordedBy: A. Simon; occurrenceID: 3247863C-B223-5529-B7DE-4F547121497B; **Location:** locationID: XIV; decimalLatitude: 51.66797; decimalLongitude: -128.12128; **Record Level:** institutionID: UBC; collectionID: Simon 801
- **Type status:** Other material. **Occurrence:** catalogNumber: BOLD PHAK361-20; recordedBy: A. Simon; otherCatalogNumbers: GenBank OQ922968; occurrenceID: 6281FBD7-B01D-5EC9-A6C9-CB7EF5877509; **Location:** locationID: XXI; decimalLatitude: 51.64221; decimalLongitude: -128.15085; **Record Level:** institutionID: UBC; collectionID: Simon 852

#### Rhizocarpon anaperum

(Vain.) Vain.

A1B504A0-FEC5-5D1C-9755-7B47B6EDBBB2

##### Materials

- **Type status:** Other material. **Occurrence:** recordedBy: R.T. McMullin; occurrenceID: AC216E7D-9FF8-5C48-883F-068BCDBE5811; **Location:** locationID: II; decimalLatitude: 51.65285; decimalLongitude: -128.13873; **Event:** habitat: Saxicolous; **Record Level:** institutionID: CANL; collectionID: McMullin 19758

#### Rhizocarpon riparium

Räsänen

0F098082-0630-516C-8540-A34E634CD6A8

##### Materials

- **Type status:** Other material. **Occurrence:** recordedBy: R.T. McMullin; occurrenceID: 350A14D1-FAD9-5944-8810-E43D7F13540A; **Location:** locationID: XX; decimalLatitude: 51.64809; decimalLongitude: -128.14378; **Event:** habitat: Saxicolous; **Record Level:** institutionID: CANL; collectionID: McMullin 19534

#### Rinodina macrospora

Sheard

A6793A14-EC7B-5A7B-B60D-811988B8217F

##### Materials

- **Type status:** Other material. **Occurrence:** recordedBy: A. Simon; occurrenceID: F67BB738-180C-57B5-BC08-46D0C1B8195A; **Location:** locationID: XXI; decimalLatitude: 51.64221; decimalLongitude: -128.15085; **Record Level:** institutionID: UBC; collectionID: Simon 846

#### Sarea difformis

(Fr.) Fr.

D1C8955F-BEA8-54FF-8DCC-BB84CA071658

##### Materials

- **Type status:** Other material. **Occurrence:** recordedBy: R.T. McMullin; occurrenceID: 883A980B-9DE3-5E5B-993F-77282553D7F5; **Location:** locationID: I; decimalLatitude: 51.65501; decimalLongitude: -128.13799; **Event:** habitat: Resinicolous; **Record Level:** institutionID: CANL; collectionID: McMullin 19801
- **Type status:** Other material. **Occurrence:** recordedBy: R.T. McMullin; occurrenceID: DC7B9614-C6DE-5604-B416-FE682111614B; **Location:** locationID: VI; decimalLatitude: 51.61978; decimalLongitude: -127.93576; **Event:** habitat: Resinicolous on *Picea* resin; **Record Level:** institutionID: CANL; collectionID: McMullin 19803

##### Notes

Non-lichenised fungi. Purple pigment in hymenium KOH+ blue.

#### Scytinium gelatinosum

(With.) Otálora, P.M. Jørg. & Wedin

022645C1-0BE1-5181-9556-AAE764A1CBA8

##### Materials

- **Type status:** Other material. **Occurrence:** recordedBy: R.T. McMullin; occurrenceID: F91645EE-3793-5CEC-B11F-0FC491D53D41; **Location:** locationID: XV; decimalLatitude: 51.65616; decimalLongitude: -128.14052; **Event:** habitat: Terricolous on a rock; **Record Level:** institutionID: CANL; collectionID: McMullin 19711

#### Scytinium tenuissimum

(Dicks.) Otálora, P.M. Jørg. & Wedin

1E6B630D-13EC-5186-B4DA-C332F9346678

##### Materials

- **Type status:** Other material. **Occurrence:** recordedBy: R.T. McMullin; occurrenceID: 14F1AF56-3AAE-5321-A691-C3AFF4501BA0; **Location:** locationID: VI; decimalLatitude: 51.61978; decimalLongitude: -127.93576; **Event:** habitat: Terricolous on a rock; **Record Level:** institutionID: CANL; collectionID: McMullin 19686

#### Siphula ceratites

(Wahlenb.) Fr.

554F1063-2EEA-5119-8DB7-857A7E3C4E5D

##### Materials

**Figure 5.**
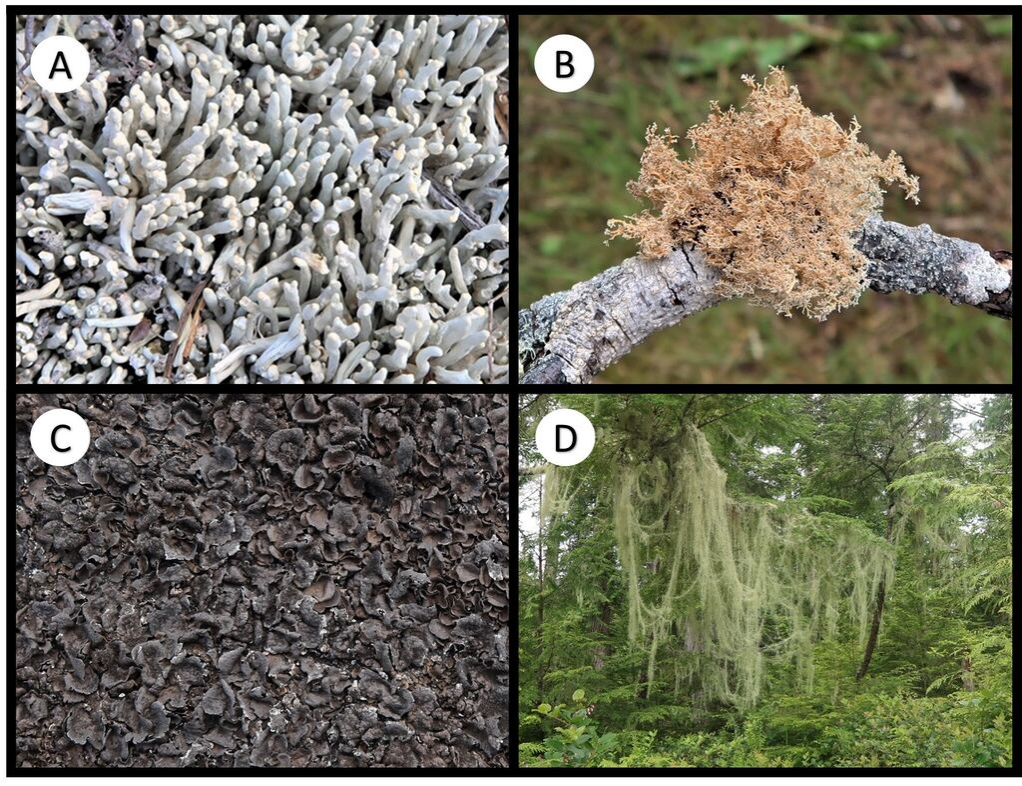
Lichens of Calvert Island. **A.** *Siphulaceratites*, *McMullin 19869* (CANL). **B.** *Sphaerophorustuckermanii*, *McMullin 19613* (CANL). **C.** *Umbilicariadeusta*, *McMullin 19519* (CANL). **D.** *Usnealongissima*, *McMullin 19540* (CANL).

- **Type status:** Other material. **Occurrence:** catalogNumber: BOLD CALV156-20; recordedBy: R.T. McMullin; otherCatalogNumbers: GenBank OQ843320; occurrenceID: FA0D3119-DDBA-5AA2-A731-A9C6971B8298; **Location:** locationID: XX; decimalLatitude: 51.64809; decimalLongitude: -128.14378; **Event:** habitat: Terricolous; **Record Level:** institutionID: CANL; collectionID: McMullin 19544
- **Type status:** Other material. **Occurrence:** recordedBy: R.T. McMullin; occurrenceID: D456B116-32EF-51D4-BED7-9B1B46DF26D8; **Location:** locationID: XI; decimalLatitude: 51.61622; decimalLongitude: -127.94227; **Event:** habitat: Terricolous; **Record Level:** institutionID: CANL; collectionID: McMullin 19636
- **Type status:** Other material. **Occurrence:** recordedBy: R.T. McMullin; occurrenceID: 9471FC6A-9AB6-5E36-95B7-6BB166342E5C; **Location:** locationID: XXV; decimalLatitude: 51.65180; decimalLongitude: -128.12865; **Identification:** identificationRemarks: Figure 5A; **Event:** habitat: Terricolous; **Record Level:** institutionID: CANL; collectionID: McMullin 19869
- **Type status:** Other material. **Occurrence:** catalogNumber: BOLD PHAK291-20; recordedBy: A. Simon; otherCatalogNumbers: GenBank OQ922958; occurrenceID: 4D82BEA8-E3E6-5DA2-AF0C-2F2E30F189C9; **Location:** locationID: XXV; decimalLatitude: 51.65180; decimalLongitude: -128.12865; **Record Level:** institutionID: UBC; collectionID: Simon 771

#### Sphaerophorus fragilis

(L.) Pers.

C64E6D58-C9F6-5BE7-ACA8-6EB8EDAC422E

##### Materials

- **Type status:** Other material. **Occurrence:** catalogNumber: BOLD PHAK306-20; recordedBy: A. Simon; otherCatalogNumbers: GenBank OQ922982; occurrenceID: CC5A6FF1-7D96-56DC-AA03-4D82B86C3E51; **Location:** locationID: XXX; decimalLatitude: 51.63722; decimalLongitude: -128.09525; **Record Level:** institutionID: UBC; collectionID: Simon 772

#### Sphaerophorus tuckermanii

Räsänen

00D21D6B-6CB5-51B2-AD65-DAF44F795E6E

##### Materials

- **Type status:** Other material. **Occurrence:** recordedBy: R.T. McMullin; occurrenceID: 5C2F5C20-A99F-515D-9768-3BF9B18EC809; **Location:** locationID: XX; decimalLatitude: 51.64809; decimalLongitude: -128.14378; **Event:** habitat: Corticolous on Pinuscontortassp.contorta; **Record Level:** institutionID: CANL; collectionID: McMullin 19511
- **Type status:** Other material. **Occurrence:** recordedBy: R.T. McMullin; occurrenceID: 05172828-8B5B-520F-AFE9-E3CF4F017250; **Location:** locationID: IV; decimalLatitude: 51.65514; decimalLongitude: -128.13243; **Event:** habitat: Corticolous on *Tsuga*; **Record Level:** institutionID: CANL; collectionID: McMullin 19525
- **Type status:** Other material. **Occurrence:** recordedBy: R.T. McMullin; occurrenceID: E24C1C91-E2E3-544A-B889-9EA6401455A7; **Location:** locationID: XIII; decimalLatitude: 51.66854; decimalLongitude: -128.11832; **Identification:** identificationRemarks: Figure 5B; **Event:** habitat: Corticolous on a snag; **Record Level:** institutionID: CANL; collectionID: McMullin 19613
- **Type status:** Other material. **Occurrence:** recordedBy: R.T. McMullin; occurrenceID: 0AF53CD6-91DC-553F-9AB1-70735D4FAA40; **Location:** locationID: V; decimalLatitude: 51.62022; decimalLongitude: -127.93070; **Event:** habitat: Corticolous on *Alnusrubra*; **Record Level:** institutionID: CANL; collectionID: McMullin 19684
- **Type status:** Other material. **Occurrence:** recordedBy: R.T. McMullin; occurrenceID: 7121BFD6-8538-510F-B0A9-5C55024A35D1; **Location:** locationID: II; decimalLatitude: 51.65285; decimalLongitude: -128.13873; **Event:** habitat: Terricolous; **Record Level:** institutionID: CANL; collectionID: McMullin 19760
- **Type status:** Other material. **Occurrence:** recordedBy: R.T. McMullin; occurrenceID: 281D746D-A825-5B4E-A0F3-3B77320AD8F2; **Location:** locationID: XVIII; decimalLatitude: 51.66476; decimalLongitude: -128.11798; **Event:** habitat: Corticolous on *Piceasitchensis*; **Record Level:** institutionID: CANL; collectionID: McMullin 19768
- **Type status:** Other material. **Occurrence:** catalogNumber: BOLD PHAK297-20; recordedBy: A. Simon; otherCatalogNumbers: GenBank OQ922936; occurrenceID: 72C14FE7-6DB9-5399-B197-53D259BFFD4C; **Location:** locationID: XXIX; decimalLatitude: 51.65271; decimalLongitude: -128.12951; **Record Level:** institutionID: UBC; collectionID: Simon 760

#### Spilonema maritimum

T. Sprib. & Fryday

7125A70D-2C1C-5B10-8B30-4FB12CB08D2D

##### Materials

- **Type status:** Other material. **Occurrence:** recordedBy: R.T. McMullin; occurrenceID: B0BF2961-3BF2-58BE-9ACE-87548E4DCD2C; **Location:** locationID: V; decimalLatitude: 51.62022; decimalLongitude: -127.93070; **Event:** habitat: Saxicolous; **Record Level:** institutionID: CANL; collectionID: McMullin 19717

##### Notes

This newly-described species (Spribille et al. 2020) was previously known from British Columbia (e.g. Benton et al. (1977), Noble et al. (1987), Brodo and Sloan (2005)), but reported as *S.revertens* Nyl. The latter species, however, prefers interior habitats that are mainly dry and forms small cushions, lacks marginal lobes and is often associated with *Psorularufonigra* (Tuck.) Gotth. Schneid. (Brodo et al. 2001). *Spilonemamaritimum* is a common maritime lichen growing on coastal rocks in the middle supralittoral zone (Brodo and Sloan 2005).

#### Steineropsis laceratula

(Hue) T. Sprib. & S. Ekman

030C8325-1D81-58BA-B26F-23DDCBCBF119

##### Materials

- **Type status:** Other material. **Occurrence:** catalogNumber: BOLD CALV265-20; recordedBy: R.T. McMullin; otherCatalogNumbers: GenBank OQ843290; occurrenceID: B9F8BE89-C01B-58BF-9A4A-A47F9B697F0D; **Location:** locationID: III; decimalLatitude: 51.65486; decimalLongitude: -128.13907; **Identification:** identificationRemarks: TLC: atranorin; **Event:** habitat: Corticolous on *Alnusrubra*; **Record Level:** institutionID: CANL; collectionID: McMullin 19546
- **Type status:** Other material. **Occurrence:** catalogNumber: BOLD CALV270-20; recordedBy: R.T. McMullin; otherCatalogNumbers: GenBank OQ843336; occurrenceID: E3500A82-640C-5117-A40C-9A52CC4ADE2D; **Location:** locationID: III; decimalLatitude: 51.65486; decimalLongitude: -128.13907; **Event:** habitat: Corticolous on *Alnusrubra*; **Record Level:** institutionID: CANL; collectionID: McMullin 19556
- **Type status:** Other material. **Occurrence:** catalogNumber: BOLD CALV267-20; recordedBy: R.T. McMullin; otherCatalogNumbers: GenBank OQ843221; occurrenceID: 1A4ACA3F-6794-52DC-B524-9F007B0F373E; **Location:** locationID: II; decimalLatitude: 51.65285; decimalLongitude: -128.13873; **Identification:** identificationRemarks: TLC: atranorin; **Event:** habitat: Saxicolous; **Record Level:** institutionID: CANL; collectionID: McMullin 19771
- **Type status:** Other material. **Occurrence:** catalogNumber: BOLD CALV266-20; recordedBy: R.T. McMullin; otherCatalogNumbers: GenBank OQ843264; occurrenceID: 54A93869-E7DA-5B6B-A49F-71C53556A356; **Location:** locationID: XXII; decimalLatitude: 51.64502; decimalLongitude: -128.15099; **Identification:** identificationRemarks: TLC: atranorin; **Event:** habitat: Corticolous on *Thujaplicata*; **Record Level:** institutionID: CANL; collectionID: McMullin 19784
- **Type status:** Other material. **Occurrence:** recordedBy: R.T. McMullin; occurrenceID: BB81E642-5C2A-5C42-974D-51BEAC1D7DA0; **Location:** locationID: II; decimalLatitude: 51.65285; decimalLongitude: -128.13873; **Event:** habitat: Saxicolous; **Record Level:** institutionID: CANL; collectionID: McMullin 19853
- **Type status:** Other material. **Occurrence:** catalogNumber: BOLD PHAK298-20; recordedBy: A. Simon; otherCatalogNumbers: GenBank OQ922954; occurrenceID: DD5FC20A-69A0-5709-9094-D35CC2F4F853; **Location:** locationID: XXI; decimalLatitude: 51.64221; decimalLongitude: -128.15085; **Record Level:** institutionID: UBC; collectionID: Simon 803
- **Type status:** Other material. **Occurrence:** catalogNumber: BOLD PHAK321-20; recordedBy: A. Simon; otherCatalogNumbers: GenBank OQ922972; occurrenceID: E13C7780-0D8D-55C4-8EC1-AA041FC3A3BC; **Location:** locationID: XXI; decimalLatitude: 51.64221; decimalLongitude: -128.15085; **Record Level:** institutionID: UBC; collectionID: Simon 844
- **Type status:** Other material. **Occurrence:** catalogNumber: BOLD PHAK358-20; recordedBy: A. Simon; otherCatalogNumbers: GenBank OQ922947; occurrenceID: 4A7D5F2B-D841-5478-A2A2-C8F725512CD9; **Location:** locationID: XXI; decimalLatitude: 51.64221; decimalLongitude: -128.15085; **Record Level:** institutionID: UBC; collectionID: Simon 849

#### Stenocybe pullatula

(Ach.) Stein

3400B95C-5902-5CC4-BD8C-597A7EABD38F

##### Materials

- **Type status:** Other material. **Occurrence:** catalogNumber: BOLD CALV126-20; recordedBy: R.T. McMullin; otherCatalogNumbers: GenBank OQ843280; occurrenceID: AD71B46E-D095-53BF-99F2-AFDD3AD8DD06; **Location:** locationID: V; decimalLatitude: 51.62022; decimalLongitude: -127.93070; **Event:** habitat: Corticolous on *Alnusrubra*; **Record Level:** institutionID: CANL; collectionID: McMullin 19590

##### Notes

Non-lichenised fungus.

#### Stereocaulon sterile

(Savicz) I.M. Lamb ex Krog

7AA0BA7A-C3E0-53AD-857F-4D57711E063C

##### Materials

- **Type status:** Other material. **Occurrence:** recordedBy: R.T. McMullin; occurrenceID: 9E9B1237-4334-5AD5-AF5D-E2533B271F90; **Location:** locationID: XXIII; decimalLatitude: 51.64058; decimalLongitude: -128.14882; **Event:** habitat: Saxicolous; **Record Level:** institutionID: CANL; collectionID: McMullin 19694
- **Type status:** Other material. **Occurrence:** recordedBy: R.T. McMullin; occurrenceID: 0E42FD98-C0C0-597B-AE72-595564EFD856; **Location:** locationID: II; decimalLatitude: 51.65285; decimalLongitude: -128.13873; **Event:** habitat: Saxicolous; **Record Level:** institutionID: CANL; collectionID: McMullin 19759
- **Type status:** Other material. **Occurrence:** recordedBy: A. Simon; occurrenceID: FA98A3A2-4E97-5D83-AC10-B90F491846F9; **Location:** locationID: II; decimalLatitude: 51.65285; decimalLongitude: -128.13873; **Record Level:** institutionID: UBC; collectionID: Simon 761
- **Type status:** Other material. **Occurrence:** recordedBy: A. Simon; occurrenceID: 2B91CD60-07C2-50B7-A329-C7438A1B0E8A; **Location:** locationID: XXX; decimalLatitude: 51.63722; decimalLongitude: -128.09525; **Record Level:** institutionID: UBC; collectionID: Simon 778

#### Stereocaulon vesuvianum

Pers.

DE669E6B-0621-587E-ADFB-0068CFA904FF

##### Materials

- **Type status:** Other material. **Occurrence:** catalogNumber: BOLD PHAK305-20; recordedBy: A. Simon; otherCatalogNumbers: GenBank OQ922980; occurrenceID: 01FF93D5-EC1C-5D06-8706-B48C9C2F3545; **Location:** locationID: XXX; decimalLatitude: 51.63722; decimalLongitude: -128.09525; **Record Level:** institutionID: UBC; collectionID: Simon 773

#### Thelotrema lepadinum

(Ach.) Ach.

536157B5-F41B-567A-A7EC-6CC5881F166C

##### Materials

- **Type status:** Other material. **Occurrence:** catalogNumber: BOLD CALV163-20; recordedBy: R.T. McMullin; otherCatalogNumbers: GenBank OQ843350; occurrenceID: 2AF158D6-EF84-543B-953B-51B5CBEC916C; **Location:** locationID: IV; decimalLatitude: 51.65514; decimalLongitude: -128.13243; **Event:** habitat: Corticolous on *Alnusrubra*; **Record Level:** institutionID: CANL; collectionID: McMullin 19523
- **Type status:** Other material. **Occurrence:** recordedBy: R.T. McMullin; occurrenceID: 6232B8C2-BF5C-594C-853E-CA4FC2DE291F; **Location:** locationID: XVI; decimalLatitude: 51.66051; decimalLongitude: -128.14587; **Event:** habitat: Corticolous on *Alnusrubra*; **Record Level:** institutionID: CANL; collectionID: McMullin 19579
- **Type status:** Other material. **Occurrence:** recordedBy: R.T. McMullin; occurrenceID: AD3911C1-D441-587B-AA4E-07D71E84AB29; **Location:** locationID: XIII; decimalLatitude: 51.66854; decimalLongitude: -128.11832; **Event:** habitat: Corticolous on *Alnusrubra*; **Record Level:** institutionID: CANL; collectionID: McMullin 19596

#### Trapelia corticola

Coppins & P. James

F3C36C3D-EB8D-5D4B-B2CE-4CCD4D47B183

##### Materials

- **Type status:** Other material. **Occurrence:** catalogNumber: BOLD CALV131-20; recordedBy: R.T. McMullin; otherCatalogNumbers: GenBank OQ843352; occurrenceID: E53AA03D-9210-55F5-8E61-E3D5F8C664DF; **Location:** locationID: IV; decimalLatitude: 51.65514; decimalLongitude: -128.13243; **Event:** habitat: Corticolous on *Tsuga*; **Record Level:** institutionID: CANL; collectionID: McMullin 19522

##### Notes

With gyrophoric acid.

#### Trapeliopsis sp.

AF9AF2F1-D232-5005-8DA4-96CEC7FD5E9A

##### Materials

- **Type status:** Other material. **Occurrence:** catalogNumber: BOLD CALV132-20; recordedBy: R.T. McMullin; otherCatalogNumbers: GenBank OQ843205; occurrenceID: D37F8927-49EB-57F8-9698-9A7CF73A9E0C; **Location:** locationID: III; decimalLatitude: 51.65486; decimalLongitude: -128.13907; **Event:** habitat: Corticolous; **Record Level:** institutionID: CANL; collectionID: McMullin 19538
- **Type status:** Other material. **Occurrence:** catalogNumber: BOLD CALV133-20; recordedBy: R.T. McMullin; otherCatalogNumbers: GenBank OQ843301; occurrenceID: F54CA003-0065-53C0-B61F-EF1757796E10; **Location:** locationID: I; decimalLatitude: 51.65501; decimalLongitude: -128.13799; **Event:** habitat: Corticolous; **Record Level:** institutionID: CANL; collectionID: McMullin 19739

##### Notes

Thallus thin, green, granulose. Soredia fine to granular, in soralia or becoming confluent, pale green (paler than thallus). Apothecia absent. Resembles *Trapeliopsisviridescens* (Schrad.) Coppins & P. James. Appears to be a novel taxon.

#### Tuckermanopsis chlorophylla

(Willd.) Hale

2F31D0D6-249C-57A4-A0D6-46B6EAFC7E53

##### Materials

- **Type status:** Other material. **Occurrence:** catalogNumber: BOLD CALV134-20; recordedBy: R.T. McMullin; otherCatalogNumbers: GenBank OQ843279; occurrenceID: 2530E08E-552D-5662-AD4A-9BD058CE4EEB; **Location:** locationID: XII; decimalLatitude: 51.66040; decimalLongitude: -128.11688; **Event:** habitat: Corticolous on Pinuscontortassp.contorta; **Record Level:** institutionID: CANL; collectionID: McMullin 19577
- **Type status:** Other material. **Occurrence:** catalogNumber: BOLD PHAK373-20; recordedBy: A. Simon; otherCatalogNumbers: GenBank OQ922960; occurrenceID: 56F75EA4-A110-5972-B546-9C2BE10DC701; **Location:** locationID: X; decimalLatitude: 51.61977; decimalLongitude: -127.93245; **Record Level:** institutionID: UBC; collectionID: Simon 819
- **Type status:** Other material. **Occurrence:** catalogNumber: BOLD PHAK370-20; recordedBy: A. Simon; otherCatalogNumbers: GenBank OQ922985; occurrenceID: 7B7C259D-2E8A-5325-A08B-9C75F7EA5C05; **Location:** locationID: XII; decimalLatitude: 51.66040; decimalLongitude: -128.11688; **Record Level:** institutionID: UBC; collectionID: Simon 853

#### Umbilicaria angulata

Tuck.

FAFFB493-69F3-511C-8174-CB533B3E3C5F

##### Materials

- **Type status:** Other material. **Occurrence:** recordedBy: R.T. McMullin; occurrenceID: 4E38BDF2-9898-53FA-B2A9-D9BC1899338B; **Location:** locationID: XX; decimalLatitude: 51.64809; decimalLongitude: -128.14378; **Event:** habitat: Saxicolous; **Record Level:** institutionID: CANL; collectionID: McMullin 19565
- **Type status:** Other material. **Occurrence:** recordedBy: R.T. McMullin; occurrenceID: E53260D5-C5E3-544B-8D43-318C998819D9; **Location:** locationID: XIV; decimalLatitude: 51.66797; decimalLongitude: -128.12128; **Event:** habitat: Saxicolous; **Record Level:** institutionID: CANL; collectionID: McMullin 19583

#### Umbilicaria deusta

(L.) Baumg.

460E2849-30E3-5567-87DE-690CB024896B

##### Materials

- **Type status:** Other material. **Occurrence:** catalogNumber: BOLD CALV137-20; recordedBy: R.T. McMullin; otherCatalogNumbers: GenBank OQ843283; occurrenceID: 7C14843C-8CA5-5DDD-8E28-6B64E8853239; **Location:** locationID: XX; decimalLatitude: 51.64809; decimalLongitude: -128.14378; **Identification:** identificationRemarks: Figure 5C; **Event:** habitat: Saxicolous; **Record Level:** institutionID: CANL; collectionID: McMullin 19519

#### Umbilicaria hyperborea

(Ach.) Hoffm.

DC56DC32-A30F-54E3-B78B-0D58960B0E66

##### Materials

- **Type status:** Other material. **Occurrence:** recordedBy: Schofield; occurrenceID: 20A9AE7F-78B1-5E89-AA1F-8566D298CDEC; **Location:** locationID: Tundra on Mt. Buxton; **Record Level:** institutionID: UBC; collectionID: Schofield 27996

##### Notes

Collected on Calvert Island prior to our survey, but we did not locate it.

#### Umbilicaria polyphylla

(L.) Baumg.

88B5A288-1AE3-573F-9D8B-6246BC1DDBA7

##### Materials

- **Type status:** Other material. **Occurrence:** recordedBy: R.T. McMullin; occurrenceID: B8BD8AC1-C6E0-5DC8-959E-AF6F7E2BD217; **Location:** locationID: XX; decimalLatitude: 51.64809; decimalLongitude: -128.14378; **Event:** habitat: Saxicolous; **Record Level:** institutionID: CANL; collectionID: McMullin 19506
- **Type status:** Other material. **Occurrence:** recordedBy: A. Simon; occurrenceID: FCC39F7A-970A-541F-9EB3-34667CF5BC9F; **Location:** locationID: XXX; decimalLatitude: 51.63722; decimalLongitude: -128.09525; **Record Level:** institutionID: UBC; collectionID: Simon 777

#### Umbilicaria polyrrhiza

(L.) Fr.

50FEC7C5-9D3E-583E-8E85-50627C2D085A

##### Materials

- **Type status:** Other material. **Occurrence:** recordedBy: A. Simon; occurrenceID: 8C42DC30-D616-55F1-BEEC-FF6E5354F6C7; **Location:** locationID: XXX; decimalLatitude: 51.63722; decimalLongitude: -128.09525; **Record Level:** institutionID: UBC; collectionID: Simon 774
- **Type status:** Other material. **Occurrence:** recordedBy: A. Simon; occurrenceID: 83E2B4DA-5125-54BA-A879-2107AF456DB3; **Location:** locationID: XXX; decimalLatitude: 51.63722; decimalLongitude: -128.09525; **Record Level:** institutionID: UBC; collectionID: Simon 776

#### Umbilicaria torrefacta

(Lightf.) Schrad.

56C965A5-93B4-5112-A9D2-145F5DB357B0

##### Materials

- **Type status:** Other material. **Occurrence:** catalogNumber: BOLD PHAK292-20; recordedBy: A. Simon; otherCatalogNumbers: GenBank OQ922941; occurrenceID: 23103B1A-359A-5E39-BCCB-08736FF2D3B9; **Location:** locationID: XXX; decimalLatitude: 51.63722; decimalLongitude: -128.09525; **Record Level:** institutionID: UBC; collectionID: Simon 775

#### Usnea cornuta

Körb.

ACCD7358-83FB-572D-BC2D-F8CE444259CD

##### Materials

- **Type status:** Other material. **Occurrence:** recordedBy: R.T. McMullin; occurrenceID: FFEFDE98-DC5A-55EE-914D-E4BF9753F5E9; **Location:** locationID: XII; decimalLatitude: 51.66040; decimalLongitude: -128.11688; **Event:** habitat: Corticolous on Pinuscontortassp.contorta; **Record Level:** institutionID: CANL; collectionID: McMullin 19688
- **Type status:** Other material. **Occurrence:** recordedBy: R.T. McMullin; occurrenceID: F09685C2-7677-56FF-8F6C-C1F8F04275DA; **Location:** locationID: XIX; decimalLatitude: 51.65065; decimalLongitude: -128.14241; **Event:** habitat: Corticolous on *Thujaplicata*; **Record Level:** institutionID: CANL; collectionID: McMullin 19794
- **Type status:** Other material. **Occurrence:** recordedBy: A. Simon; occurrenceID: 1ABA4DD4-39E1-51C5-93D9-5691EC50BE7E; **Location:** locationID: XIX; decimalLatitude: 51.65065; decimalLongitude: -128.14241; **Record Level:** institutionID: UBC; collectionID: Simon 782
- **Type status:** Other material. **Occurrence:** catalogNumber: BOLD PHAK286-20; recordedBy: A. Simon; otherCatalogNumbers: GenBank OQ922973; occurrenceID: 78828C96-9C03-5FD9-890B-600D75913D18; **Location:** locationID: XIII; decimalLatitude: 51.66854; decimalLongitude: -128.11832; **Record Level:** institutionID: UBC; collectionID: Simon 802
- **Type status:** Other material. **Occurrence:** recordedBy: A. Simon; occurrenceID: 14694334-AD1B-5299-AF5F-5E897CCDE0DC; **Location:** locationID: XXIII; decimalLatitude: 51.64058; decimalLongitude: -128.14882; **Record Level:** institutionID: UBC; collectionID: Simon 855

#### Usnea dasopoga

(Ach.) Nyl.

10C86490-BEC4-50AF-811C-26089FE18886

##### Materials

- **Type status:** Other material. **Occurrence:** catalogNumber: BOLD CALV140-20; recordedBy: R.T. McMullin; otherCatalogNumbers: GenBank OQ843362; occurrenceID: BA15D382-1C10-54D4-84D2-4E860CB328C9; **Location:** locationID: XII; decimalLatitude: 51.66040; decimalLongitude: -128.11688; **Event:** habitat: Corticolous on Pinuscontortassp.contorta; **Record Level:** institutionID: CANL; collectionID: McMullin 19633
- **Type status:** Other material. **Occurrence:** recordedBy: A. Simon; occurrenceID: DA6F94CC-E0C6-52E9-A0DF-E236E72DCB82; **Location:** locationID: XXI; decimalLatitude: 51.64221; decimalLongitude: -128.15085; **Record Level:** institutionID: UBC; collectionID: Simon 816

#### Usnea fragilescens

Hav. ex Lynge

B7B794AF-E8D5-5504-8601-1D3CDF44239A

##### Materials

- **Type status:** Other material. **Occurrence:** recordedBy: R.T. McMullin; occurrenceID: 47B5158A-3B76-59B3-A6E7-1B86368F4D5F; **Location:** locationID: XIX; decimalLatitude: 51.65065; decimalLongitude: -128.14241; **Event:** habitat: Corticolous on *Thujaplicata*; **Record Level:** institutionID: CANL; collectionID: McMullin 19791

#### Usnea longissima

Ach.

AB9CA23E-C150-5A8B-89AF-857BD0814B01

##### Materials

- **Type status:** Other material. **Occurrence:** catalogNumber: BOLD CALV141-20; recordedBy: R.T. McMullin; otherCatalogNumbers: GenBank OQ843197; occurrenceID: 63CFF75A-30D3-5CD7-A428-74A810A9A8AC; **Location:** locationID: IV; decimalLatitude: 51.65514; decimalLongitude: -128.13243; **Identification:** identificationRemarks: Figure 5D; **Event:** habitat: Corticolous on *Tsuga*; **Record Level:** institutionID: CANL; collectionID: McMullin 19540
- **Type status:** Other material. **Occurrence:** catalogNumber: BOLD CALV142-20; recordedBy: R.T. McMullin; otherCatalogNumbers: GenBank OQ843348; occurrenceID: 54FDA17F-422A-5676-B531-509CE59450AE; **Location:** locationID: V; decimalLatitude: 51.62022; decimalLongitude: -127.93070; **Event:** habitat: Corticolous on *Piceasitchensis*; **Record Level:** institutionID: CANL; collectionID: McMullin 19572
- **Type status:** Other material. **Occurrence:** catalogNumber: BOLD CALV143-20; recordedBy: R.T. McMullin; otherCatalogNumbers: GenBank OQ843293; occurrenceID: 757745CC-4C8E-5E03-A5D9-982D1A7FDDA8; **Location:** locationID: XXVII; decimalLatitude: 51.65589; decimalLongitude: -128.13098; **Event:** habitat: Corticolous on *Alnusrubra*; **Record Level:** institutionID: CANL; collectionID: McMullin 19870
- **Type status:** Other material. **Occurrence:** recordedBy: A. Simon; occurrenceID: C52AB2EA-A488-5C56-BA7C-40BEACF1D1C4; **Location:** locationID: XXVIII; decimalLatitude: 51.66425; decimalLongitude: -128.12706; **Record Level:** institutionID: UBC; collectionID: Simon 762

#### Verrucaria schofieldii

Brodo

E3AA63A0-5D0F-5C2B-8F74-1AABCC9EFF05

##### Materials

- **Type status:** Other material. **Occurrence:** catalogNumber: BOLD CALV179-20; recordedBy: R.T. McMullin; otherCatalogNumbers: GenBank OQ843200; occurrenceID: 901E2BC0-8F75-5151-A195-16E7C7E1D06D; **Location:** locationID: V; decimalLatitude: 51.62022; decimalLongitude: -127.93070; **Event:** habitat: Saxicolous; **Record Level:** institutionID: CANL; collectionID: McMullin 19703
- **Type status:** Other material. **Occurrence:** recordedBy: R.T. McMullin; occurrenceID: 67BF0B1F-6C3B-5CD6-8739-4CD26E8546DD; **Location:** locationID: II; decimalLatitude: 51.65285; decimalLongitude: -128.13873; **Event:** habitat: Saxicolous; **Record Level:** institutionID: CANL; collectionID: McMullin 19783

#### Xylographa opegraphella

Nyl.

5FD7D81C-F887-5D75-ACB8-C28F4974803E

##### Materials

- **Type status:** Other material. **Occurrence:** catalogNumber: BOLD CALV186-20; recordedBy: R.T. McMullin; otherCatalogNumbers: GenBank OQ843357; occurrenceID: 6575C922-AF0C-5F14-987A-B01F742622FD; **Location:** locationID: XIII; decimalLatitude: 51.66854; decimalLongitude: -128.11832; **Identification:** identificationRemarks: TLC: stictic acid, trace of norstictic acid; **Event:** habitat: Lignicolous on a stump; **Record Level:** institutionID: CANL; collectionID: McMullin 19644

#### Xylographa sp.

5CFB4121-22AE-5023-B637-28B93D2F0393

##### Materials

- **Type status:** Other material. **Occurrence:** catalogNumber: BOLD CALV097-20; recordedBy: R.T. McMullin; otherCatalogNumbers: GenBank OQ843333; occurrenceID: 8F65EC89-B71D-54F0-92FA-0D2FE1DA64E2; **Location:** locationID: III; decimalLatitude: 51.65486; decimalLongitude: -128.13907; **Identification:** identificationRemarks: TLC: stictic acid; **Event:** habitat: Lignicolous on a log; **Record Level:** institutionID: CANL; collectionID: McMullin 19529
- **Type status:** Other material. **Occurrence:** catalogNumber: BOLD CALV145-20; recordedBy: R.T. McMullin; otherCatalogNumbers: GenBank OQ843344; occurrenceID: 936DA526-B39E-597C-AD94-393916F85822; **Location:** locationID: III; decimalLatitude: 51.65486; decimalLongitude: -128.13907; **Event:** habitat: Lignicolous on a log; **Record Level:** institutionID: CANL; collectionID: McMullin 19776

##### Notes

Thallus immersed, creating a whitish stain. Apothecia brown, margins paler than the disc, initially somewhat circular, becoming elongated, unbranched, tapering at both ends, 0.2–0.6 mm × 0.1–0.15 mm. Ascospores 8 per ascus, simple, hyaline, 10–12.5 μm × 6–7.5 μm. Appears to be a novel taxon.

#### Zythia resinae

(Ehrenb.) P. Karst

81307D58-A020-5016-B573-C010ACACED3A

##### Materials

- **Type status:** Other material. **Occurrence:** recordedBy: R.T. McMullin; occurrenceID: 0962F030-0AA6-5E37-9C51-08AE8FA75179; **Location:** locationID: I; decimalLatitude: 51.65501; decimalLongitude: -128.13799; **Event:** habitat: Resinicolous; **Record Level:** institutionID: CANL; collectionID: McMullin 19800
- **Type status:** Other material. **Occurrence:** recordedBy: R.T. McMullin; occurrenceID: 19E1F991-9647-540D-9783-2C85A13FDA47; **Location:** locationID: VI; decimalLatitude: 51.61978; decimalLongitude: -127.93576; **Event:** habitat: Resinicolous on *Picea* resin; **Record Level:** institutionID: CANL; collectionID: McMullin 19802

##### Notes

Non-lichenised fungus.

## Analysis

We identified 189 species in 85 genera of lichens and allied fungi from 449 specimens collected during our survey on Calvert Island. These include 71 (38%) microlichens (crustose species), 102 (54%) macrolichens (51 foliose and 51 fruticose) and 16 (8%) non-lichenised allied fungi. Cyanobacteria are the primary photobiont in 22 (13%) lichen species, while an additional 13 (8%) produce cephalodia and are, therefore, a tripartite symbiosis. In the remaining 138 lichen species (80%), the primary photobiont is green algae. Calicioids are represented by 16 (8%) species (including *Bunodophoron* and *Sphaerophorus*), half of which are non-lichenised. We generated ITS sequences from 215 specimens representing 121 species.

We collected three species for the first time from British Columbia, *Bryoriafurcellata*, *Chaenothecopsislecanactidis* and *C.nigripunctata*, which were reported in a previous publication (McMullin 2019). One species is listed as Special Concern on the federal Species at Risk Act, *Pseudocyphellariarainierensis* (Government of Canada 2023). We also report four species that are rarely collected in British Columbia: *Opegraphasphaerophoricola*, which is only known from one previous collection in the Province (*Goward 91-692*, UBC); *Protomicarealimosa*, which is reported here for the first time in the Province, even though there is a previous collection from Haida Gwaii (*Brodo 17848*, CANL); *Raesaeneniahuuskonenii*, which is known from a single previous collection in the Province (*Revel 19*, UBC); and *Sareadifformis*, which is also known from a single previous collection (*Björk 20137*, UBC), but ours appears to be the first report from the Province (Allen and McMullin 2021). Five species were previously collected on Calvert Island that we did not find, all collected by Wilfred Schofield: *Cladoniaumbricola*, *Ephebelanata*, *Hypogymniavittata*, *Pseudephebepubescens* and *Umbilicariahyperborea* (see the annotated list for the collector numbers and location of these specimens).

We provide images of 22 of the lichens that we collected on Calvert Island: *Bryoriabicolor*, *Bunodophoronmelanocarpum*, *Caloplacalitoricola*, *Chaenothecopsislecanactidis*, *C.nigripunctata*, *Coccotremamaritimum* (Fig. 2), *Hypogymnialophyrea*, *Lobariaanomala*, *L.anthraspis*, *L.linita*, *L.pulmonaria*, *Normandinapulchella* (Fig. 3), *Opegraphasphaerophoricola*, *Placopsislambii*, *Polycauliona* sp., *Protomicarealimosa*, *Pseudocyphellariarainierensis*, *Psoromahypnorum* (Fig. 4), *Siphulaceratites*, *Sphaerophorustuckermanii*, *Umbilicariadeusta* and *Usnealongissima* (Fig. 5).

## Discussion

We show that bioblitzes are a valuable tool for the rapid appraisal of biodiversity in understudied remote regions like the Central Coast of British Columbia. During our limited time on Calvert Island with only two of us (RTM and AS) collecting specimens, we uncovered 189 species, the vast majority of which had never been reported from Calvert Island. Eight of the species we discovered are either new to British Columbia or are rarely collected in the Province. Some of those rare species (i.e. *Chaenothecopsislecanactidis*, *C.nigripunctata*, *Opegraphasphaerophoricola*, *Protomicarealimosa*, *Raesaeneniahuuskonenii* and *Sareadifformis*) are small and inconspicuous and could simply be overlooked or they might truly be rare species in the region. Four species appear to be novel taxa (*Chaenothecopsis* sp., *Polycauliona* sp., *Trapeliopsis* sp. and *Xylographa* sp.), but additional study is required to describe them. Our results combined with previous collections bring the total number of lichens and allied fungi known on Calvert Island to 194. However, only a small portion of the Island was surveyed and we expect that there are many more species to be discovered, particularly because there are ecosystems and habitats that were not surveyed; for example, around waterfalls or over 300 m (elevation on Calvert Island reaches over 1000 m). The absence of genera (e.g. *Amydalaria*, *Dermatocarpon*, *Leptogium*) that are known from the region also suggests that additional species are likely to be present on the Island.

The nearest detailed lichen biodiversity research in the broader coastal region of British Columbia includes a study in the Gulf Islands and southern part of Vancouver Island in the coastal douglas-fir dry subzone, where 448 lichens were reported (Noble 1982) and an intensive survey of Haida Gwaii, where over 600 lichens are known (I.M. Brodo, unpublished data). These two studies were conducted over many years, covered much larger areas and included a broader range of ecosystems. For example, mountains on Haida Gwaii are above the tree-line, so there are many arctic-alpine lichens present (Thomson 1984, Thomson 1997), but none of the areas that we surveyed on Calvert Island had such conditions. Nevertheless, we did find two arctic-alpine species on exposed coastal rocks (*Brodoaoroarctica* and *Sphaerophorusfragilis*). Noble’s study to the south of Calvert Island has a different climate and ecosystems (e.g. *Quercusgarryana* savannahs), which have different lichen communities (Noble 1982).

Our results include several indicators of old intact forest ecosystems. Selva (2003) developed a calicioid index of ecological continuity that correlates the number of calicioids in an area with the continuity of the forest (i.e. the amount of time that the forest has existed without major disturbance). In the index, the presence of 16 or more calicioid species is considered an indication of an “ancient” forest with a long history of continuity – and we found 16 calicioid species on Calvert Island. Goward and Arsenault (2018) also show that calicioid numbers positively correspond with stand age in the humid inland forests of British Columbia. We found *Pseudocyphellariarainierensis* on Calvert Island as well, which is known to be restricted to old-growth forests in the British Columbia coastal region (COSEWIC 2010, Environment and Climate Change Canada 2017). Goward (1994) also developed a list of old-growth dependent lichens for inland rainforests of British Columbia. Many of the species he lists are on Calvert Island (i.e. *Hypogymniaenteromorpha*, *H.hultenii*, *H.vittata*, *Lobariaoregana*, *Platismatiaherrei*, *P.norvegica*, *Pseudocyphellariacitrina* and *P.hawaiiensis* [as *P.crocata*] and *Sphaerophorustuckermanii*). These lichens show the conservation importance of the intact forests on Calvert Island for old-growth dependent biodiversity.

Bioblitzes are quintessential components of large-scale biodiversity surveys and knowledge acquisition mechanisms. In addition to generating valuable biodiversity data, bioblitzes can contribute to appreciation and curiosity amongst the public for specific groups of lesser-known organisms such as lichens. Unfortunately, lichen identification can be notoriously difficult for beginners; not only is there a wide range of specific vocabulary to learn, but reliable identification often requires microscopy and/or advanced chemical analyses (e.g. thin layer chromatography). Consequently, professional lichenologists are needed to lead these specialised bioblitzes and to publish corresponding annotated checklists for conservation and decision-making purposes. In the case of the 2018 Hakai Terrestrial BioBlitz, the DNA sequences generated required us to further review some specimens which did not fit the reference data for the initially-identified species. We argue that DNA barcoding increases the value of expert-led bioblitzes by facilitating consistent identification of chemically and morphologically overlapping taxa.

## Figures and Tables

**Table 1.** The latitude, longitude and habitat description of the 31 sites surveyed for lichens and allied fungi during the 2018 Hakai Terrestrial BioBlitz. Roman numerals correspond with those Fig. 1.

**Site Number**	**Site Name**	**Coordinates**	**Site Description**
I	Trail between the Hakai Institute Field Station and West Beach, near West Beach	51.65501, -128.13799	Mostly young second-growth mixed-wood forest with a dense canopy. Dominant trees include *Alnusrubra*, *Piceasitchensis*, *Thujaplicata* and *Tsugaheterophylla*.
II	South end of West Beach	51.65285, -128.13873	Coastal rocks/small cliffs and shoreline trees dominated by *Alnusrubra* and *Piceasitchensis*.
III	Central part of West Beach	51.65486, -128.13907	Shoreline stumps, driftwood and trees dominated by *Alnusrubra* and *Piceasitchensis*.
IV	Trail between the Hakai Institute Field Station and West Beach, near field station	51.65514, -128.13243	Mostly young second-growth mixed-wood forest with a dense canopy. Dominant trees include *Alnusrubra*, *Piceasitchensis*, *Thujaplicata* and *Tsugaheterophylla*.
V	Mouth of river valley on east side of Calvert Island	51.62022, -127.93070	Coastal rocks/small cliffs and shoreline trees dominated by *Alnusrubra* and *Thujaplicata*.
VI	River valley on east side of Calvert Island, ca. 350 m west of the coast	51.61978, -127.93576	Sheltered river valley, large old-growth trees dominated by *Alnusrubra*, *Thujaplicata* and *Tsugaheterophylla*.
VII	River valley on east side of Calvert Island, ca. 750 m west of the coast	51.61615, -127.93851	Sheltered river valley, large old-growth trees dominated by *Alnusrubra*, *Thujaplicata* and *Tsugaheterophylla*.
VIII	River valley on east side of Calvert Island, ca. 700 m west of the coast	51.61611, -127.93962	Bog woodland, large trees dominated by *Thujaplicata* and *Tsuga*.
IX	River valley on east side of Calvert Island, ca. 750 m west of the coast	51.61638, -127.94035	Small stream valley with *Alnus*.
X	River valley on east side of Calvert Island, ca. 120 m west of the coast	51.61977, -127.93245	Sheltered river valley, large old-growth trees dominated by *Alnusrubra*, *Thujaplicata* and *Tsuga*.
XI	River valley on east side of Calvert Island, ca. 900 m west of the coast	51.61622, -127.94227	Exposed area, with small pools of standing water and scattered Pinuscontortasubsp.contorta.
XII	East side of Goose Grass Bay	51.66040, -128.11688	Coastal rocks and shoreline trees dominated by *Piceasitchensis* and Pinuscontortasubsp.contorta.
XIII	Central part of Wolf Beach	51.66854, -128.11832	Sand beach with shoreline trees dominated by *Alnusrubra* and *Piceasitchensis*.
XIV	West end of Wolf Beach	51.66797, -128.12128	Coastal rocks/cliffs with shoreline *Piceasitchensis*.
XV	Central area of West Beach, north of trailhead to the Hakai Institute Field Station	51.65616, -128.14052	Coast cliffs in the spray zone.
XVI	Trail between West Beach and North Beach	51.66051, -128.14587	Mature mixed-wood forest dominated by *Alnusrubra*, *Piceasitchensis*, *Thujaplicata* and *Tsugaheterophylla*.
XVII	North Beach	51.66213, -128.14492	Sand beach with shoreline trees dominated by *Alnusrubra* and *Piceasitchensis*.
XVIII	Trail between Goose Grass Bay and Wolf Beach	51.66476, -128.11798	Mature mixed-wood forest dominated by *Thujaplicata*.
XIX	Trail between West Beach and 2^nd^ Beach	51.65065, -128.14241	Mature mixed-wood forest dominated by *Piceasitchensis*, *Thujaplicata* and *Tsugaheterophylla*.
XX	Lookout Point	51.64809, -128.14378	Exposed area, with small pools of standing water, rock outcrops and scattered Pinuscontortasubsp.contorta.
XXI	4^th^ Beach	51.64221, -128.15085	Sand beach with shoreline trees dominated by *Alnusrubra*, *Piceasitchensis* and *Tsugaheterophylla*.
XXII	3^rd^ Beach	51.64502, -128.15099	Shoreline trees dominated by *Alnusrubra* and *Piceasitchensis* and the forest adjacent the beach dominated by *Thujaplicata* and *Tsugaheterophylla*.
XXIII	7^th^ Beach	51.64058, -128.14882	Coastal rocks and shoreline trees dominated by *Alnusrubra* and *Thujaplicata*.
XXIV	Trail between the Hakai Institute Field Station and Tsunami Hill	51.65271, -128.12951	Mature mixed-wood forest dominated by *Thujaplicata* and *Tsuga*.
XXV	Summit area of Tsunami Hill	51.65180, -128.12865	Exposed area with small pools of standing water, rock outcrops and scattered Pinuscontortasubsp.contorta.
XXVI	Shoreline around the dock of the Hakai Institute Field Station	51.65466, -128.13051	Shoreline trees, dominant trees include *Alnusrubra* and *Thujaplicata*.
XXVII	Pruth Bay at the west side of the mouth of Pruth Lagoon	51.65589, -128.13098	Shoreline trees, dominant trees include *Alnusrubra* and *Thujaplicata*.
XXVIII	Forest between Little Wolf and Wolf	51.66425, -128.12706	Coastal western hemlock forest.
XXIX	Little Wolf Beach	51.66391, -128.13013	Open beach, forest margin.
XXX	Telus Towers	51.63722, -128.09525	Open blanket bog.
XXXI	Telus Towers shoreline	51.64512, -128.09463	Coastal rocks and shoreline driftwood.
